# Efficient and Sustainable Electrosynthesis of *N*-Sulfonyl Iminophosphoranes by the Dehydrogenative P–N Coupling Reaction

**DOI:** 10.1021/jacsau.4c00156

**Published:** 2024-04-18

**Authors:** Jessica C. Bieniek, Darryl F. Nater, Sara L. Eberwein, Dieter Schollmeyer, Martin Klein, Siegfried R. Waldvogel

**Affiliations:** † Department of Chemistry, Johannes Gutenberg University Mainz, Duesbergweg 10–14, 55128 Mainz, Germany; ‡ Institute of Biological and Chemical Systems—Functional Molecular Systems (IBCS-FMS), Hermann-von-Helmholtz-Platz 1, 76344 Eggenstein-Leopoldshafen, Germany; § Max-Planck-Institute for Chemical Energy Conversion, Stiftstraße 34–36, 45470 Mülheim an der Ruhr, Germany

**Keywords:** electrochemistry, iminophosphoranes, iodide-mediated, phosphines, sulfonamides

## Abstract

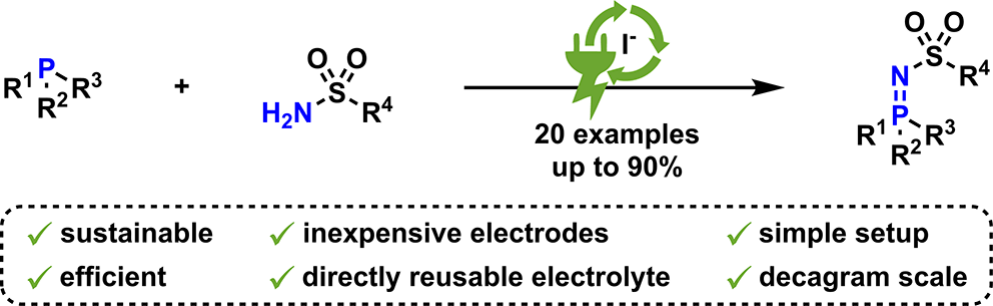

Iminophosphoranes are commonly used reagents in organic synthesis and are, therefore, of great interest. An efficient and sustainable iodide-mediated electrochemical synthesis of *N*-sulfonyl iminophosphoranes from readily available phosphines and sulfonamides is reported. This method features low amounts of supporting electrolytes, inexpensive electrode materials, a simple galvanostatic setup, and high conversion rates. The broad applicability could be demonstrated by synthesizing 20 examples in yields up to 90%, having diverse functional groups including chiral moieties and biologically relevant species. Furthermore, electrolysis was performed on a 20 g scale and could be run in repetitive mode by recycling the electrolyte, which illustrates the suitability for large-scale production. A reaction mechanism involving electrochemical mediation by the iodide-based supporting electrolyte is proposed, completely agreeing with all of the results.

## Introduction

**Scheme 1:**
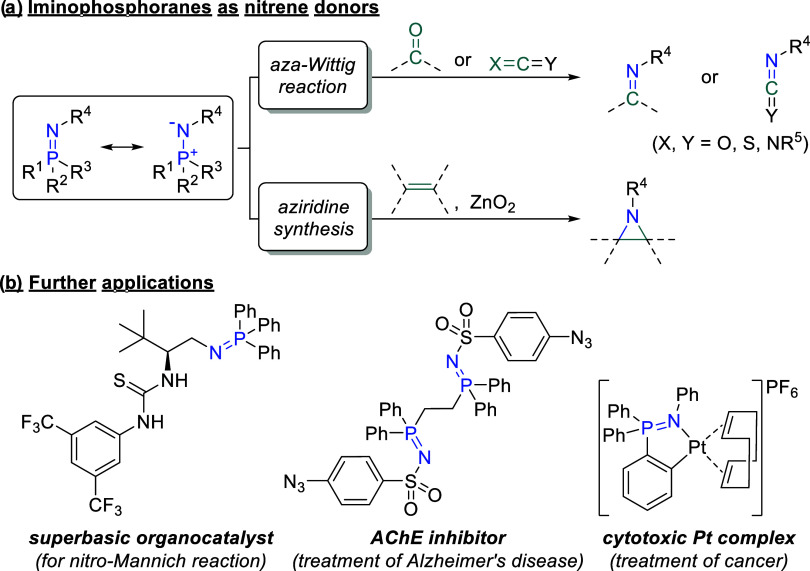
Possible Application Fields for Iminophosphoranes^13,30,33^ AChE = acetylcholinesterase.

Iminophosphoranes, also referred to as phosphinimines, are nitrogen analogues of phosphonium ylides and represent powerful reagents in organic synthesis.^1^ They serve as nitrene donors in aziridine synthesis^2^ and in *aza-Wittig* reactions,^1,3−10^ which are frequently used for the construction of N-heterocyclic compounds such as drugs (Scheme 1a).^8−10^ Furthermore, they are utilized as superbasic organocatalysts for versatile transformations,^11−14^ as ligands for transition metal catalysts,^15−25^ and as intermediates for amino group protection^26^ and optical resolutions.^27,28^ Moreover, several iminophosphoranes showed biological activity and were investigated as potential anticancer agents^29−32^ and acetylcholinesterase (AChE) inhibitors targeting the treatment of Alzheimer’s disease (Scheme 1b).^33^

**Scheme 2:**
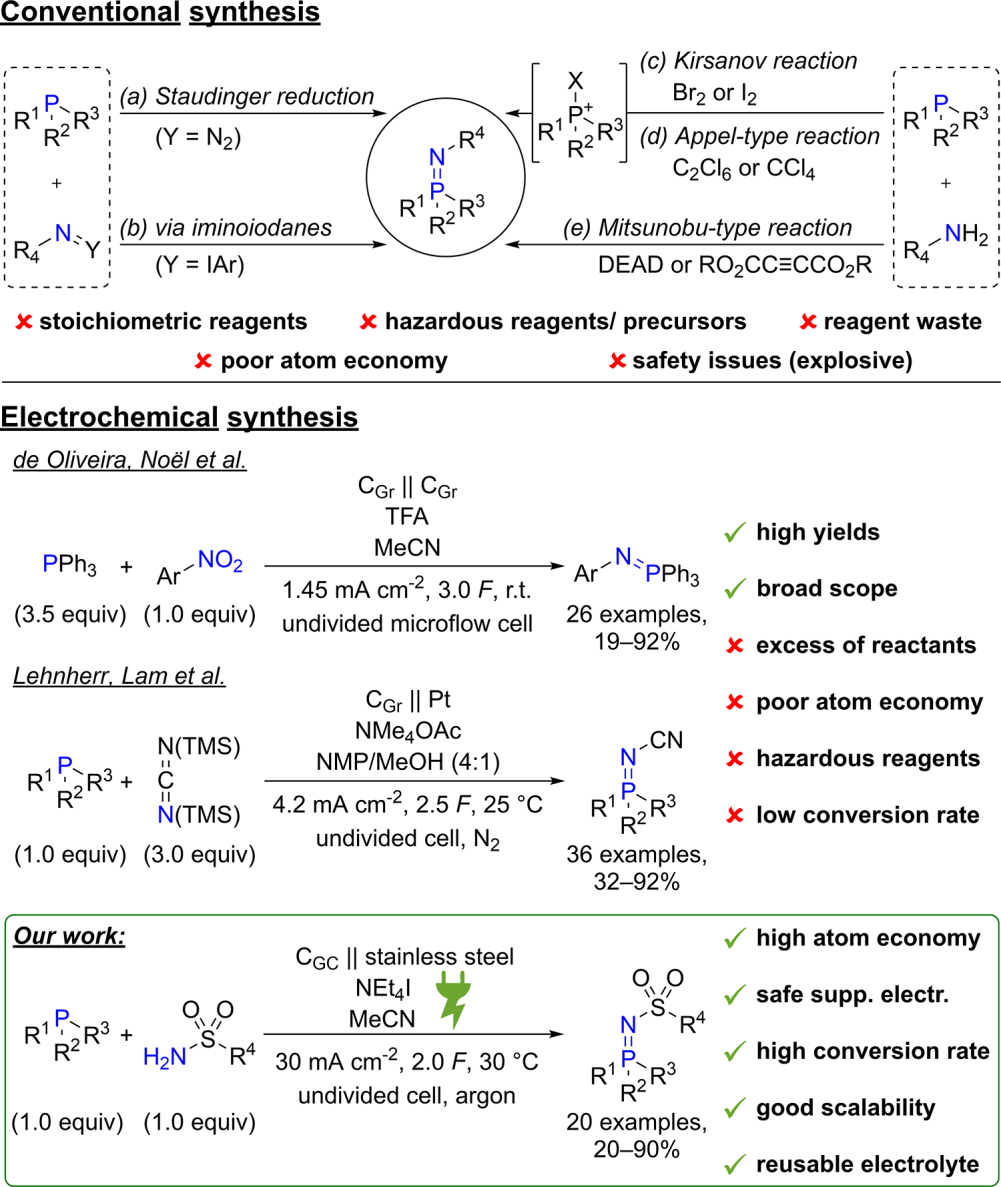
Conventional and Electrochemical Synthesis of Iminophosphoranes Ar = aryl, DEAD = diethyl azodicarboxylate, TFA = trifluoroacetic acid, r.t. = room temperature, TMS = trimethylsilyl, NMP = *N*-methyl-2-pyrrolidone, C_Gr_ = graphite, C_GC_ = glassy carbon, supp. electr. = supporting electrolyte.

Conventionally, iminophosphoranes can be synthesized through *Staudinger* reduction^34−42^ or by reacting phosphines with iminoiodanes (Scheme 2a,b).^43−46^ Alternatively, *Kirsanov*,^47−52^*Appel*-type,^53−55^ or *Mitsunobu*-type^56−62^ reactions can be performed to generate an electrophilic phosphine intermediate in situ, which is then trapped by the nitrogen precursor (Scheme 2c–e).

Despite the broad applicability of these methods, they require toxic and potentially explosive reactants in stoichiometric quantities, which leads to safety risks, as well as large amounts of reagent waste and poor atom economy.

A sustainable and broadly applicable alternative to conventional synthesis approaches is electro-organic synthesis. It employs inexpensive electric current as a traceless redox agent, replacing hazardous and polluting reagents. By this, reagent waste can be minimized, the atom economy can be increased, and costs can be diminished.^63−69^ Furthermore, workup processes can be simplified, since the employed supporting electrolyte and the solvent can often be separated from the formed product by simple aqueous extraction or distillation. Due to the usually high energy demand for workup processes, this plays an essential role and makes elegant combinations of sustainable synthesis and simple workup highly desirable.^63,66^ Finally, work safety is improved, since reactive intermediates are formed in situ in only small amounts, and the reaction can be easily controlled by regulating the electric current.^64−68^

Iminophosphoranes can be synthesized electrochemically by reductive dehydrogenation of (alkylamino)phosphonium salts^70^ or by P–N coupling reactions of a phosphine and a nitrogen compound.^71,72^ The latter have so far only been reported by de Oliveira and Noël et al.,^71^ as well as by Lehnherr and Lam et al.^72^ who synthesized *N*-aryl iminophosphoranes from nitro arenes and *N*-cyano iminophosphoranes from bis(trimethylsilyl)carbodiimide, respectively (Scheme 2). These methods allow for the synthesis of numerous derivatives in good to excellent yields. However, both methods require one of the precursors to be used in large excess (3.0–3.5 equiv), resulting in additional reagent waste and poor atom economy. Furthermore, hazardous trifluoroacetic acid (TFA) and *N*-methyl-2-pyrrolidone (NMP) are used within the electrolyte system, leading to safety issues. Lastly, conversion rates are low due to relatively low current densities (1.45 and 4.2 mA cm^–2^), making large-scale applications less attractive.

In contrast, we herein present a sustainable, inherently safe, efficient, and easily scalable synthesis of *N*-sulfonyl iminophosphoranes, starting from readily available phosphines and sulfonamides by applying inexpensive electrode materials. This method features a broad and diverse scope, high conversion rates, and an excellent atom economy.

## Results and Discussion

The reaction conditions for the electrochemical formation of iminophosphoranes were optimized by using triphenylphosphine (PPh_3_, **1**) and *p*-toluenesulfonamide (TsNH_2_, **2**) as test substrates in equimolar amounts. Detailed information about the reaction optimization is provided in the Supporting Information. Initially, PPh_3_ and TsNH_2_ were electrolyzed under constant current conditions in an undivided cell equipped with a glassy carbon anode and a nickel cathode. The reaction was performed under air, and a mixture of NEt_4_I, *tert*-butanol, and acetonitrile was employed as the electrolyte. The choice of NEt_4_I as a supporting electrolyte was based on previous reports, where halide-based supporting electrolytes proved to be suitable redox mediators for various P–N coupling reactions^73−80^ and for the analogue sulfilimine synthesis.^81^ By applying the initial conditions, iminophosphorane **3** was obtained in 33% ^31^P NMR yield, while triphenylphosphine oxide (TPPO) was formed as a major product in 59% yield (Table 1, entry 1). In order to avoid TPPO formation, the reaction was repeated by applying an inert atmosphere and anhydrous acetonitrile (Table 1, entry 2). With this, the yield of product **3** could be raised to 50% and TPPO formation could be reduced to 28%, demonstrating the importance of inert conditions for an improved reaction performance. When various supporting electrolytes were screened under inert and ambient conditions, NEt_4_I turned out to be superior to the other tested supporting electrolytes and was therefore further used (see the Supporting Information). Next, various additives were investigated. The absence of additives led to a slightly lower yield for **3** (42%, Table 1, entry 3). A significantly higher yield of 73% was obtained with 1,1,1,3,3,3-hexafluoro-2-propanol (HFIP, 0.10 m) as an additive, exhibiting unique solvation properties while being poorly nucleophilic (Table 1, entry 4).^82−85^ With a higher HFIP concentration of 0.15 m, product **3** yield increased to 91%, which did not significantly change at higher HFIP concentrations (Table 1, entry 5, see the Supporting Information). Therefore, HFIP at a concentration of 0.15 m was further used as the additive. When screening cathode materials, nickel could be replaced by stainless steel, being advantageous in terms of costs, availability, and safety, leading to 87% of **3** (Table 1, entry 6). Regarding the anode material, isostatic graphite provided a lower product yield compared to glassy carbon, which was therefore further used (Table 1, entry 7). Next, the interelectrode gap and the current density were investigated. With a smaller interelectrode gap of 4 mm instead of 9 mm, the cell voltage and thus the specific energy consumption of the reaction could be lowered by around 40% from 2.12 to 1.23 kJ mmol^–1^ while keeping the yield of **3** on a high level of 90% (Table 1, entry 8). Regarding the current density, no significant change in yield was observed within a range between 10 and 50 mA cm^–2^, demonstrating the high robustness of this transformation toward fluctuations in current density (see the Supporting Information). This will be particularly important for future industrial applications in order to adapt flexibly to intermittent energy, as expected with electricity generated from renewable energy sources.^81,86^ To maintain a high space-time yield while increasing the energy efficiency, a current density of 30 mA cm^–2^ was chosen, resulting in a yield for **3** of 91% and a specific energy consumption of 0.87 kJ mmol^–1^ (Table 1, entry 9). The efficiency and sustainability of the reaction could further be improved by tripling the concentration of the starting material (*c*_s.m._), which led to the highest product yield of 95% while lowering the amounts of reagents and solvent needed relative to the amount of generated product (Table 1, entry 10). With these conditions, the HFIP concentration was finally screened. Interestingly, **3** was formed in a very good yield of 89% in the absence of HFIP, indicating no significant influence of HFIP on the reaction performance (Table 1, entry 11, see the Supporting Information). The energy demand remained at a low level of 0.92 kJ mmol^–1^. Since the omission of highly fluorinated and persistent organics, such as HFIP, improves the sustainability of the reaction dramatically, these conditions were chosen to be the appropriate ones, despite a slightly lower product yield.

**Table 1:**
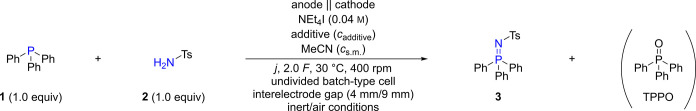
Optimization of the Reaction Conditions for the Synthesis of Iminophosphorane **3**^a^

entry	inert/air	additive	*c*_additive_ (m)	anode	cathode	interelectrode gap (mm)	*j* (mA cm^–2^)	*c*_s.m._ (m)	**3**^b^ (%)	TPPO^b^ (%)	**1**^b^,^c^ (%)
1^d^	air	^*t*^BuOH	0.10	C_GC_	Ni	9	50	0.1	33	59	6
2	inert	^*t*^BuOH	0.10	C_GC_	Ni	9	50	0.1	50	28	9
3	inert			C_GC_	Ni	9	50	0.1	42	19	8
4	inert	HFIP	0.10	C_GC_	Ni	9	50	0.1	73	18	0
5	inert	HFIP	0.15	C_GC_	Ni	9	50	0.1	91	5	1
6	inert	HFIP	0.15	C_GC_	stainless steel	9	50	0.1	87	8	1
7	inert	HFIP	0.15	C_Gr_	stainless steel	9	50	0.1	78	19	0
8	inert	HFIP	0.15	C_GC_	stainless steel	4	50	0.1	90	7	1
9	inert	HFIP	0.15	C_GC_	stainless steel	4	30	0.1	91	6	0
10	inert	HFIP	0.15	C_GC_	stainless steel	4	30	0.3	95	4	0
11	inert			C_GC_	stainless steel	4	30	0.3	89	5	1

a Triphenylphosphine (PPh_3_, **1**, 1.0 equiv, 1.5 mmol for *c*_s.m._ = 0.1 m, 4.5 mmol for *c*_s.m._ = 0.3 m), *p*-toluenesulfonamide (TsNH_2_, **2**, 1.0 equiv, 1.5 mmol for *c*_s.m._ = 0.1 m, 4.5 mmol for *c*_s.m._ = 0.3 m), NEt_4_I (0.04 m, 0.6 mmol), additive (*c*_additive_) in acetonitrile (MeCN, 15 mL), undivided 25 mL batch-type glass cell, anode, cathode, interelectrode gap (possible settings: 4 mm/9 mm), current density *j* (active anodic surface: 4.8 cm^2^), applied molar charge: 2.0 *F*, 30 °C, stirring speed: 400 rpm, inert (argon atmosphere, anhydrous acetonitrile) or air (air atmosphere, HPLC grade acetonitrile) conditions. Abbreviations: s.m. = starting material, TPPO = triphenylphosphine oxide, HFIP = 1,1,1,3,3,3-hexafluoro-2-propanol, C_GC_ = glassy carbon, C_Gr_ = isostatic graphite.

b Yields determined by ^31^P NMR spectroscopy using triphenyl phosphate as an internal standard.

c Residual PPh_3_ (**1**).

d Active anodic surface: 5.6 cm^2^ due to different stirring bars.

**Scheme 3:**
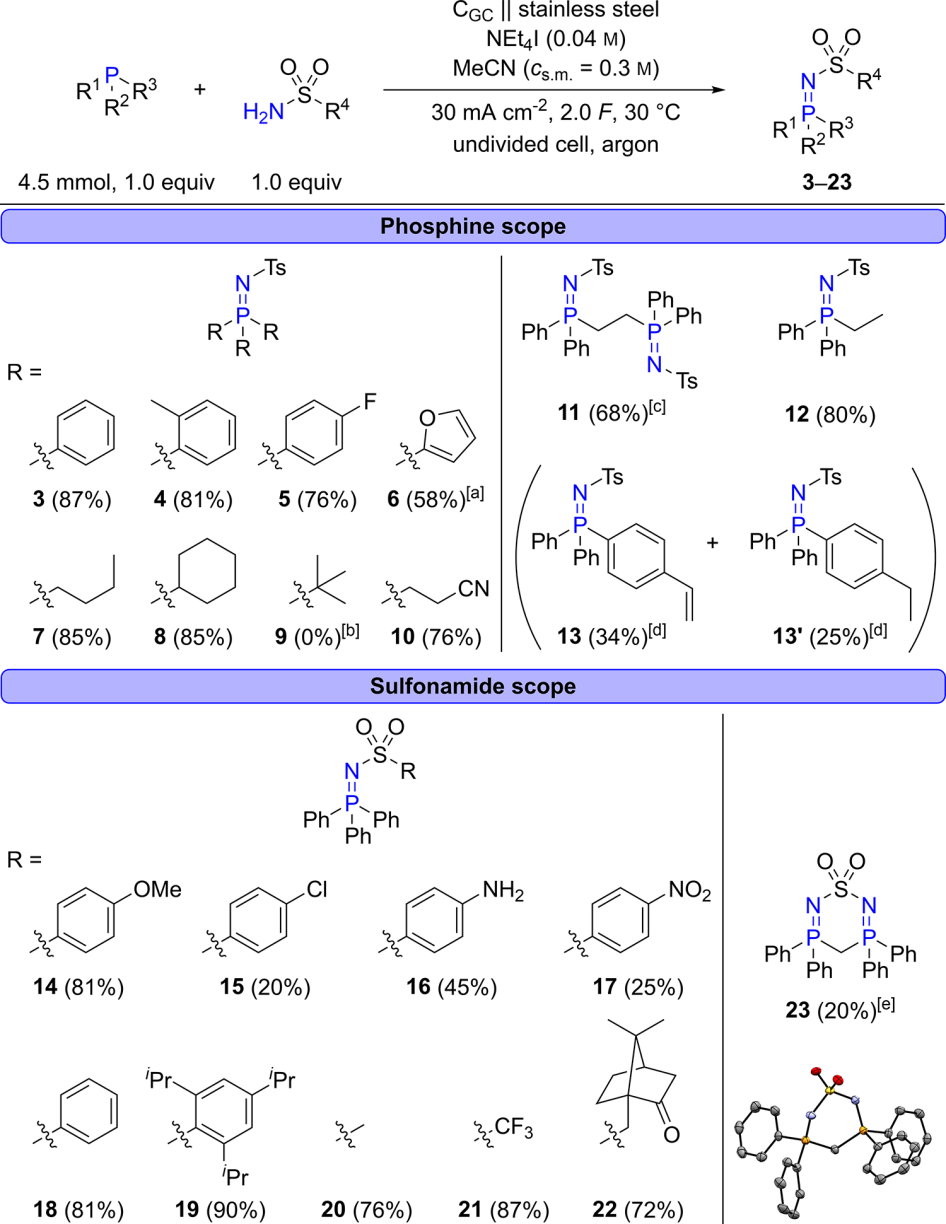
Reaction Scope Based on Diverse Phosphines and Sulfonamides 2.4 *F*. Tri-*tert*-butylphosphine (3.1 mmol, 1.0 equiv) and TsNH_2_ (3.1 mmol, 1.0 equiv). Smaller amounts of substrates due to poor solubility: diphosphine (1.5 mmol, 1.0 equiv), TsNH_2_ (3.0 mmol, 2.0 equiv), 4 *F*, 500 rpm. Both products were obtained from 4-(diphenylphosphino)styrene. 4 *F*. Phosphine (4.5 mmol, 1.0 equiv), sulfonamide (4.5 mmol, 1.0 equiv), NEt_4_I (0.6 mmol) in anhydrous acetonitrile (15 mL), argon atmosphere, undivided 25 mL glass cell, glassy carbon (C_GC_) anode, stainless steel cathode, interelectrode gap: 4 mm, 30 mA cm^–2^ (active anodic surface: 4.8 cm^2^), 2.0 *F*, 30 °C, stirring speed: 400 rpm. All yields are isolated yields.

Subsequently, the scope of the reaction was investigated (Scheme 3). First, diverse phosphines were reacted with TsNH_2_. Test derivative **3** could be isolated in 87% yield. A similar result was obtained for **4** involving three electron-rich and sterically demanding *o*-tolyl substituents on the phosphorus atom. Compound **5** with three electron-deficient 4-fluorophenyl moieties could also be synthesized in a good yield of 76%. In contrast, tris(pentafluorophenyl)phosphine failed to provide the corresponding iminophosphorane when electrolyzed with TsNH_2_. Styrene substituents turned out to be tolerated in the reaction, and 4-(diphenylphosphino)styrene could be converted to the expected product **13** in 34% yield. Interestingly, hydrogenated byproduct **13′** was formed in 25% yield. Moreover, **6** bearing three 2-furyl substituents could be synthesized in 58% yield, confirming that aryl- and heteroaryl-substituted phosphines are well tolerated in this transformation. Next, alkyl-substituted phosphines were tested. Electron-rich phosphines with primary and secondary alkyl substituents, such as *n*-butyl and cyclohexyl groups, could be converted into corresponding iminophosphoranes **7** and **8** in a very high yield of 85%. In contrast, **9**, wherein the phosphine bears three *tert*-butyl substituents, was not accessible by this method, likely due to the high steric hindrance caused by the substituents. Electron-withdrawing cyanoethyl groups were tolerated as well in the reaction, providing **10** in 76% yield, which is viable for further functionalization. Further, unsymmetrical iminophosphorane **12** could be isolated in a high yield of 80%, illustrating that both aryl- and alkyl-substituted phosphines are suitable for the reaction. Lastly, diiminophosphorane **11**, which is an interesting ligand for organometallic complexes,^16,22^ was accessible in 68% yield by reacting 1,2-bis(diphenylphosphino)ethane (dppe) with 2 equiv of TsNH_2_.

Next, the sulfonamide scope was investigated. The electron-rich 4-methoxybenzenesulfonamide could be converted successfully into iminophosphorane **14** in a good yield of 81%. Conversely, **15** with a *p*-chlorophenyl substituent was obtained in a poor yield of 20%. Sulfanilamide, which was formerly used as an antibiotic,^87^ yielded **16**(88) in 45% yield. Pleasingly, the unprotected amino group was tolerated in the reaction, which tends to be challenging under oxidative conditions.^89−91^ Moreover, redox-sensitive nitro groups were tolerated in the reaction, as is evident from **17**, which was isolated in a 25% yield. Benzenesulfonamide with an unsubstituted phenyl group yielded **18** in 81%. The highest yield was obtained for iminophosphorane **19**, which was formed in excellent 90%, despite bearing two sterically demanding isopropyl groups in the *o*-position of the phenyl group. Alkyl sulfonamides provided corresponding derivatives **20** and **21** in good yields of 76 and 87%, respectively, demonstrating that both electron-rich and electron-deficient alkyl substituents on the sulfonamide moiety are tolerated in this reaction. Furthermore, chiral iminophosphorane **22** could be prepared from enantiopure (1*S*)-10-camphorsulfonamide in 72% yield, showing that intermediates for chiral transformations are easily accessible by this method. Finally, 1,1-bis(diphenylphosphino)methane was reacted with sulfamide, yielding heterocycle **23**(88) in 20% by a 2-fold P=N bond formation.

**Figure 1:**
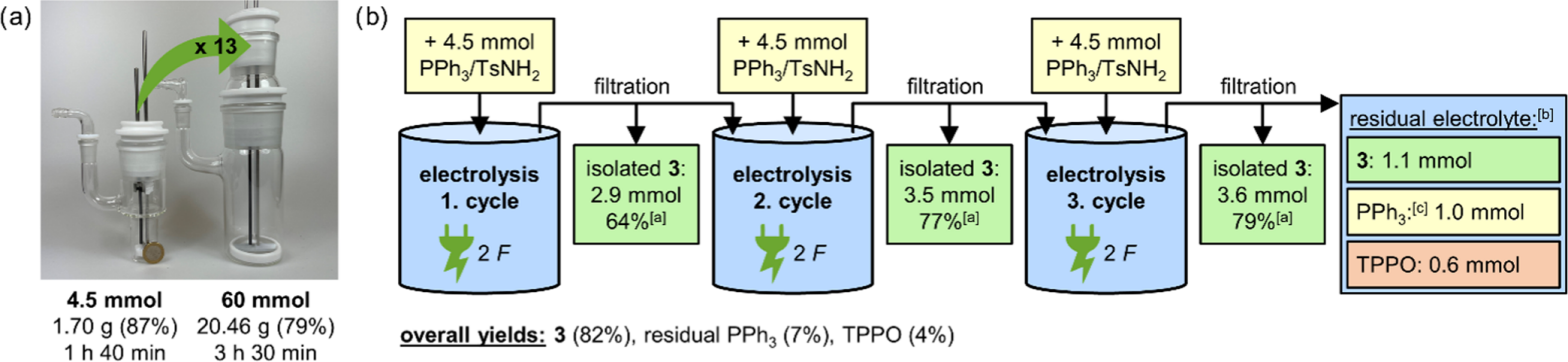
(a) Scale-up of the electrolysis reaction with PPh_3_ and TsNH_2_ from 4.5 to 60 mmol scale (left: 25 mL glass cell; right: 200 mL glass cell, in comparison to a 1 € coin). Standard reaction conditions with 2.1 *F* were applied (active anodic surface: 32.4 cm^2^). Amounts of isolated **3**, reaction yields, and reaction times are compared. A 12-fold amount of **3** (20.46 g vs 1.70 g) was synthesized in only double the time (3 h 30 min vs 1 h 40 min). (b) Electrolysis of PPh_3_ and TsNH_2_ in repetitive mode in 25 mL glass cells. Standard reaction conditions were applied. After each electrolysis (2 *F*), precipitated **3** was filtered off, and the residual electrolyte was enriched with the additional starting material (4.5 mmol of PPh_3_ and TsNH_2_ each) and electrolyzed again. A total of three electrolysis cycles was conducted reusing the electrolyte. Prior to the third electrolysis, additional acetonitrile (5 mL) was added due to losses during filtration. [a] Isolated **3** after filtration. Yield is calculated based on 4.5 mmol of the starting material. [b] The composition of the residual electrolyte after three electrolysis cycles was analyzed by ^31^P NMR, using triphenyl phosphate as an internal standard. [c] Nonconverted PPh_3_.

In order to demonstrate the suitability of the established method for production on a larger scale, electrolysis of PPh_3_ (**1**) and TsNH_2_ (**2**) was performed on a 60 mmol scale, which corresponds to a 13-fold scale-up (Figure 1a). The electrolysis was conducted in a 200 mL glass cell, applying standard conditions (with 2.1 *F*) and two electrodes in the size of 4 × 12 × 0.3 cm^3^, with an active anodic surface area of 32.4 cm^2^. With this setup, 20.46 g of **3** could be generated within 3.5 h in a good yield of 79%. The workup turned out to be straightforward, since most of the formed product (14.72 g) precipitated from the electrolyte and could simply be filtered off. In addition, 88% of employed NEt_4_I could be recovered by an aqueous extraction.

These results prove that the reaction is readily scalable, since a 12-fold amount of **3** was produced in only double the time, resulting in a 6-fold higher productivity. Furthermore, it was investigated if the reaction is operable in a repetitive way by directly reusing the electrolyte and feeding it with the substrate. For this, the electrolysis of **1** and **2** was run on a small scale (4.5 mmol) under standard conditions. Then, precipitated **3** was filtered off, and the residual electrolyte was enriched with the additional starting material (4.5 mmol of **1** and **2** each) and electrolyzed again applying 2 *F*. This procedure was repeated for three electrolysis cycles. During this experiment, 1.25 g (2.9 mmol), 1.49 g (3.5 mmol), and 1.54 g (3.6 mmol) of pure **3** were isolated by filtration after the first, second, and third electrolysis cycles, respectively. This corresponds to reaction yields of 64, 77, and 79% relative to 4.5 mmol of the starting material. Additionally, the residual electrolyte contained 1.1 mmol of **3** (determined by ^31^P NMR), which resulted in an overall product yield of 82% over three electrolysis cycles. Besides this, 7% of nonconverted PPh_3_ was left, and only 4% of TPPO was formed over the three cycles. These results demonstrate impressively that the electrolyte can be efficiently reused and that repetitive operation of this electrolysis is feasible by feeding the substrate while removing the precipitated product.

**Scheme 4:**
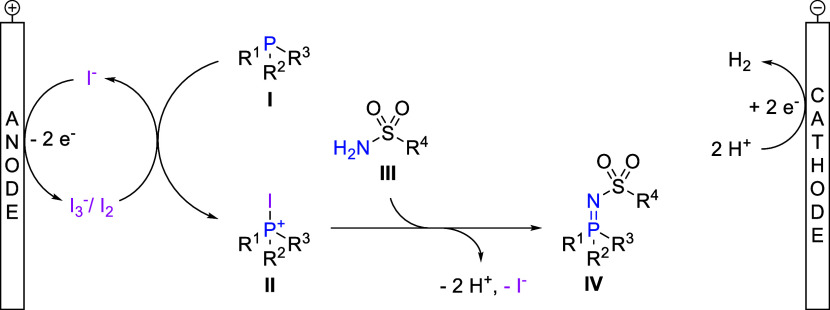
Proposed Reaction Mechanism Based on CV Measurements

To obtain insights into the reaction mechanism, cyclic voltammetry (CV) measurements were performed (see the Supporting Information). Based on these results, a reaction mechanism is proposed in which the iodide-based supporting electrolyte fulfills a dual role, serving as an ionic conductor while simultaneously acting as a redox mediator (Scheme 4).^92^ First, iodide is oxidized at the anode to form I_2_ and I_3_^–^. Then, phosphine **I** reacts with I_2_ equivalent to form intermediate **II**, analogously to Kirsanov reactions.^48,51,52^ This hypothesis is supported by a control experiment in which I_2_ (1.0 equiv) instead of electric current was employed as the oxidizing agent (see the Supporting Information). Nucleophilic attack by sulfonamide **III**, followed by elimination of iodide and deprotonation, yields iminophosphorane **IV**. At the cathode, hydrogen is evolved from the liberated protons.

## Conclusions

In summary, an efficient and sustainable electrochemical synthesis of *N*-sulfonyl iminophosphoranes with high atom economy has been established. The reaction protocol involves the use of a simple galvanostatic setup, inexpensive and readily available electrode materials, small amounts of NEt_4_I as inherently safe supporting electrolytes, high conversion rates, and good energy efficiency. This method was applied to synthesize 20 examples in yields up to 90%, bearing various functional groups including chiral moieties and biologically relevant species. The large-scale applicability of the reaction was demonstrated by performing the electrolysis on a 60 mmol scale (20 g) and by running it in a repetitive way reusing the electrolyte. Finally, an iodide-mediated reaction mechanism was proposed based on CV studies.

## Supporting Information Available

Detailed experimental procedures; characterization data; cyclic voltammetry data and crystal structure data; and detailed results of reaction screening (PDF)
