# Supplementary material for: Efficient and Sustainable Electrosynthesis of N-Sulfonyl Iminophosphoranes by the Dehydrogenative P–N Coupling Reaction
Source: JACS Au. 2024 Apr 18;4(6):2188–96. doi: 10.1021/jacsau.4c00156 (PMC11200248; doi:10.1021/jacsau.4c00156)
Supplement: Supplementary file 1 — au4c00156_si_001.pdf [file au4c00156_si_001.pdf]

Supporting Information

Efficient and Sustainable Electrosynthesis of *N*-Sulfonyl Iminophosphoranes by Dehydrogenative P-N Coupling Reaction

Jessica C. Bieniek,+[a] Darryl F. Nater,+[a,c] Sara L. Eberwein,[a] Dieter Schollmeyer,[a] Martin Klein[a] and Siegfried R. Waldvogel*[a,b,c]

[a] Department of Chemistry, Johannes Gutenberg University Mainz, Duesbergweg 10–14, 55128 Mainz (Germany)

[b] Institute of Biological and Chemical Systems – Functional Molecular Systems (IBCS-FMS), Hermann-von-Helmholtz-Platz 1, 76344 Eggenstein-Leopoldshafen (Germany)

[c] Max-Planck-Institute for Chemical Energy Conversion, Stiftstraße 34–36, 45470 Mülheim an der Ruhr (Germany)
E-Mail: siegfried.waldvogel@cec.mpg.de
Homepage: www.cec.mpg.de/en/home

+ The authors contributed equally to this work.

Table of Contents

1 General Information S3
1.1 Instruments and Analytical Methods S3
1.2 Electrolysis Set-up S3
2 Electrolysis Protocols S5
2.1 General Protocol A: Electrolysis in Undivided 25 mL Batch-Type Glass Cells Applying Optimized Conditions S5
2.2 General Protocol B: Optimization Experiments in Undivided 25 mL Batch-Type Glass Cells S5
2.3 General Protocol C: Optimization Experiments in Undivided 5 mL Teflon™ Screening Cells S5
2.4 Scale-up of the Reaction: 60 mmol Scale in Undivided 200 mL Batch-Type Glass Cell S6
2.5 Electrolysis in Repetitive Mode by Reuse of the Electrolyte and Feeding of Starting Material S6
3 Optimization Data S7
3.1 Optimization Experiments in Undivided 5 mL Teflon™ Screening Cells S7
3.2 Optimization Experiments in Undivided 25 mL Beaker-Type Glass Cells S7
4 Cyclic Voltammetry Studies and Mechanistic Considerations S12
5 Limitations S15
6 Compound Characterization S16
6.1 Starting Materials S16
6.2 *N*-Sulfonyl iminophosphoranes S16
7 NMR Spectra S22
8 Crystallographic Data S57
9 References S61

1 General Information

All reagents were used in analytical grades and were obtained from common commercial sources. Cyclohexane and ethyl acetate (technical grade) were distilled prior to use. Other solvents were used in analytical grades. Anhydrous acetonitrile was obtained by degassing HPLC grade acetonitrile (alternating application of vacuum and argon), followed by drying over activated molecular sieve (3 Å) for at least four days.

1.1 Instruments and Analytical Methods

Column Chromatography: Automated preparative chromatography was performed using a puriFlash™ XS 520 Plus (Interchim, Montluçon, France), applying a prepacked puriFlash™ SI-HP silica gel PF-15SIHP-F0040 column, a puriFlash™ SI-HP silica gel PF-30SIHP-F0040 column or a puriFlash™ SI-HC silica gel PF-25SIHC-F0080 column (Interchim, Montluçon, France). Cyclohexane and ethyl acetate were used as eluents. Silica gel 60 sheets on aluminum (F254, Merck KGaA, Darmstadt, Germany) were employed for thin layer chromatography. Reversed phase column chromatography was performed with a prepacked Sepacore™ C18 column (Büchi-Labortechnik GmbH, Essen, Germany), using a preparative chromatography system (Büchi-Labortechnik GmbH, Essen, Germany) with a Büchi Control Unit C-620, an UV detector Büchi UV photometer C-635, a Büchi fraction collector C-660 and two Pump Modules C-605 for adjusting the solvent mixtures. Mixtures of water (MilliQ™) and acetonitrile were used as eluents.

High Performance Liquid Chromatography (HPLC): Analytical HPLC was performed on a Shimadzu HPLC-MS system (Shimadzu, Kyoto, Japan) using an autosampler SIL-20AHT, a column oven CTO-20AC, two pump modules LC-20AD for adjusting the solvent mixtures, a diode array detector SPD-M20A, a communication BUS Modul CBM-20A and an Eurospher II 100-5 C18 column 150 mm x 4 mm (Knauer, Berlin, Germany). HPLC-MS measurements were carried out on a LCMS-2020 (Shimadzu, Kyoto, Japan). Acetonitrile (HPLC-MS grade) and water (MilliQ™) with 0.1% (*v*/*v*) formic acid were used as eluents.

Spectroscopy and Spectrometry: ^{1}H NMR, ^{13}C NMR, ^{19}F NMR, ^{31}P NMR and 2D NMR spectra were measured at 25 °C, using a Bruker Avance II 400, a Bruker Avance III HD 400, or a Bruker Avance III 600 spectrometer (Bruker BioSpin GmbH, Rheinstetten, Germany). Chemical shifts (δ) are reported in parts per million (ppm) relative to traces of $CHCl_3$, CH_2Cl_2 or CH_3CN in the corresponding deuterated solvent. The ^{31}P NMR spectra were recorded using a ^{31}P NMR standard method with a normal ^{1}H decoupling or a ^{31}P NMR inverse gated method with an inverse gated ^{1}H decoupling. Phosphoric acid (5% H_3PO_4 in D_2O) served as external standard. For quantification by ^{31}P NMR spectroscopy triphenyl phosphate was used as internal standard. The ^{19}F spectra were recorded without ^{1}H decoupling, and α-trifluorotoluene served as external standard. High-resolution mass spectra were measured using an Agilent 6545 QTOF-MS (Agilent, Santa Clara, USA) apparatus employing ESI+.

X-Ray Analysis: All data were collected on a STOE IPDS2T diffractometer with Oxford Cryostream 700er series cooling device (Oxford Cryosystems, Oxford, United Kingdom) using graphite monochromated Mo Kα radiation (λ = 0.71073 Å). Intensities were measured using fine-slicing ω-scans and corrected for background, polarization, and Lorentz effects. The structures were solved by direct methods and refined anisotropically by the least-squares procedure implemented in the SHELXL-2019 program system.[1] The supplementary crystallographic data for this paper can be obtained free of charge from The Cambridge Crystallographic Data Centre (www.ccdc.cam.ac.uk/data_request/cif). Deposition numbers and further details are given with the individual characterization data.

Cyclic Voltammetry (CV) Measurements: CV measurements were performed with a Metrohm Autolab type III potentiostat (Metrohm AG, Herisau, Switzerland). Working electrode (WE): glassy carbon (diameter: 2 mm), counter electrode (CE): glassy carbon rod (diameter: 2 mm), reference electrode (RE): Ag/AgCl.

1.2 Electrolysis Set-up

DC Power Supply:
A Rohde & Schwartz HMP4040 programmable power supply (Rhode & Schwartz, Munich, Germany) was used. All electrolyses were conducted under galvanostatic conditions.

Undivided 5 mL Teflon™ Screening Cells:
Initial electrolysis experiments were conducted in undivided 5 mL Teflon™ screening cells under air, with two electrodes (70 x 10 x 3 mm^{3}) in a parallel orientation.[2] The cells were fixed in an aluminum block, which was placed onto a magnetic stirrer (IKA™ RCT basic IKAMAG™ safety control, IKA-Werke GmbH & Co. KG, Staufen, Germany). Heating occurred with the aluminum arrangement, applying a contact thermometer connected to the magnetic stirrer. This screening set-up is also commercially available as IKA Screening System (IKA-Werke GmbH & Co. KG, Staufen, Germany).[3]

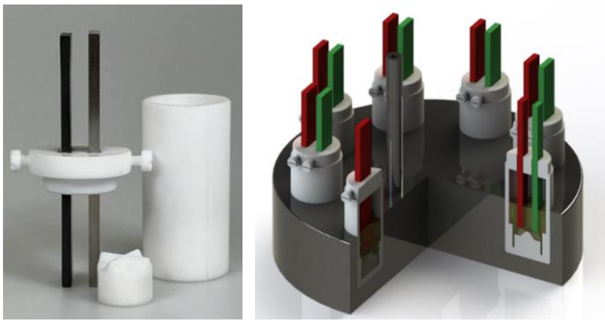

Figure S1: Undivided 5 mL Teflon™ screening cell with a stirrer, and two electrodes (70 x 10 x 3 mm³) (left). Schematic screening arrangement, consisting of undivided 5 mL Teflon™ screening cells arranged in an aluminum block (right).[2]

Undivided 25 mL Batch-Type Glass Cells:

Electrolysis reactions according to general protocols **A** and **B** were performed in undivided 25 mL batch-type glass cells, with two electrodes (60 x 20 x 3 mm^3) in parallel orientation. The electrodes were fixed within the PTFE stopper using electrode holders made from stainless-steel. Possible inter-electrode gaps were 4 mm or 9 mm. The electrode holders were insulated from each other with Teflon™ tape. For reactions under inert conditions an argon stream was applied through the side outlet of the cell. Two different stirring bars (Figure S2) were used, resulting in different anodic surface areas exposed to the electrolyte. With 15 mL of solvent the active anodic surface area for stirring bar SB1 was 5.6 cm^2, and for stirring bar SB2 was 4.8 cm^2. Heating occurred through a water bath.

Undivided 200 mL Batch-Type Glass Cell:

The scale-up experiment was performed in an undivided 200 mL batch-type glass cell, applying two electrodes (120 x 40 x 3 mm^3) in parallel orientation. The electrodes were fixed within the PTFE stopper using stainless-steel electrode holders insulated with Teflon™ tape, applying an inter-electrode gap of 4 mm. An additional glass elongation part was used to fit the electrodes within the cell. Argon was applied through the side outlet of the cell. Heating occurred through a water bath. A comparable 200 mL glass batch-type cell with a PTFE stopper and electrode holders is commercially available as SynLectro™ Starter Kit (Merck KGaA, Darmstadt, Germany).[4]

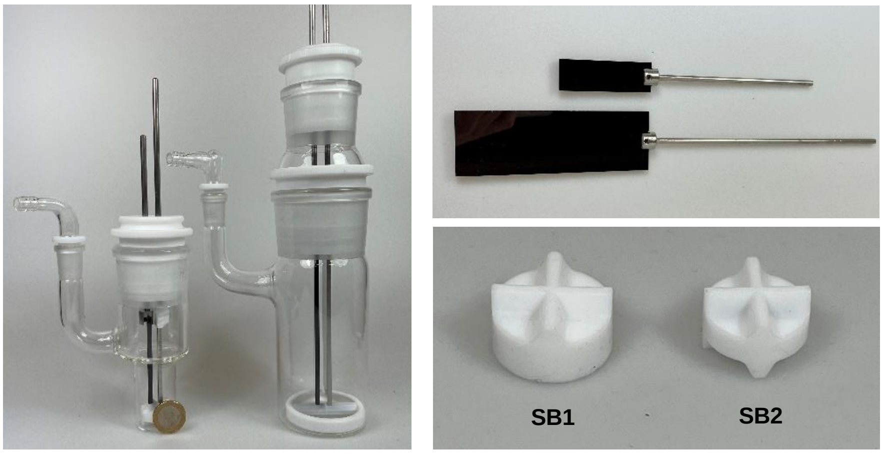

Figure S2: Undivided 25 mL batch-type glass cell and 200 mL batch-type glass cell with PTFE stoppers, stirring bars, electrode holders and electrodes, compared to a 1 € coin (left). Glassy carbon electrodes (SIGRADUR™ G, 60 x 20 x 3 mm^3 and 120 x 40 x 3 mm^3) in electrode holders (top right). Stirring bars SB1 and SB2, used for electrolysis experiments in 25 mL batch-type glass cells (bottom right).

Electrode Materials:

The following electrode materials were employed in electrolysis experiments. Isostatic graphite, stainless-steel and nickel electrodes were sanded prior to use.

Isostatic graphite: Highly isostatic graphite SIGRAFINE™ V2100 was obtained from SGL Carbon, Bonn, Germany.

Glassy carbon: Glassy carbon electrodes SIGRADUR™ G and glassy carbon rods SIGRADUR™ G were obtained from HTW GmbH, Thierhaupten, Germany.

Stainless-steel: Stainless-steel (VA 1.4571) electrodes were machined from a bigger piece.

Nickel: Nickel electrodes were obtained from IKA Werke GmbH & Co. KG, Staufen, Germany. The geometries were machined from a larger piece.

Platinum: Platinum electrodes were obtained from ÖGUSSA Ges.m.b.H., Wien, Austria.

2 Electrolysis Protocols

2.1 General Protocol A: Electrolysis in Undivided 25 mL Batch-Type Glass Cells Applying Optimized Conditions

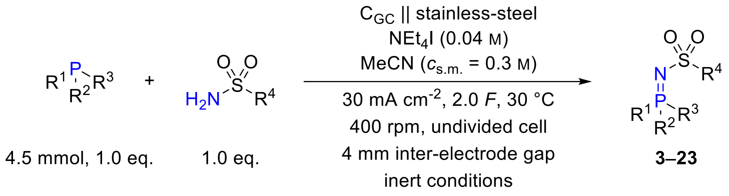

The undivided 25 mL batch-type glass cell, equipped with a glassy carbon anode and a freshly sanded stainless-steel cathode (inter-electrode gap: 4 mm), was flushed with argon for 15 minutes. Then, the phosphine (4.5 mmol, 1.0 eq.), sulfonamide (4.5 mmol, 1.0 eq.), tetraethylammonium iodide (NEt_4I, 0.154 g, 0.6 mmol), and anhydrous acetonitrile (15 mL) were added, and the resulting mixture was stirred (stirring bar SB2, stirring speed v_{stirr} = 400 rpm) under argon atmosphere at 30 °C for 30 minutes. Afterwards, the reaction mixture was electrolyzed, applying constant current conditions (current density j = 30 mA cm^{-2}, active anodic surface: 4.8 cm^2), and a total applied charge of 2.0 *F*. After completed electrolysis, the reaction mixture was concentrated, water (50 mL) was added, and the aqueous layer was extracted with dichloromethane (3 x 50 mL). The combined organic fractions were dried over anhydrous magnesium sulfate and the solvent was removed under reduced pressure. If not stated otherwise, the crude product was purified by column chromatography, yielding the corresponding iminophosphoranes **3**–**23**.

2.2 General Protocol B: Optimization Experiments in Undivided 25 mL Batch-Type Glass Cells

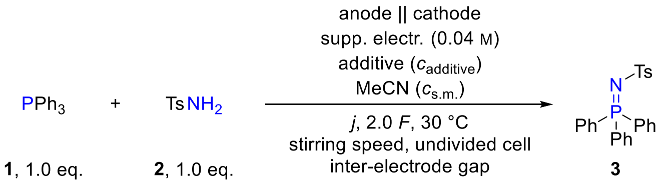

A mixture of triphenylphosphine (1.0 eq.), *p*-toluenesulfonamide (1.0 eq.), the supporting electrolyte (0.6 mmol), the additive ($c_{additive}$), and acetonitrile (15 mL) was stirred at 30 °C for 30 minutes in an undivided 25 mL batch-type glass cell, equipped with two electrodes, arranged with the corresponding inter-electrode gap. Afterwards, the reaction mixture was electrolyzed, applying constant current conditions (current density j, active anodic surface: 5.6 cm^2 for stirring bar SB1 and 4.8 cm^2 for SB2), and a total applied charge of 2.0 *F*. After completed electrolysis, triphenyl phosphate (0.1–1.0 eq.) was added as internal standard for quantification by ^{31}P NMR. The sample for the ^{31}P NMR measurement was prepared by mixing 0.4 mL of the reaction solution with 0.1 mL of CD_3CN.
For reactions under inert conditions, an argon atmosphere and anhydrous acetonitrile were applied, and the cell was flushed with argon for 15 minutes prior to filling it with the reaction mixture.

2.3 General Protocol C: Optimization Experiments in Undivided 5 mL Teflon™ Screening Cells

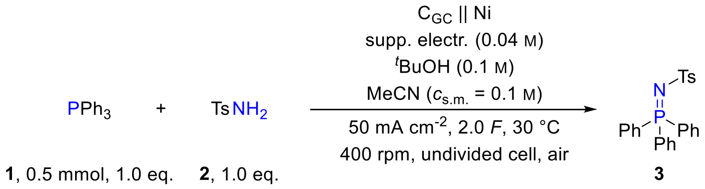

A solution of triphenylphosphine (0.5 mmol, 1.0 eq.), *p*-toluenesulfonamide (0.5 mmol, 1.0 eq.), the supporting electrolyte (0.2 mmol), and *tert*-butanol (0.1 M) in acetonitrile (5 mL) was stirred (stirring speed v_{stirr} = 400 rpm) in a temperature adjusted undivided 5 mL Teflon™ screening cell for 30 minutes until the reaction mixture reached a constant temperature of 30 °C. Then, the solution was electrolyzed under constant current conditions (current density j = 50 mA cm^{-2}, active anodic surface: 1.8 cm^2), employing a glassy carbon anode and a nickel cathode, until a total applied charge of 2.0 *F* was applied. After completed electrolysis, triphenyl phosphate (1.0 eq.) was added as internal standard for quantification by ^{31}P NMR.

2.4 Scale-up of the Reaction: 60 mmol Scale in Undivided 200 mL Batch-Type Glass Cell

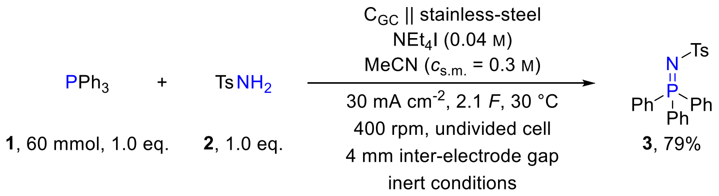

The undivided 200 mL batch-type glass cell, equipped with a glassy carbon anode and a freshly sanded stainless-steel cathode (inter-electrode gap: 4 mm), was flushed with argon for 15 minutes. Then, triphenylphosphine (15.74 g, 60 mmol, 1.0 eq.), *p*-toluenesulfonamide (10.27 g, 60 mmol, 1.0 eq.), NEt_4I (2.06 g, 8 mmol), and anhydrous acetonitrile (200 mL) were added, and the resulting mixture was stirred (stirring speed v_{stirr} = 400 rpm) under argon atmosphere at 30 °C for 30 minutes. Afterwards, the reaction mixture was electrolyzed, applying constant current conditions (current density j = 30 mA cm^{-2}, active anodic surface: 32.4 cm^2) and a total applied charge of 2.1 *F*.
After completed electrolysis, the product was crystallized from the electrolyte at 0 °C. Filtration and washing with acetonitrile yielded the first product fraction (14.72 g) as a colorless solid.
The filtrate was concentrated under reduced pressure to approximately 80 mL, from which further solid precipitated at 0 °C, which was filtered off. Water (50 mL) was added to this solid, followed by extraction with dichloromethane (3 x 50 mL), washing with water (100 mL), drying of the combined organic fractions over anhydrous magnesium sulfate, and removing of the solvent. This yielded the second product fraction (3.24 g) as a colorless solid.
The residual filtrate was concentrated under reduced pressure, water (100 mL) was added, and the mixture was extracted with dichloromethane (3 x 100 mL). The combined organic fractions were dried over anhydrous magnesium sulfate and the solvent was removed under reduced pressure. Crystallization from boiling acetonitrile (approximately 30 mL) yielded the third product fraction (1.31 g).
The remaining filtrate was purified by column chromatography (silica gel, cyclohexane/ ethyl acetate, gradient: 12% to 40% ethyl acetate *v*/*v*), yielding the last product fraction (1.19 g).
The combined aqueous fractions were concentrated under reduced pressure and the residue was dried at 100 °C under vacuum for 5 hours, yielding the recovered NEt_4I (1.810 g, 7 mmol, 88%) as a light brown solid.
Overall, the scale-up experiment yielded 20.46 g of the product (47 mmol, 79%) as a colorless solid within 3.5 hours, with an average electric power of 7.00 W, which corresponds to a specific energy consumption of 1.46 kJ per mmol starting material.

2.5 Electrolysis in Repetitive Mode by Reuse of the Electrolyte and Feeding of Starting Material

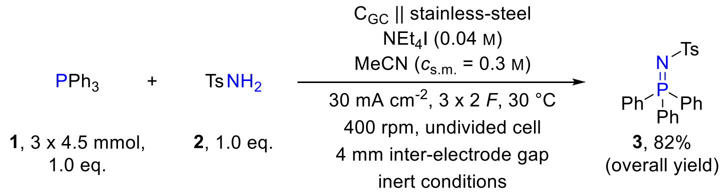

According to general protocol **A** triphenylphosphine (**1**, 1.180 g, 4.5 mmol, 1.0 eq.) was reacted with *p*-toluenesulfonamide (**2**, 0.771 g, 4.5 mmol, 1.0 eq.), using an electrolyte consisting of NEt_4I (0.154 g, 0.6 mmol) in anhydrous acetonitrile (15 mL). After 2 *F* of applied charge, the precipitated product **3** (1.252 g, 2.9 mmol, 64% based on 4.5 mmol substrate) was filtered off under argon atmosphere (followed by separately washing with water) and the resulting filtrate was enriched with further **1** (1.180 g, 4.5 mmol, 1.0 eq.) and **2** (0.771 g, 4.5 mmol, 1.0 eq.). Then, the mixture was stirred for 30 minutes at 30 °C and electrolyzed again, applying further 2 *F* of applied charge. After completed electrolysis, the precipitated product **3** (1.489 g, 3.5 mmol, 77% based on 4.5 mmol substrate) was again filtered off under argon atmosphere (followed by separately washing with water) and the resulting filtrate was enriched with further **1** (1.180 g, 4.5 mmol, 1.0 eq.) and **2** (0.771 g, 4.5 mmol, 1.0 eq.). Due to solvent losses during filtration, further anhydrous acetonitrile (5 mL) was added. The resulting reaction mixture was stirred for 30 minutes at 30 °C and afterwards electrolyzed again, applying additional 2 *F* of applied charge. Then, the precipitated product (1.540 g, 3.6 mmol, 79% based on 4.5 mmol substrate) was filtered off again and washed with acetonitrile. The residual filtrate was analysed by ^{31}P NMR, using triphenyl phosphate (0.655 g, 2.0 mmol) as internal standard. It contained further product (1.1 mmol), triphenylphosphine oxide (**TPPO**, 0.6 mmol) and unconverted **1** (1.0 mmol), resulting in an overall yield of 82% (11.0 mmol) for **3**, 4% (0.6 mmol) for **TPPO** and 7% (1.0 mmol) for unconverted **1**.

3 Optimization Data

The reaction conditions were optimized using triphenylphosphine (**1**) and *p*-toluenesulfonamide (**2**) as test substrates, which were converted into iminophosphorane **3**. Triphenylphosphine oxide (**TPPO**) was formed as by-product. The optimization experiments were conducted according to general protocols **B** and **C**. The yields of product **3**, residual starting material **1** and **TPPO** as by-product were determined by ^{31}P NMR spectroscopy, using triphenyl phosphate (0.1–1.0 eq.) as internal standard, which was added to the reaction mixture after completed electrolysis.

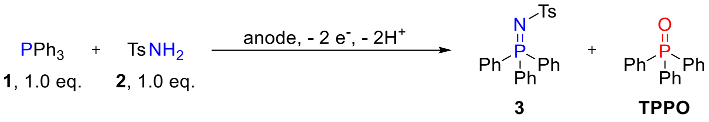

Scheme S1: General strategy for the synthesis of iminophosphorane **3** from triphenylphosphine and *p*-toluenesulfonamide.

3.1 Optimization Experiments in Undivided 5 mL Teflon™ Screening Cells

Table S1: Influence of the supporting electrolyte.

Entry	Supporting electrolyte	**3**[a] (%)	**TPPO**[a] (%)	**1**[a,b] (%)
1	NBu_4PF_6	traces	38	33
2	NEt_4Cl	2	91	0
3	NEt_4Br	1	91	0
4[c]	NEt_4I	14	82	0

Triphenylphosphine (**1**, 0.5 mmol, 1.0 eq.), *p*-toluenesulfonamide (**2**, 0.5 mmol, 1.0 eq.), supporting electrolyte (0.04 M), *t*BuOH (0.1 M) in MeCN (5 mL), anode: glassy carbon, cathode: nickel, current density: 50 mA cm^{-2} (active anodic surface: 1.8 cm^2), applied charge: 2.0 *F*, 30 °C, stirring speed: 400 rpm, air, undivided 5 mL Teflon™ cell. [a] Yields determined by ^{31}P NMR spectroscopy, using triphenyl phosphate (1.0 eq.) as internal standard. [b] Residual **1** due to uncomplete conversion. [c] Isolated yields: **3** (12%), **TPPO** (73%).

3.2 Optimization Experiments in Undivided 25 mL Beaker-Type Glass Cells

To test the reaction under inert conditions, the reaction set-up was changed to undivided 25 mL beaker-type glass cells, since they possess an outlet through which an argon stream can be applied.

Table S2: Influence of oxygen and water.

Entry	Conditions	**3**[a] (%)	**TPPO**[a] (%)	**1**[a,b] (%)
1[c]	non-inert	33	59	6
2[d]	inert	50	21	12
3[e]	inert + H_2O (1.0 eq.)	29	71	0

Triphenylphosphine (**1**, 1.5 mmol, 1.0 eq.), *p*-toluenesulfonamide (**2**, 1.5 mmol, 1.0 eq.), NEt_4I (0.04 M), *t*BuOH (0.1 M) in MeCN (15 mL), anode: glassy carbon, cathode: nickel, inter-electrode gap: 9 mm, current density: 50 mA cm^{-2} (stirring bar SB1, active anodic surface: 5.6 cm^2), applied charge: 2.0 *F*, 30 °C, stirring speed: 400 rpm, undivided 25 mL beaker-type glass cell. Non-inert conditions: The reaction was conducted under air. Inert conditions: The reaction was conducted under argon atmosphere, using anhydrous MeCN. [a] Yields determined by ^{31}P NMR spectroscopy, using triphenyl phosphate (0.1–1.0 eq.) as internal standard. [b] Residual **1** due to uncomplete conversion. [c] Yields of the replicate experiment: **3** (31%), **TPPO** (61%), **1** (0%). [d] Yields of the replicate experiments: Exp. 1: **3** (55%), **TPPO** (23%), **1** (11%); Exp. 2: **3** (56%), **TPPO** (21%), **1** (13%); Exp. 3: **3** (52%), **TPPO** (20%), **1** (10%); Exp. 4 (with stirring bar SB2, active anodic surface: 4.8 cm^2): **3** (50%), **TPPO** (28%), **1** (9%). [e] Yields of the replicate experiments: Exp. 1: **3** (31%), **TPPO** (69%), **1** (0%); Exp. 2 (with stirring bar SB2, active anodic surface: 4.8 cm^2): **3** (17%), **TPPO** (82%), **1** (0%).

Table S3: Influence of the supporting electrolyte and possible redox mediators.

Entry	Conditions	**3**[a] (%)	**TPPO**[a] (%)	**1**[a,b] (%)
1	NBu_4PF_6	3	12	67
2	NEt_4Cl	49	30	10
3	NEt_4Br	48	24	12
4[c]	NEt_4I	50	21	12
5[d]	PhI + NMe_4OAc	3	96	0
6[e]	*N*(*p*-Br-Ph)$_3$ + NBu_4PF_6	14	12	57

Triphenylphosphine (**1**, 1.5 mmol, 1.0 eq.), *p*-toluenesulfonamide (**2**, 1.5 mmol, 1.0 eq.), supporting electrolyte (0.04 M), ^{t}BuOH (0.1 M) in MeCN (15 mL), anode: glassy carbon, cathode: nickel, inter-electrode gap: 9 mm, current density: 50 mA cm^{-2} (stirring bar SB1, active anodic surface: 5.6 cm^2), applied charge: 2.0 *F*, 30 °C, stirring speed: 400 rpm, undivided 25 mL beaker-type glass cell, inert conditions. [a] Yields determined by ^{31}P NMR spectroscopy, using triphenyl phosphate (0.1–1.0 eq.) as internal standard. [b] Residual **1** due to uncomplete conversion. [c] Yields of the replicate experiments: Exp. 1: **3** (55%), **TPPO** (23%), **1** (11%); Exp. 2: **3** (56%), **TPPO** (21%), **1** (13%); Exp. 3: **3** (52%), **TPPO** (20%), **1** (10%); Exp. 4 (with stirring bar SB2, active anodic surface: 4.8 cm^2): **3** (50%), **TPPO** (28%), **1** (9%). [d] PhI (0.04 M), NMe_4OAc (0.08 M), current density: 5 mA cm^{-2}. [e] *N*(*p*-Br-Ph)$_3$ = tris(4-bromophenyl)amine (0.04 M), NBu_4PF_6 (0.04 M).

Table S4: Influence of the type of stirring bar.

Entry	Stirring bar	**3**[a] (%)	**TPPO**[a] (%)	**1**[a,b] (%)
1[c]	SB1	50	21	12
2	SB2	50	28	9

Pictures of the tested stirring bars are provided within the chapter “General Information”. Triphenylphosphine (**1**, 1.5 mmol, 1.0 eq.), *p*-toluenesulfonamide (**1**, 1.5 mmol, 1.0 eq.), NEt_4I (0.04 M), ^{t}BuOH (0.1 M) in MeCN (15 mL), anode: glassy carbon, cathode: nickel, inter-electrode gap: 9 mm, current density: 50 mA cm^{-2} (active anodic surface for stirring bar SB1: 5.6 cm^2; active anodic surface for stirring bar SB2: 4.8 cm^2), applied charge: 2.0 *F*, 30 °C, stirring speed: 400 rpm, undivided 25 mL beaker-type glass cell, inert conditions. [a] Yields determined by ^{31}P NMR spectroscopy, using triphenyl phosphate (0.1–1.0 eq.) as internal standard. [b] Residual **1** due to uncomplete conversion. [c] Yields of the replicate experiments: Exp. 1: **3** (55%), **TPPO** (23%), **1** (11%); Exp. 2: **3** (56%), **TPPO** (21%), **1** (13%); Exp. 3: **3** (52%), **TPPO** (20%), **1** (10%).

The type of stirring bar doesn’t seem to have a significant influence on the reaction. Since stirring bar SB2 was more practicable regarding stirring performance, it was further used.

Table S5: Influence of the additive.

Entry	Additive	Additive concentration, $c_{additive}$ (M)	**3**[a] (%)	**TPPO**[a] (%)	**1**[a,b] (%)
1[c]	-	-	42	19	8
2[d]	^{t}BuOH	0.10	50	28	9
3[e]	MeOH	0.10	49	29	10
4[f,g]	MeOH	0.25	79	18	3
5	MeOH	0.40	80	20	0
6	EtOH	0.10	43	19	19
7	EtOH	0.25	50	18	20
8[h]	HFIP	0.10	73	18	0
9[i]	HFIP	0.15	91	5	1
10[f,j]	HFIP	0.25	91	9	0
11[k]	TFE	0.10	81	10	6
12	TFE	0.15	90	7	1
13[l]	TFE	0.25	90	9	0
14	AcOH	0.10	1	98	0
15[f]	TFA	0.10	0	100	0
16	pyridine	0.10	44	12	24

Triphenylphosphine (**1**, 1.5 mmol, 1.0 eq.), *p*-toluenesulfonamide (**2**, 1.5 mmol, 1.0 eq.), NEt_4I (0.04 M), additive in MeCN (15 mL), anode: glassy carbon, cathode: nickel, inter-electrode gap: 9 mm, current density: 50 mA cm^{-2} (stirring bar SB2, active anodic surface: 4.8 cm^2), applied charge: 2.0 *F*, 30 °C, stirring speed: 400 rpm, undivided 25 mL beaker-type glass cell, inert conditions. HFIP = 1,1,1,3,3,3-hexafluoro-2-propanol. TFE = 2,2,2-trifluoroethanol. TFA = trifluoroacetic acid. [a] Yields determined by ^{31}P NMR spectroscopy, using triphenyl phosphate (0.1–1.0 eq.) as internal standard. [b] Residual **1** due to uncomplete conversion. [c] Yields of the replicate experiments: Exp. 1: **3** (40%), **TPPO** (22%), **1** (11%); Exp. 2 (with stirring bar SB1, active anodic surface: 5.6 cm^2): **3** (48%), **TPPO** (26%), **1** (13%). [d] Yields of the replicate experiments (with stirring bar SB1, active anodic surface: 5.6 cm^2): Exp. 1: **3** (50%), **TPPO** (21%), **1** (12%); Exp. 2: **3** (55%), **TPPO** (23%), **1** (11%); Exp. 3: **3** (56%), **TPPO** (21%), **1** (13%); Exp. 4: **3** (52%), **TPPO** (20%), **1** (10%). [e] Yields of the replicate experiment (with stirring bar SB1, active anodic surface: 5.6 cm^2): **3** (55%), **TPPO** (25%), **1** (9%). [f] With stirring bar SB1, active anodic surface: 5.6 cm^2. [g] Yields of the replicate experiment: **3** (80%), **TPPO** (16%), **1** (3%). [h] Yields of the replicate experiments: Exp. 1: **3** (71%), **TPPO** (17%), **1** (2%); Exp. 2 (with stirring bar SB1, active anodic surface: 5.6 cm^2): **3** (83%), **TPPO** (13%), **1** (3%). [i] Yields of the replicate experiments: Exp. 1: **3** (94%), **TPPO** (5%), **1** (0%); Exp. 2: **3** (85%), **TPPO** (10%), **1** (0%); Exp. 3: **3** (75%), **TPPO** (25%), **1** (0%). [j] Yields of the replicate experiments (with stirring bar SB2, active anodic surface: 4.8 cm^2): Exp. 1: **3** (96%), **TPPO** (3%), **1** (0%); Exp. 2: **3** (81%), **TPPO** (19%), **1** (0%). [k] Yields of the replicate experiments: Exp. 1: **3** (85%), **TPPO** (9%), **1** (6%); Exp. 2 (with stirring bar SB1, active anodic surface: 5.6 cm^2): **3** (85%), **TPPO** (7%), **1** (8%). [l] Yields of the replicate experiment: **3** (90%), **TPPO** (10%), **1** (0%).

In the screening of additives, the best yields for **3** could be obtained with HFIP and TFE, which performed similarly. In both cases yields of **3** increased significantly when increasing the additive concentration from 0.1 M to 0.15 M. At higher concentrations no significant change of yield could be observed. Due to our extensive experience with HFIP as a unique solvent,[5–9] it was chosen as the additive for subsequent optimization experiments.

Table S6: Influence of the cation of the supporting electrolyte.

Entry	Supporting Electrolyte	**3**[a] (%)	**TPPO**[a] (%)	**1**[a,b] (%)
1[c]	NMe_4I	62	10	0
2[d]	NEt_4I	91	5	1
3	NBu_4I	70	22	0

Triphenylphosphine (**1**, 1.5 mmol, 1.0 eq.), *p*-toluenesulfonamide (**2**, 1.5 mmol, 1.0 eq.), supporting electrolyte (0.04 M), 1,1,1,3,3,3-hexafluoro-2-propanol (HFIP, 0.15 M) in MeCN (15 mL), anode: glassy carbon, cathode: nickel, inter-electrode gap: 9 mm, current density: 50 mA cm^{-2} (stirring bar SB2, active anodic surface: 4.8 cm^2), applied charge: 2.0 *F*, 30 °C, stirring speed: 400 rpm, undivided 25 mL beaker-type glass cell, inert conditions. [a] Yields determined by ^{31}P NMR spectroscopy, using triphenyl phosphate (1.0 eq.) as internal standard. [b] Residual **1** due to uncomplete conversion. [c] NMe_4I could not be fully dissolved, and the reaction mixture was electrolyzed as a suspension. [d] Yields of the replicate experiments: Exp. 1: **3** (94%), **TPPO** (5%), **1** (0%); Exp. 2: **3** (85%), **TPPO** (10%), **1** (0%); Exp. 3: **3** (75%), **TPPO** (25%), **1** (0%).

Table S7: Influence of the cathode material.

Entry	Cathode material	**3**[a] (%)	**TPPO**[a] (%)	**1**[a,b] (%)
1[c]	nickel	91	5	1
2	platinum	88	10	0
3	isostatic graphite	3	61	4
4[d]	stainless-steel	87	8	1

Triphenylphosphine (**1**, 1.5 mmol, 1.0 eq.), *p*-toluenesulfonamide (**2**, 1.5 mmol, 1.0 eq.), NEt_4I (0.04 M), 1,1,1,3,3,3-hexafluoro-2-propanol (HFIP, 0.15 M) in MeCN (15 mL), anode: glassy carbon, cathode, inter-electrode gap: 9 mm, current density: 50 mA cm^{-2} (stirring bar SB2, active anodic surface: 4.8 cm^2), applied charge: 2.0 *F*, 30 °C, stirring speed: 400 rpm, undivided 25 mL beaker-type glass cell, inert conditions. [a] Yields determined by ^{31}P NMR spectroscopy, using triphenyl phosphate (1.0 eq.) as internal standard. [b] Residual **1** due to uncomplete conversion. [c] Yields of the replicate experiments: Exp. 1: **3** (94%), **TPPO** (5%), **1** (0%); Exp. 2: **3** (85%), **TPPO** (10%), **1** (0%); Exp. 3: **3** (75%), **TPPO** (25%), **1** (0%). [d] Yields of the replicate experiments: Exp. 1: **3** (90%), **TPPO** (6%), **1** (1%); Exp. 2: **3** (83%), **TPPO** (12%), **1** (2%).

The screening of cathode materials revealed that nickel, platinum and stainless-steel yielded the product in comparable amounts. Since stainless-steel is superior to nickel and platinum regarding availability, costs, and safety, it was further used as the cathode material.

Table S8: Influence of the anode material.

Entry	Anode material	**3**[a] (%)	**TPPO**[a] (%)	**1**[a,b] (%)
1[c]	glassy carbon	87	8	1
2[d]	isostatic graphite	78	19	0

Triphenylphosphine (**1**, 1.5 mmol, 1.0 eq.), *p*-toluenesulfonamide (**2**, 1.5 mmol, 1.0 eq.), NEt_4I (0.04 M), 1,1,1,3,3,3-hexafluoro-2-propanol (HFIP, 0.15 M) in MeCN (15 mL), anode, cathode: stainless-steel, inter-electrode gap: 9 mm, current density: 50 mA cm^{-2} (stirring bar SB2, active anodic surface: 4.8 cm^2), applied charge: 2.0 *F*, 30 °C, stirring speed: 400 rpm, undivided 25 mL beaker-type glass cell, inert conditions. [a] Yields determined by ^{31}P NMR spectroscopy, using triphenyl phosphate (1.0 eq.) as internal standard. [b] Residual **1** due to uncomplete conversion. [c] Yields of the replicate experiments: Exp. 1: **3** (90%), **TPPO** (6%), **1** (1%); Exp. 2: **3** (83%), **TPPO** (12%), **1** (2%). [d] Yields of the replicate experiment: **3** (70%), **TPPO** (27%), **1** (0%).

Table S9: Influence of the inter-electrode gap and stirring speed.

Entry	Inter-electrode gap	Stirring speed	$ØP_{el}$[a] (W)	$W_{el}/n_{s.m.}$[b] (kJ $mmol^{-1}$)	**3**[c] (%)	**TPPO**[c] (%)	**1**[c,d] (%)
1[e]	9 mm	400 rpm	2.65	2.12	87	8	1
2[f]	4 mm	400 rpm	1.54	1.23	90	7	1
3	4 mm	600 rpm	1.53	1.22	92	6	1

Triphenylphosphine (**1**, 1.5 mmol, 1.0 eq.), *p*-toluenesulfonamide (**2**, 1.5 mmol, 1.0 eq.), NEt_4I (0.04 M), 1,1,1,3,3,3-hexafluoro-2-propanol (HFIP, 0.15 M) in MeCN (15 mL), anode: glassy carbon, cathode: stainless-steel, inter-electrode gap, current density: 50 mA cm^{-2} (stirring bar SB2, active anodic surface: 4.8 cm^2), applied charge: 2.0 *F*, 30 °C, stirring speed, undivided 25 mL beaker-type glass cell, inert conditions. [a] Average electric power, calculated from the during the reaction measured electric current and cell voltage. [b] Specific energy consumption (per mmol of starting material), calculated from the during the reaction measured electric current and cell voltage. [c] Yields determined by ^{31}P NMR spectroscopy, using triphenyl phosphate (1.0 eq.) as internal standard. [d] Residual **1** due to uncomplete conversion. [e] Yields of the replicate experiments: Exp. 1: **3** (90%), **TPPO** (6%), **1** (1%), 2.61 W, 2.09 kJ $mmol^{-1}$; Exp. 2: **3** (83%), **TPPO** (12%), **1** (2%), 2.84 W, 2.27 kJ $mmol^{-1}$. [f] Yields of the replicate experiment: **3** (87%), **TPPO** (8%), **1** (2%), 1.34 W, 1.07 kJ $mmol^{-1}$.

Different inter-electrode gaps have been tested to increase the energy efficiency of the reaction by lowering the cell voltage. With a smaller inter-electrode gap of 4 mm the specific energy consumption of the reaction could be lowered by 42%, with product yields remaining constant. A higher stirring speed had no influence on the yield and the energy consumption. Therefore, further experiments were performed with an inter-electrode gap of 4 mm and a stirring speed of 400 rpm.

Table S10: Influence of the current density.

Entry	Current density (mA cm^{-2})	$ØP_{el}$[a] (W)	$W_{el}/n_{s.m.}$[b] (kJ $mmol^{-1}$)	**3**[c] (%)	**TPPO**[c] (%)	**1**[c,d] (%)
1[e]	50	1.54	1.23	90	7	1
2	40	1.02	1.02	92	6	1
3	30	0.65	0.87	91	6	0
4	20	0.36	0.73	89	8	0
5	10	0.14	0.57	88	9	1

Triphenylphosphine (**1**, 1.5 mmol, 1.0 eq.), *p*-toluenesulfonamide (**1**, 1.5 mmol, 1.0 eq.), NEt_4I (0.04 M), 1,1,1,3,3,3-hexafluoro-2-propanol (HFIP, 0.15 M) in MeCN (15 mL), anode: glassy carbon, cathode: stainless-steel, inter-electrode gap: 4 mm, current density (stirring bar SB2, active anodic surface: 4.8 cm^2), applied charge: 2.0 *F*, 30 °C, stirring speed: 400 rpm, undivided 25 mL beaker-type glass cell, inert conditions. [a] Average electric power, calculated from the during the reaction measured electric current and cell voltage. [b] Specific energy consumption (per mmol of starting material), calculated from the during the reaction measured electric current and cell voltage. [c] Yields determined by ^{31}P NMR spectroscopy, using triphenyl phosphate (1.0 eq.) as internal standard. [d] Residual **1** due to uncomplete conversion. [e] Yields of the replicate experiment: **3** (87%), **TPPO** (8%), **1** (2%), 1.34 W, 1.07 kJ $mmol^{-1}$.

The current density in a range between 10–50 mA cm^{-2} had no significant impact on the product yield. However, the energy efficiency of the reaction could be increased by lowering the current density. To ensure a high space-time yield in addition to a low energy consumption, a current density of 30 mA cm^{-2} was chosen as the appropriate value. With this, the specific energy consumption of the reaction could be lowered by 29% compared to a current density of 50 mA cm^{-2}.

Table S11: Influence of the starting material concentration.

Entry	Starting material concentration	$ØP_{el}$[a] (W)	$W_{el}/n_{s.m.}$[b] (kJ $mmol^{-1}$)	**3**[c] (%)	**TPPO**[c] (%)	**1**[c,d] (%)
1	0.1 M (1.5 mmol PPh_3)	0.65	0.87	91	6	0
2	0.3 M (4.5 mmol PPh_3)	0.68	0.91	95	4	0

Triphenylphosphine (**1**, 1.0 eq.), *p*-toluenesulfonamide (**2**, 1.0 eq.), NEt_4I (0.04 M), 1,1,1,3,3,3-hexafluoro-2-propanol (HFIP, 0.15 M) in MeCN (15 mL), anode: glassy carbon, cathode: stainless-steel, inter-electrode gap: 4 mm, current density: 30 mA cm^{-2} (stirring bar SB2, active anodic surface: 4.8 cm^2), applied charge: 2.0 *F*, 30 °C, stirring speed: 400 rpm, undivided 25 mL beaker-type glass cell, inert conditions. [a] Average electric power, calculated from the during the reaction measured electric current and cell voltage. [b] Specific energy consumption (per mmol of starting material), calculated from the during the reaction measured electric current and cell voltage. [c] Yields determined by ^{31}P NMR spectroscopy, using triphenyl phosphate (1.0 eq.) as internal standard. [d] Residual **1** due to uncomplete conversion.

A higher starting material concentration resulted in comparable high yields. Furthermore, a precipitation of the product during the electrolysis could be observed.

Table S12: Influence of the HFIP concentration.

Entry	HFIP concentration (M)	$ØP_{el}$[a] (W)	$W_{el}/n_{s.m.}$[b] (kJ $mmol^{-1}$)	**3**[c] (%)	**TPPO**[c] (%)	**1**[c,d] (%)
1[e]	no HFIP	0.68	0.92	89	5	1
2	0.025	0.68	0.91	92	4	2
3	0.05	0.72	0.96	93	4	2
4	0.10	0.68	0.91	94	4	1
5	0.15	0.68	0.91	95	4	0
6	0.20	0.68	0.91	94	4	0
7	0.45	0.67	0.90	93	5	1

Triphenylphosphine (**1**, 4.5 mmol, 1.0 eq.), *p*-toluenesulfonamide (**2**, 4.5 mmol, 1.0 eq.), NEt_4I (0.04 M), 1,1,1,3,3,3-hexafluoro-2-propanol (HFIP) in MeCN (15 mL), anode: glassy carbon, cathode: stainless-steel, inter-electrode gap: 4 mm, current density: 30 mA cm^{-2} (stirring bar SB2, active anodic surface: 4.8 cm^2), applied charge: 2.0 *F*, 30 °C, stirring speed: 400 rpm, undivided 25 mL beaker-type glass cell, inert conditions. [a] Average electric power, calculated from the during the reaction measured electric current and cell voltage. [b] Specific energy consumption (per mmol of starting material), calculated from the during the reaction measured electric current and cell voltage. [c] Yields determined by ^{31}P NMR spectroscopy, using triphenyl phosphate (1.0 eq.) as internal standard. [d] Residual **1** due to uncomplete conversion. [e] Yields of the replicate experiments: Exp. 1: **3** (88%), **TPPO** (6%), **1** (4%), 0.70 W, 0.93 kJ $mmol^{-1}$; Exp. 2: **3** (88%), **TPPO** (6%), **1** (3%), 0.73 W, 0.98 kJ $mmol^{-1}$.

The highest yield was obtained with 0.15 M HFIP as an additive (Table S12, entry 5). However, HFIP turned out to have no significant influence on the product yield under these conditions, yielding **3** in 89% in absence of HFIP (Table S12, entry 1). In a control experiment, methanol (0.15 M) instead of HFIP was tested as an additive under these conditions, which led to a lower product yield (**3**: 82%, **TPPO**: 14%, unconverted **1**: 1%, determined by ^{31}P NMR). Due to sustainability reasons the reaction conditions without HFIP were chosen to be the appropriate ones.

4 Cyclic Voltammetry Studies and Mechanistic Considerations

Cyclic Voltammetry Studies:

Cyclic voltammetry (CV) measurements were performed with solutions of the corresponding test substances (5 mM) in 5 mL electrolyte (0.1 M NEt_4PF_6 in MeCN), using a glassy carbon WE, a glassy carbon CE, and an Ag/AgCl RE. Before measuring a sample, the WE was polished with alumina paste (0.5 µm) and the test solution was deoxygenated by bubbling with argon for 5 minutes. For each sample 3 scans with a scan rate of 100 mV s^{-1} were recorded. The potential was referenced versus ferrocene. Figure S3 displays the cyclic voltammograms of the blank electrolyte, of NEt_4I as redox-active supporting electrolyte, of iminophosphorane **3** and of various phosphines and sulfonamides. Potentials of the oxidation peaks (E_p) were determined graphically and are marked in the cyclic voltammograms. Half peak potentials could not be clearly identified and are therefore not provided.

a) **Blank:** NEt_4PF_6 + MeCN

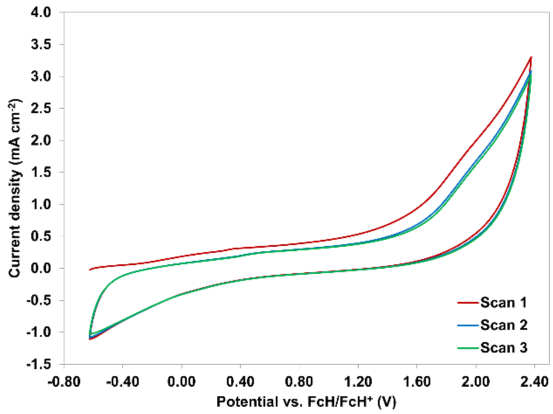

b) **NEt_4I** + NEt_4PF_6 + MeCN

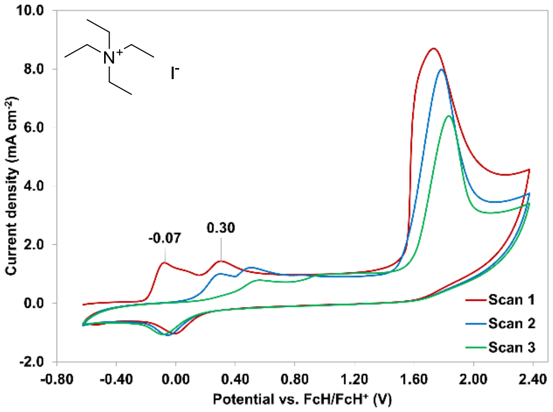

c) **PPh_3** + NEt_4PF_6 + MeCN

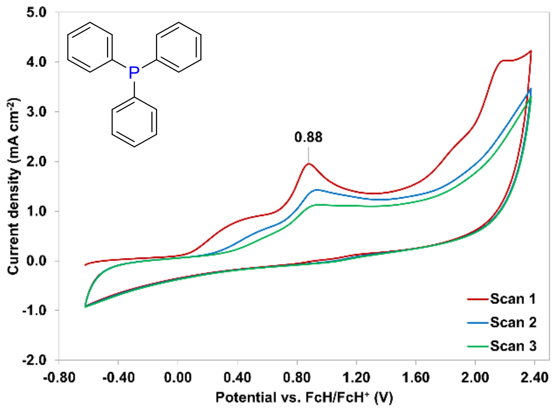

d) **$TsNH_2$** + NEt_4PF_6 + MeCN

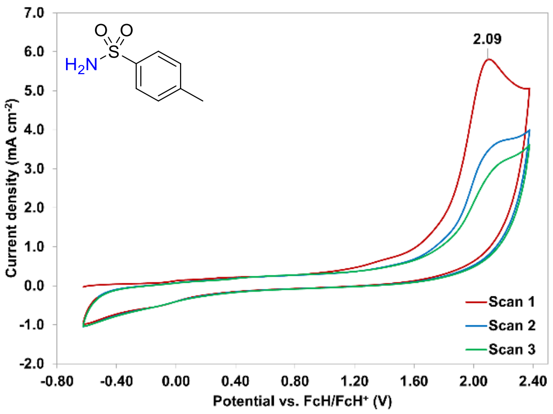

e) **Iminophosphorane 3** + NEt_4PF_6 + MeCN

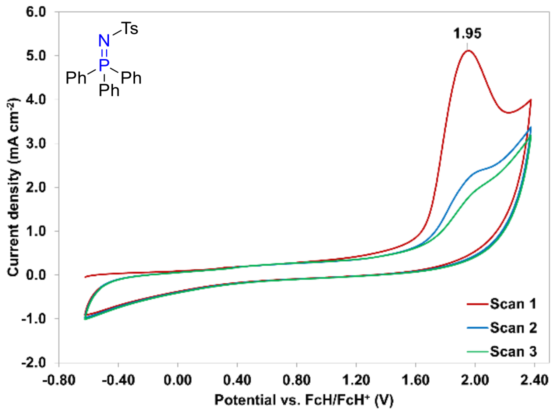

f) **Tris(4-fluorophenyl)phosphine** + NEt_4PF_6 + MeCN

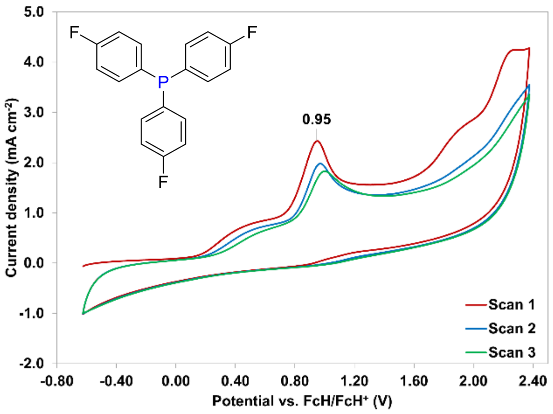

g) **Tri(2-furyl)phosphine** + NEt_4PF_6 + MeCN

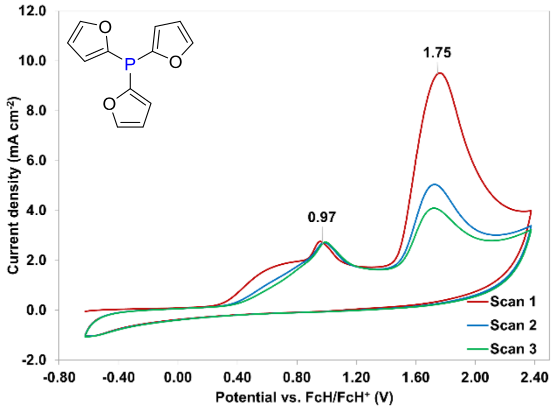

h) **Tricyclohexylphosphine** + NEt_4PF_6 + MeCN

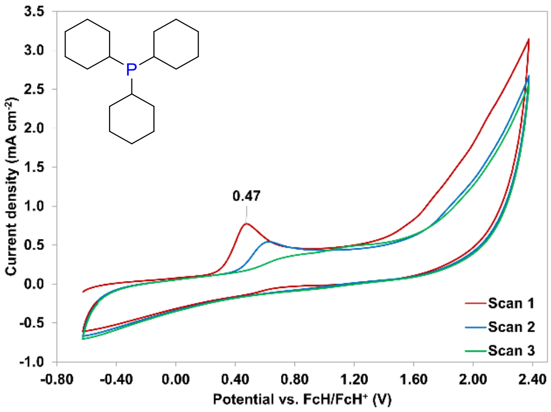

i) **Tris(cyanoethyl)phosphine** + NEt_4PF_6 + MeCN

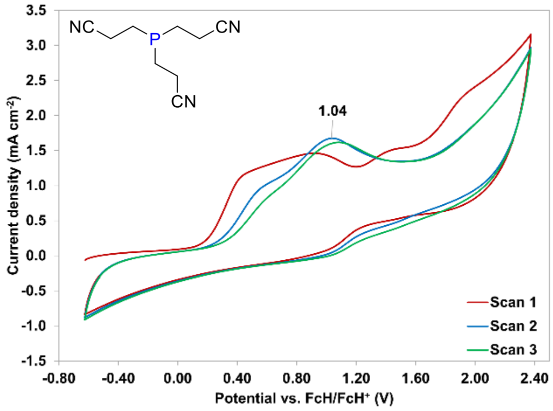

j) **4-Methoxybenzenesulfonamide** + NEt_4PF_6 + MeCN

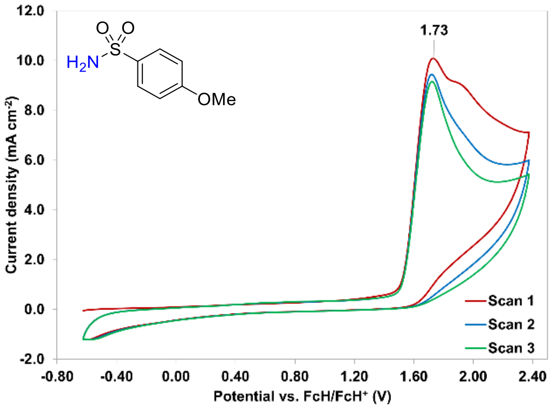

k) **4-Chlorobenzenesulfonamide** + NEt_4PF_6 + MeCN

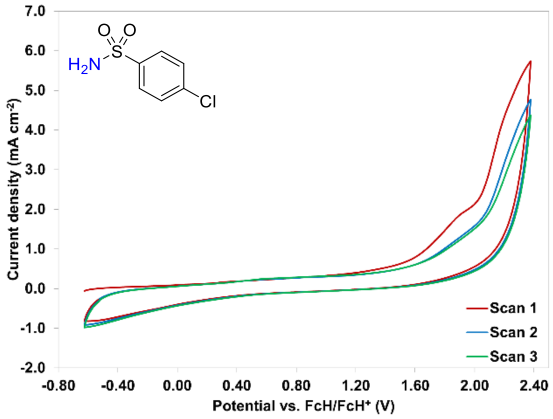

l) **Sulfanilamide** + NEt_4PF_6 + MeCN

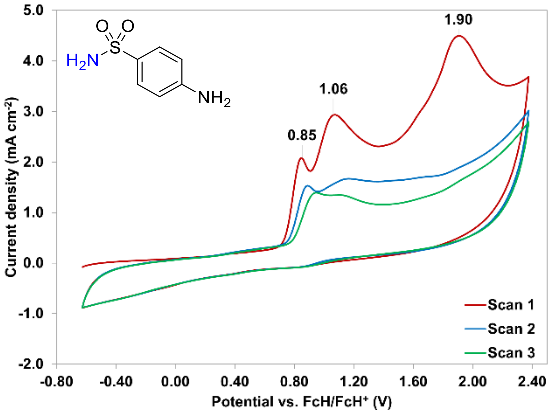

m) **4-Nitrobenzenesulfonamide** + NEt_4PF_6 + MeCN

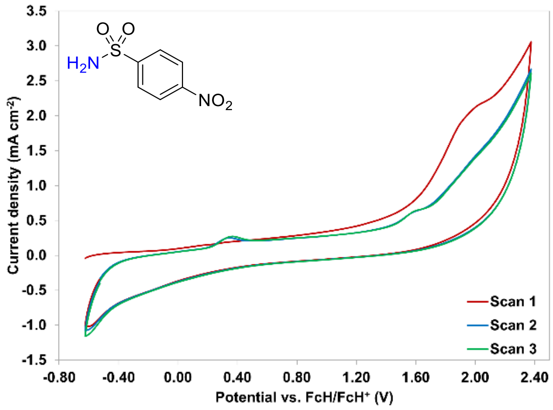

n) **Trifluoromethanesulfonamide** + NEt_4PF_6 + MeCN

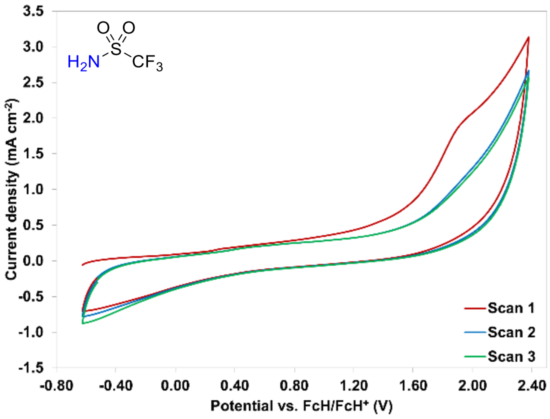

o) **(1*S*)-10-Camphorsulfonamide** + NEt_4PF_6 + MeCN

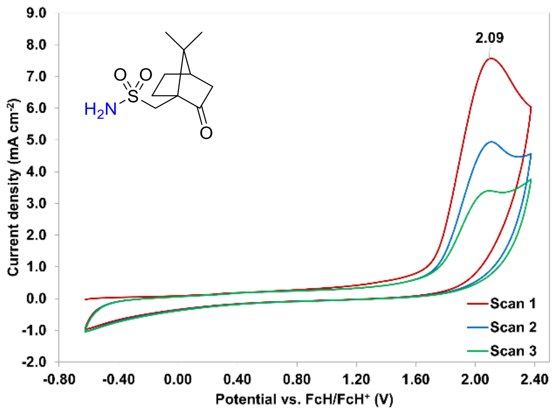

Figure S3: Cyclic voltammograms of selected compounds. Test substance (5 mM) in 5 mL of NEt_4PF_6 (0.1 M) in MeCN. Working electrode: glassy carbon, counter electrode: glassy carbon, reference electrode: Ag/AgCl. Scan rate: 100 V s^{-1}, 3 scans. Potential referenced vs. FcH/FcH^+ (FcH = Fe(η^5-C_5H_5)$_2$).

Control Experiment with Iodine as Oxidizing Agent:

A control experiment was performed, in which elemental iodine (1.0 eq.) was employed as the oxidizing agent instead of using electric current (Scheme S2).

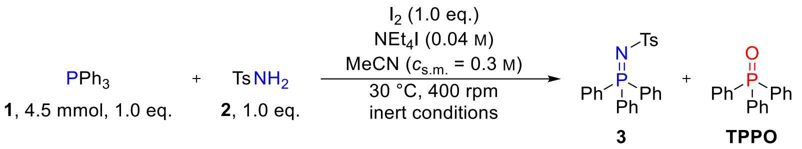

Scheme S2: Reaction of PPh_3 and $TsNH_2$ to iminophosphorane **3**, using elemental iodine as oxidizing agent.

An undivided 25 mL batch-type glass cell, equipped with a sealed PTFE stopper, was flushed with argon for 15 minutes. Then, triphenylphosphine (**1**, 1.180 g, 4.5 mmol, 1.0 eq.), *p*-toluenesulfonamide (**2**, 0.771 g, 4.5 mmol, 1.0 eq.), tetraethylammonium iodide (NEt_4I, 0.154 g, 0.6 mmol), and anhydrous acetonitrile (15 mL) were added, and the resulting solution was stirred (stirring bar SB2, stirring speed v_{stirr} = 400 rpm) under argon atmosphere at 30 °C for 30 minutes. Afterwards, iodine (1.142 g, 4.5 mmol, 1.0 eq.) was added and stirring was continued for 2 hours, until no further conversion was observed by TLC. Then, triphenyl phosphate was added as internal standard and yields were determined by ^{31}P NMR spectroscopy. Iminophosphorane **3** was formed in 79% and **TPPO** in 10%, while observing 9% of unconverted **1**. The obtained yield for **3** is 10% lower compared to the analogous electrochemical experiment (compare Table S12, entry 1).

Mechanistic Conclusions:

According to the cyclic voltammograms in Figure S3, iodide has a significantly (> 0.5 V) lower oxidation potential than the investigated phosphines and sulfonamides. Therefore, iodide is oxidized to I_3^- and I_2 first during the electrolysis reaction. This is supported by the observation of a yellow species forming near the anode surface, when applying an electric current. Furthermore, the same reactivity has been observed using elemental iodine as oxidizing agent instead of electric current (control experiment, see Scheme S2), indicating that the electrochemically generated I_2 equivalent acts as the actual oxidizing agent.

Due to the higher nucleophilicity of phosphines compared to sulfonamides, the corresponding phosphine reacts with the generated I_2 equivalent to form a R_3PI_2 intermediate, which subsequently reacts with the sulfonamide. Elimination of iodide and deprotonation leads to the corresponding iminophosphorane.

5 Limitations

The following phosphines could not be converted into the corresponding iminophosphoranes when being reacted with $TsNH_2$.

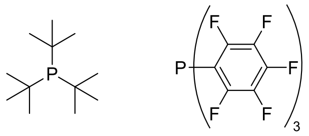

Figure S4: Unsuccessful substrates.

The electrolysis experiments with both phosphines from Figure S4 were carried out under standard conditions, employing the following amounts of substrates:

- tri-*tert*-butylphosphine (3.1 mmol, 1.0 eq.), $TsNH_2$ (3.1 mmol, 1.0 eq.)
- tris(pentafluorophenyl)phosphine (3.0 mmol, 1.0 eq.), $TsNH_2$ (3.0 mmol, 1.0 eq.).

6 Compound Characterization

6.1 Starting Materials

All starting materials were obtained from common commercial sources and were employed without additional purification, except for 2,4,6-triisopropylbenzenesulfonamide (**24**) and (1*S*)-10-camphorsulfonamide (**25**), which were synthesized from the corresponding sulfonyl chlorides.

2,4,6-Triisopropylbenzenesulfonamide (24)

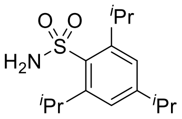

According to a known protocol,[10] a solution of 2,4,6-triisopropylbenzenesulfonyl chloride (4.543 g, 15.0 mmol, 1.0 eq.) in chloroform (23 mL) was treated with an aqueous solution of ammonia (25%, 5.6 mL, 75.0 mmol, 5.0 eq.), and stirred at room temperature for 7 hours. Then, water (50 mL) was added, and the mixture was extracted with chloroform (2 x 50 mL). The aqueous layer was treated with caustic soda (1 M, 30 mL) and extracted again with chloroform (50 mL). The combined organic fractions were washed with water (100 mL), dried over anhydrous magnesium sulfate and the solvent was removed under reduced pressure, yielding **24** (3.823 g, 13.5 mmol, 90%) as a colorless solid, which was used without further purification.

^{1}H NMR (400 MHz, $CDCl_3$): δ = 7.16 (s, 2H), 4.92 (s, 2H), 4.11 (hept, *J* = 6.7 Hz, 2H), 2.90 (hept, *J* = 6.9 Hz, 1H), 1.28 (d, *J* = 6.7 Hz, 12H), 1.25 (d, *J* = 6.9 Hz, 6H) ppm.

^{13}C NMR (101 MHz, $CDCl_3$): δ = 152.6, 149.2, 135.1, 123.7, 34.2, 29.8, 24.8, 23.7 ppm.

MS for $C_{15}H_{25}NO_2S$ (ESI+, HPLC-MS) $[M+H]^+$ m/z: calc: 284, found: 284.

The analytical data are in accordance with the literature.[10]

(1*S*)-10-Camphorsulfonamide (25)

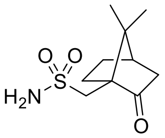

According to a known protocol,[11] a solution of (1*S*)-10-camphorsulfonyl chloride (3.763 g, 15.0 mmol, 1.0 eq.) in dichloromethane (35 mL) was treated with an aqueous solution of ammonia (25%, 33 mL, 466.3 mmol, 31.0 eq.) at 0 °C, and stirred for further 1 hour. Then, the mixture was extracted with dichloromethane (3 x 30 mL). The combined organic fractions were washed with water (100 mL), dried over anhydrous magnesium sulfate and the solvent was removed under reduced pressure, yielding **25** (2.928 g, 12.7 mmol, 84%) as a colorless solid, which was used without further purification.

^{1}H NMR (400 MHz, $CDCl_3$): δ = 5.49 (s, 2H), 3.50 (d, *J* = 15.0 Hz, 1H), 3.12 (d, *J* = 15.0 Hz, 1H), 2.46 – 2.35 (m, 1H), 2.22 – 2.10 (m, 2H), 2.09 – 1.89 (m, 3H), 1.50 – 1.40 (m, 1H), 1.00 (s, 3H), 0.91 (s, 3H) ppm.

^{13}C NMR (101 MHz, $CDCl_3$): δ = 217.7, 59.4, 53.9, 49.2, 43.1, 42.9, 27.1, 26.8, 20.0, 19.5 ppm.

MS for $C_{10}H_{17}NO_3S$ (ESI+, HPLC-MS) $[M+H]^+$ m/z: calc: 232, found: 232.

The analytical data are in accordance with the literature.[11,12]

6.2 *N*-Sulfonyl iminophosphoranes

4-Methyl-*N*-(triphenyl-λ^5-phosphanylidene)benzenesulfonamide (3)

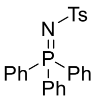

According to general protocol **A**, a solution of triphenylphosphine (1.180 g, 4.5 mmol, 1.0 eq.), *p*-toluenesulfonamide (0.771 g, 4.5 mmol, 1.0 eq.), and NEt_4I (0.154 g, 0.6 mmol) in anhydrous MeCN (15 mL) was electrolyzed in an undivided 25 mL batch-type glass cell under inert conditions, applying 2.0 *F* of electric charge. The product precipitated from the electrolyte, was filtered off, and washed with acetonitrile, yielding pure **3** (1.008 g). The filtrate was then concentrated under reduced pressure, water (50 mL) was added, and the mixture was extracted with dichloromethane (3 x 50 mL). The combined organic fractions were dried over anhydrous magnesium sulfate and the solvent was removed under reduced pressure. The residue was purified by column chromatography (silica gel, cyclohexane/ ethyl acetate, gradient: 8% to 60% ethyl acetate *v*/*v*). Overall, **3** (1.697 g, 3.9 mmol, 87%) was obtained as a colorless solid.

^{1}H NMR (400 MHz, $CDCl_3$): δ = 7.79 – 7.68 (m, 6H), 7.61 – 7.53 (m, 3H), 7.53 – 7.47 (m, 2H), 7.47 – 7.39 (m, 6H), 7.04 – 6.96 (m, 2H), 2.29 (s, 3H) ppm.

^{13}C NMR (101 MHz, $CDCl_3$): δ = 143.6 (d, *J* = 2.7 Hz), 140.6, 133.3 (d, *J* = 10.8 Hz), 132.9 (d, *J* = 2.5 Hz), 128.8 (d, *J* = 13.1 Hz), 128.7, 127.5 (d, *J* = 104.2 Hz), 125.8, 21.4 ppm.

^{31}P NMR (162 MHz, $CDCl_3$): δ = 14.49 (s) ppm.

HRMS for $C_{25}H_{22}NO_2PS$ (ESI+) $[M+H]^+$ m/z: calc: 432.1182, found: 432.1183.

The analytical data are in accordance with the literature.[13]

4-Methyl-*N*-(tri-*o*-tolyl-λ^5-phosphanylidene)benzenesulfonamide (4)

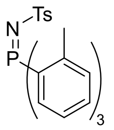

According to general protocol **A**, a suspension of tri(*o*-tolyl)phosphine (1.370 g, 4.5 mmol, 1.0 eq.), *p*-toluenesulfonamide ($TsNH_2$, 0.771 g, 4.5 mmol, 1.0 eq.), and NEt_4I (0.154 g, 0.6 mmol) in anhydrous MeCN (15 mL) was electrolyzed in an undivided 25 mL batch-type glass cell under inert conditions, applying 2.0 *F* of electric charge. The product precipitated from the electrolyte, was filtered off and washed with acetonitrile, yielding pure **4** (1.270 g). The filtrate was then concentrated under reduced pressure, water (50 mL) was added, and the mixture was extracted with dichloromethane (3 x 40 mL). The combined organic fractions were dried over anhydrous magnesium sulfate and the solvent was removed under reduced pressure. Purification by column chromatography (silica gel, cyclohexane/ ethyl acetate, gradient: 4% to 62% ethyl acetate *v*/*v*) yielded **4** with small residues of $TsNH_2$ and the corresponding phosphine oxide. Overall, **4** (1.717 g, 3.6 mmol, 81%) was obtained as a colorless solid.

^{1}H NMR (400 MHz, $CDCl_3$): δ = 7.77 (ddd, *J* = 15.6, 7.8, 1.5 Hz, 3H), 7.65 – 7.61 (m, 2H), 7.48 (tt, *J* = 7.4, 1.5 Hz, 3H), 7.33 – 7.26 (m, 3H), 7.26 – 7.20 (m, 3H), 7.13 – 7.07 (m, 2H), 2.35 (s, 3H), 2.16 (s, 9H) ppm.

^{13}C NMR (101 MHz, $CDCl_3$): δ = 143.6 (d, *J* = 5.5 Hz), 143.3 (d, *J* = 8.9 Hz), 140.6, 134.9 (d, *J* = 13.7 Hz), 132.9 (d, *J* = 2.9 Hz), 132.5 (d, *J* = 11.3 Hz), 128.8, 126.1 (d, *J* = 13.8 Hz), 126.0, 125.4 (d, *J* = 101.6 Hz), 22.6 (d, *J* = 4.0 Hz), 21.5 ppm.

^{31}P NMR (162 MHz, $CDCl_3$): δ = 17.74 (s) ppm.

HRMS for $C_{28}H_{28}NO_2PS$ (ESI+) $[M+H]^+$ m/z: calc: 474.1651, found: 474.1660.

4-Methyl-*N*-(tris(4-fluorophenyl)-λ^5-phosphanylidene)benzenesulfonamide (5)

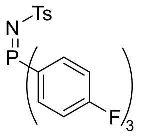

According to general protocol **A**, a solution of tris(4-fluorophenyl)phosphine (1.423 g, 4.5 mmol, 1.0 eq.), *p*-toluenesulfonamide (0.771 g, 4.5 mmol, 1.0 eq.), and NEt_4I (0.154 g, 0.6 mmol) in anhydrous MeCN (15 mL) was electrolyzed in an undivided 25 mL batch-type glass cell under inert conditions, applying 2.0 *F* of electric charge. Purification by column chromatography (silica gel, cyclohexane/ ethyl acetate, gradient: 10% to 50% ethyl acetate *v*/*v*) yielded **5** (1.666 g, 3.4 mmol, 76%) as a colorless solid.

^{1}H NMR (400 MHz, $CDCl_3$): δ = 7.77 – 7.66 (m, 6H), 7.51 – 7.45 (m, 2H), 7.19 – 7.11 (m, 6H), 7.06 – 7.01 (m, 2H), 2.31 (s, 3H) ppm.

^{13}C NMR (101 MHz, $CDCl_3$): δ = 165.7 (dd, *J* = 256.4, 3.4 Hz), 143.1 (d, *J* = 2.9 Hz), 141.0, 135.7 (dd, *J* = 12.5, 9.1 Hz), 128.8, 125.7, 123.0 (dd, *J* = 109.0, 3.4 Hz), 116.5 (dd, *J* = 21.7, 14.3 Hz), 21.4 ppm.

^{31}P NMR (162 MHz, $CDCl_3$): δ = 12.49 (s) ppm.

^{19}F NMR (376 MHz, $CDCl_3$): δ = (-104.05) – (-104.17) (m) ppm.

HRMS for $C_{25}H_{19}F_3NO_2PS$ (ESI+) $[M+H]^+$ m/z: calc: 486.0899, found: 486.0902.

4-Methyl-*N*-(tri(furan-2-yl)-λ^5-phosphanylidene)benzenesulfonamide (6)

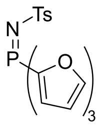

According to general protocol **A**, a solution of tri(2-furyl)phosphine (1.045 g, 4.5 mmol, 1.0 eq.), *p*-toluenesulfonamide (0.771 g, 4.5 mmol, 1.0 eq.), and NEt_4I (0.154 g, 0.6 mmol) in anhydrous MeCN (15 mL) was electrolyzed in an undivided 25 mL batch-type glass cell under inert conditions, applying 2.4 *F* of electric charge. Purification by column chromatography (silica gel, cyclohexane/ ethyl acetate, gradient: 12% to 93% ethyl acetate *v*/*v*; then: silica gel, cyclohexane/ ethyl acetate, gradient: 40% to 50% ethyl acetate *v*/*v*) yielded **6** (1.054 g, 2.6 mmol, 58%) as a colorless solid.

^{1}H NMR (400 MHz, $CDCl_3$): δ = 7.72 (ddd, *J* = 2.9, 1.8, 0.7 Hz, 3H), 7.68 – 7.63 (m, 2H), 7.36 (ddd, *J* = 3.6, 2.1, 0.7 Hz, 3H), 7.15 – 7.10 (m, 2H), 6.57 (dt, *J* = 3.6, 1.8 Hz, 3H), 2.34 (s, 3H) ppm.

^{13}C NMR (101 MHz, $CDCl_3$): δ = 150.1 (d, *J* = 9.3 Hz), 142.4 (d, *J* = 2.5 Hz), 141.0, 140.4 (d, *J* = 162.9 Hz), 128.7, 126.4 (d, *J* = 22.6 Hz), 125.7, 111.6 (d, *J* = 9.9 Hz), 21.2 ppm.

^{31}P NMR (162 MHz, $CDCl_3$): δ = -27.52 (s) ppm.

HRMS for $C_{19}H_{16}NO_5PS$ (ESI+) $[M+H]^+$ m/z: calc: 402.0560, found: 402.0563.

The analytical data are in accordance with the literature.[14]

4-Methyl-*N*-(tributyl-λ^5-phosphanylidene)benzenesulfonamide (7)

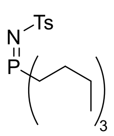

According to general protocol **A**, a solution of tributylphosphine (1.11 mL, 4.5 mmol, 1.0 eq.), *p*-toluenesulfonamide (0.771 g, 4.5 mmol, 1.0 eq.), and NEt_4I (0.154 g, 0.6 mmol) in anhydrous MeCN (15 mL) was electrolyzed in an undivided 25 mL batch-type glass cell under inert conditions, applying 2.0 *F* of electric charge. Purification by column chromatography (silica gel, cyclohexane/ ethyl acetate, gradient: 15% to 50% ethyl acetate *v*/*v*; then: silica gel, cyclohexane/ (ethyl acetate + 9% triethylamine, *v*/*v*), gradient: 15% to 25% (ethyl acetate + 9% triethylamine) *v*/*v*) yielded **7** (1.424 g, 3.8 mmol, 85%) as a light yellow solid.

^{1}H NMR (400 MHz, $CDCl_3$): δ = 7.81 – 7.72 (m, 2H), 7.20 – 7.12 (m, 2H), 2.34 (s, 3H), 1.92 – 1.81 (m, 6H), 1.49 – 1.27 (m, 12H), 0.85 (t, *J* = 7.2 Hz, 9H) ppm.

^{13}C NMR (101 MHz, $CDCl_3$): δ = 144.0 (d, *J* = 2.5 Hz), 140.8, 128.9, 125.7, 24.9 (d, *J* = 62.5 Hz), 24.0 (d, *J* = 15.6 Hz), 23.6 (d, *J* = 4.1 Hz), 21.4, 13.5 ppm.

^{31}P NMR (162 MHz, $CDCl_3$): δ = 36.50 (s) ppm.

HRMS for $C_{19}H_{34}NO_2PS$ (ESI+) $[M+H]^+$ m/z: calc: 372.2121, found: 372.2124.

4-Methyl-*N*-(tricyclohexyl-λ^5-phosphanylidene)benzenesulfonamide (8)

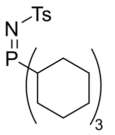

According to general protocol **A**, a solution of tricyclohexylphosphine (1.262 g, 4.5 mmol, 1.0 eq.), *p*-toluenesulfonamide (0.771 g, 4.5 mmol, 1.0 eq.), and NEt_4I (0.154 g, 0.6 mmol) in anhydrous MeCN (15 mL) was electrolyzed in an undivided 25 mL batch-type glass cell under inert conditions, applying 2.0 *F* of electric charge. The crude product was recrystallized from boiling ethyl acetate, followed by purification of the residual filtrate by column chromatography (silica gel, cyclohexane/ ethyl acetate, gradient: 8% to 62% ethyl acetate *v*/*v*), yielding **8** (1.728 g, 3.8 mmol, 85%) as a colorless solid.

^{1}H NMR (400 MHz, $CDCl_3$): δ = 7.80 – 7.69 (m, 2H), 7.16 – 7.06 (m, 2H), 2.29 (s, 3H), 2.25 – 2.09 (m, 3H), 1.96 – 1.57 (m, 15H), 1.54 – 1.34 (m, 6H), 1.27 – 1.05 (m, 9H) ppm.

^{13}C NMR (101 MHz, $CDCl_3$): δ = 144.3 (d, *J* = 5.5 Hz), 140.2, 128.6, 125.5, 34.2 (d, *J* = 57.1 Hz), 26.7 (d, *J* = 12.5 Hz), 26.5 (d, *J* = 3.0 Hz), 25.7, 21.2 ppm.

^{31}P NMR (162 MHz, $CDCl_3$): δ = 38.41 (s) ppm.

HRMS for $C_{25}H_{40}NO_2PS$ (ESI+) $[M+H]^+$ m/z: calc: 450.2590, found: 450.2593.

The ^{13}C NMR and ^{31}P NMR data are in accordance with the literature.[15]

4-Methyl-*N*-(tris(2-cyanoethyl)-λ^5-phosphanylidene)benzenesulfonamide (10)

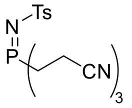

According to general protocol **A**, a suspension of tris(2-cyanoethyl)phosphine (0.869 g, 4.5 mmol, 1.0 eq.), *p*-toluenesulfonamide (0.771 g, 4.5 mmol, 1.0 eq.), and NEt_4I (0.154 g, 0.6 mmol) in anhydrous MeCN (15 mL) was electrolyzed in an undivided 25 mL batch-type glass cell under inert conditions, applying 2.0 *F* of electric charge. The crude product was recrystallized from boiling ethyl acetate, followed by purification of the residual filtrate by column chromatography (silica gel, dichloromethane/ MeCN, gradient: 0% to 64% MeCN *v*/*v*), yielding **10** (1.235 g, 3.4 mmol, 76%) as a colorless solid.

^{1}H NMR (600 MHz, CD_2Cl_2): δ = 7.78 – 7.73 (m, 2H), 7.32 – 7.28 (m, 2H), 2.86 (dt, *J* = 14.9, 7.3 Hz, 6H), 2.53 (dt, *J* = 10.7, 7.3 Hz, 6H), 2.41 (s, 3H) ppm.

^{13}C NMR (151 MHz, CD_2Cl_2): δ = 143.0, 142.0 (d, *J* = 5.7 Hz), 129.8, 125.8, 118.2 (d, *J* = 11.7 Hz), 23.5 (d, *J* = 63.6 Hz), 21.5, 11.1 (d, *J* = 3.7 Hz) ppm.

^{31}P NMR (162 MHz, CD_2Cl_2): δ = 30.33 (s) ppm.

HRMS for $C_{16}H_{19}N_4O_2PS$ (ESI+) $[M+Na]^+$ m/z: calc: 385.0859, found: 385.0859.

***N*,*N'*-(Ethane-1,2-diylbis(diphenyl-λ^5-phosphanylylidene))bis(4-methylbenzenesulfonamide) (11)**

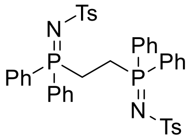

According to general protocol **A**, a suspension of 1,2-bis(diphenylphosphino)ethane (dppe, 0.598 g, 1.5 mmol, 1.0 eq.), *p*-toluenesulfonamide (0.514 g, 3.0 mmol, 2.0 eq.), and NEt_4I (0.154 g, 0.6 mmol) in anhydrous MeCN (15 mL) was electrolyzed in an undivided 25 mL batch-type glass cell under inert conditions (stirring speed: 500 rpm), applying 4.0 *F* of electric charge. The lower amounts of starting materials and the higher stirring speed were applied due to the poor solubility of dppe. After completed electrolysis the reaction mixture was concentrated and the residue was purified by column chromatography (silica gel, dichloromethane/ ethyl acetate, gradient: 12% to 93% ethyl acetate *v*/*v*), yielding **11** (0.753 g, 1.0 mmol, 68%) as a colorless solid.

^{1}H NMR (400 MHz, $CDCl_3$): δ = 7.77 – 7.69 (m, 8H), 7.63 – 7.58 (m, 4H), 7.57 – 7.51 (m, 4H), 7.47 – 7.40 (m, 8H), 7.11 – 7.04 (m, 4H), 3.12 (d, *J* = 2.9 Hz, 4H), 2.33 (s, 6H) ppm.

^{13}C NMR (101 MHz, $CDCl_3$): δ = 143.0, 141.1, 133.1, 131.9 (t, *J* = 5.3 Hz), 129.2 (t, *J* = 6.4 Hz), 129.0, 127.3 – 125.6 (m), 125.8, 21.5, 21.0 – 20.3 (m) ppm.

^{31}P NMR (162 MHz, $CDCl_3$): δ = 20.60 (s) ppm.

HRMS for $C_{40}H_{38}N_2O_4P_2S_2$ (ESI+) $[M+H]^+$ m/z: calc: 737.1821, found: 737.1824.

The analytical data are in accordance with the literature.[16]

***N*-(Ethyldiphenyl-λ^5-phosphanylidene)-4-methylbenzenesulfonamide (12)**

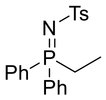

According to general protocol **A**, a solution of ethyldiphenylphosphine (0.92 mL, 4.5 mmol, 1.0 eq.), *p*-toluenesulfonamide (0.771 g, 4.5 mmol, 1.0 eq.), and NEt_4I (0.154 g, 0.6 mmol) in anhydrous MeCN (15 mL) was electrolyzed in an undivided 25 mL batch-type glass cell under inert conditions, applying 2.0 *F* of electric charge. The crude product was purified by addition of ethyl acetate (30 mL), leading to partial crystallization of **12**, which was filtered off. The filtrate was then purified by column chromatography (silica gel, cyclohexane/ ethyl acetate, gradient: 18% to 93% ethyl acetate *v*/*v*), yielding **12** (1.389 g, 3.6 mmol, 80%) as a colorless solid.

^{1}H NMR (400 MHz, $CDCl_3$): δ = 7.70 – 7.61 (m, 4H), 7.58 – 7.48 (m, 4H), 7.45 – 7.36 (m, 4H), 7.04 – 6.97 (m, 2H), 2.68 (dq, *J* = 11.8, 7.5 Hz, 2H), 2.29 (s, 3H), 1.14 (dt, *J* = 19.1, 7.5 Hz, 3H) ppm.

^{13}C NMR (101 MHz, $CDCl_3$): δ = 143.3 (d, *J* = 2.5 Hz), 140.6, 132.7 (d, *J* = 2.9 Hz), 131.9 (d, *J* = 10.2 Hz), 128.9 (d, *J* = 12.3 Hz), 128.7, 126.9 (d, *J* = 101.7 Hz), 125.7, 21.4, 20.6 (d, *J* = 64.6 Hz), 5.9 (d, *J* = 4.7 Hz) ppm.

^{31}P NMR (162 MHz, $CDCl_3$): δ = 22.82 (s) ppm.

HRMS for $C_{21}H_{22}NO_2PS$ (ESI+) $[M+H]^+$ m/z: calc: 384.1182, found: 384.1187.

N-(Diphenyl(4-vinylphenyl)-λ⁵-phosphanylidene)-4-methylbenzenesulfonamide (13) and N-((4-Ethylphenyl)diphenyl-λ⁵-phosphanylidene)-4-methylbenzenesulfonamide (13')

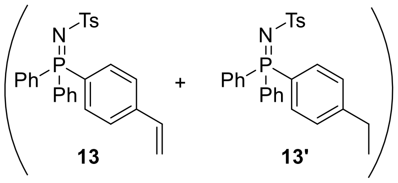

According to general protocol **A**, a suspension of 4-(diphenylphosphino)styrene (1.297 g, 4.5 mmol, 1.0 eq.), *p*-toluenesulfonamide (0.771 g, 4.5 mmol, 1.0 eq.), and NEt_4I (0.154 g, 0.6 mmol) in anhydrous MeCN (15 mL) was electrolyzed in an undivided 25 mL batch-type glass cell under inert conditions, applying 2.0 *F* of electric charge. Purification by reversed phase column chromatography (C18 silica gel, water/ MeCN, gradient: 50% to 60% MeCN *v*/*v*) yielded **13** (0.705 g, 1.5 mmol, 34%) and **13'** (0.521 g, 1.1 mmol, 25%) as colorless solids.

Compound 13:

^{1}H NMR (400 MHz, $CDCl_3$): δ = 7.76 – 7.59 (m, 6H), 7.53 – 7.43 (m, 4H), 7.42 – 7.33 (m, 6H), 6.97 – 6.88 (m, 2H), 6.65 (dd, *J* = 17.6, 10.9 Hz, 1H), 5.79 (d, *J* = 17.6 Hz, 1H), 5.33 (d, *J* = 10.9 Hz, 1H), 2.21 (s, 3H) ppm.

^{13}C NMR (101 MHz, $CDCl_3$): δ = 143.2 (d, *J* = 2.3 Hz), 141.6 (d, *J* = 3.0 Hz), 140.3, 135.4 (d, *J* = 1.7 Hz), 133.2 (d, *J* = 11.1 Hz), 132.8 (d, *J* = 10.8 Hz), 132.6 (d, *J* = 2.9 Hz), 128.5 (d, *J* = 13.0 Hz), 128.4, 127.0 (d, *J* = 104.7 Hz), 126.1 (d, *J* = 13.4 Hz), 125.8 (d, *J* = 105.3 Hz), 125.5, 117.2, 21.1 ppm.

^{31}P NMR (162 MHz, $CDCl_3$): δ = 14.13 (s) ppm.

HRMS for $C_{27}H_{24}NO_2PS$ (ESI+) $[M+H]^+$ m/z: calc: 458.1338, found: 458.1341.

Compound 13':

^{1}H NMR (400 MHz, $CDCl_3$): δ = 7.78 – 7.69 (m, 4H), 7.66 – 7.59 (m, 2H), 7.58 – 7.52 (m, 2H), 7.51 – 7.46 (m, 2H), 7.46 – 7.40 (m, 4H), 7.28 – 7.23 (m, 2H), 7.01 – 6.95 (m, 2H), 2.69 (q, *J* = 7.6 Hz, 2H), 2.28 (s, 3H), 1.24 (t, *J* = 7.6 Hz, 3H) ppm.

^{13}C NMR (101 MHz, $CDCl_3$): δ = 149.6 (d, *J* = 2.9 Hz), 143.4 (d, *J* = 2.4 Hz), 140.3, 133.2 (d, *J* = 11.1 Hz), 133.0 (d, *J* = 10.8 Hz), 132.6 (d, *J* = 3.0 Hz), 128.6 (d, *J* = 12.8 Hz), 128.5, 128.2 (d, *J* = 13.3 Hz), 127.5 (d, *J* = 104.7 Hz), 125.6, 123.8 (d, *J* = 105.8 Hz), 28.8, 21.2, 15.1 ppm.

^{31}P NMR (162 MHz, $CDCl_3$): δ = 14.39 (s) ppm.

HRMS for $C_{27}H_{26}NO_2PS$ (ESI+) $[M+H]^+$ m/z: calc: 460.1495, found: 460.1496.

4-Methoxy-*N*-(triphenyl-λ⁵-phosphanylidene)benzenesulfonamide (14)

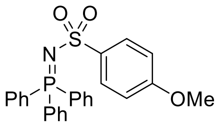

According to general protocol **A**, a solution of triphenylphosphine (1.180 g, 4.5 mmol, 1.0 eq.), 4-methoxybenzenesulfonamide (0.842 g, 4.5 mmol, 1.0 eq.), and NEt_4I (0.154 g, 0.6 mmol) in anhydrous MeCN (15 mL) was electrolyzed in an undivided 25 mL batch-type glass cell under inert conditions, applying 2.0 *F* of electric charge. Purification by column chromatography (silica gel, cyclohexane/ ethyl acetate, gradient: 20% to 100% ethyl acetate *v*/*v*) yielded **14** (1.628 g, 3.6 mmol, 81%) as a colorless solid.

^{1}H NMR (400 MHz, $CDCl_3$): δ = 7.78 – 7.68 (m, 6H), 7.59 – 7.49 (m, 5H), 7.47 – 7.39 (m, 6H), 6.70 – 6.64 (m, 2H), 3.74 (s, 3H) ppm.

^{13}C NMR (101 MHz, $CDCl_3$): δ = 161.0, 138.7 (d, *J* = 2.6 Hz), 133.2 (d, *J* = 10.7 Hz), 132.8 (d, *J* = 3.0 Hz), 128.8 (d, *J* = 12.8 Hz), 127.6, 127.3 (d, *J* = 104.2 Hz), 113.2, 55.4 ppm.

^{31}P NMR (162 MHz, $CDCl_3$): δ = 14.24 (s) ppm.

HRMS for $C_{25}H_{22}NO_3PS$ (ESI+) $[M+H]^+$ m/z: calc: 448.1131, found: 448.1131.

The analytical data are in accordance with the literature.[17]

4-Chloro-*N*-(triphenyl-λ⁵-phosphanylidene)benzenesulfonamide (15)

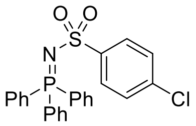

According to general protocol **A**, a solution of triphenylphosphine (1.180 g, 4.5 mmol, 1.0 eq.), 4-chlorobenzenesulfonamide (0.862 g, 4.5 mmol, 1.0 eq.), and NEt_4I (0.154 g, 0.6 mmol) in anhydrous MeCN (15 mL) was electrolyzed in an undivided 25 mL batch-type glass cell under inert conditions, applying 2.0 *F* of electric charge. Purification by column chromatography (silica gel, cyclohexane/ ethyl acetate, gradient: 7% to 35% ethyl acetate *v*/*v*, then: silica gel, cyclohexane/ ethyl acetate/ dichloromethane, 4:1:1 *v*/*v*/*v*) yielded **15** (0.401 g, 0.9 mmol, 20%) as a colorless solid.

^{1}H NMR (400 MHz, $CDCl_3$): δ = 7.79 – 7.68 (m, 6H), 7.63 – 7.55 (m, 3H), 7.54 – 7.41 (m, 8H), 7.19 – 7.11 (m, 2H) ppm.

^{13}C NMR (101 MHz, $CDCl_3$): δ = 144.8 (d, *J* = 2.4 Hz), 136.4, 133.2 (d, *J* = 10.8 Hz), 133.1 (d, *J* = 3.0 Hz), 128.9 (d, *J* = 13.0 Hz), 128.3, 127.4, 127.1 (d, *J* = 104.1 Hz) ppm.

^{31}P NMR (162 MHz, $CDCl_3$): δ = 15.07 (s) ppm.

HRMS for $C_{24}H_{19}ClNO_2PS$ (ESI+) $[M+H]^+$ m/z: calc: 452.0635, found: 452.0634.

The analytical data are in accordance with the literature.[17]

4-Amino-*N*-(triphenyl-*λ*⁵-phosphanylidene)benzenesulfonamide (16)

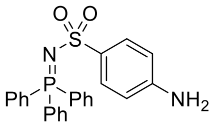

According to general protocol **A**, a solution of triphenylphosphine (1.180 g, 4.5 mmol, 1.0 eq.), sulfanilamide (0.775 g, 4.5 mmol, 1.0 eq.), and NEt_4I (0.154 g, 0.6 mmol) in anhydrous MeCN (15 mL) was electrolyzed in an undivided 25 mL batch-type glass cell under inert conditions, applying 2.0 *F* of electric charge. Purification by reversed phase column chromatography (C18 silica gel, water/ MeCN, gradient: 30% to 43% MeCN *v*/*v*) yielded **16** (0.871 g, 2.0 mmol, 45%) as a colorless solid.

^{1}H NMR (400 MHz, $CDCl_3$): δ = 7.79 – 7.68 (m, 6H), 7.60 – 7.52 (m, 3H), 7.48 – 7.41 (m, 6H), 7.41 – 7.34 (m, 2H), 6.46 – 6.40 (m, 2H), 3.73 (s broad, 2H) ppm.

^{13}C NMR (101 MHz, $CDCl_3$): δ = 148.3, 136.2 (d, *J* = 2.5 Hz), 133.3 (d, *J* = 10.7 Hz), 132.8 (d, *J* = 3.0 Hz), 128.8 (d, *J* = 12.9 Hz), 127.6 (d, *J* = 104.1 Hz), 127.5, 113.8 ppm.

^{31}P NMR (162 MHz, $CDCl_3$): δ = 13.80 (s) ppm.

HRMS for $C_{24}H_{21}N_2O_2PS$ (ESI+) $[M+H]^+$ m/z: calc: 433.1134, found: 433.1133.

The analytical data are in accordance with the literature.[17]

4-Nitro-*N*-(triphenyl-*λ*⁵-phosphanylidene)benzenesulfonamide (17)

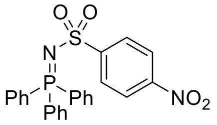

According to general protocol **A**, a solution of triphenylphosphine (1.180 g, 4.5 mmol, 1.0 eq.), 4-nitrobenzenesulfonamide (0.910 g, 4.5 mmol, 1.0 eq.), and NEt_4I (0.154 g, 0.6 mmol) in anhydrous MeCN (15 mL) was electrolyzed in an undivided 25 mL batch-type glass cell under inert conditions, applying 2.0 *F* of electric charge. Purification by column chromatography (silica gel, cyclohexane/ ethyl acetate/ dichloromethane, gradient: 15%/ 15%/ 70% to 25%/ 25%/ 50% *v*/*v*) yielded **17** (0.530 g, 1.2 mmol, 25%) as a light green solid.

^{1}H NMR (400 MHz, $CDCl_3$): δ = 8.06 – 7.98 (m, 2H), 7.77 – 7.67 (m, 8H), 7.63 – 7.54 (m, 3H), 7.50 – 7.40 (m, 6H) ppm.

^{13}C NMR (101 MHz, $CDCl_3$): δ = 151.7 (d, *J* = 2.3 Hz), 148.5, 133.2 (d, *J* = 3.0 Hz), 133.0 (d, *J* = 10.8 Hz), 129.0 (d, *J* = 13.0 Hz), 127.0, 126.5 (d, *J* = 104.5 Hz), 123.4 ppm.

^{31}P NMR (162 MHz, $CDCl_3$): δ = 16.24 (s) ppm.

HRMS for $C_{24}H_{19}N_2O_4PS$ (ESI+) $[M+H]^+$ m/z: calc: 463.0876, found: 463.0874.

The analytical data are in accordance with the literature.[17]

***N*-(Triphenyl-*λ*⁵-phosphanylidene)benzenesulfonamide (18)**

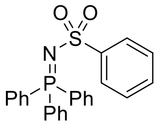

According to general protocol **A**, a solution of triphenylphosphine (1.180 g, 4.5 mmol, 1.0 eq.), benzenesulfonamide (0.707 g, 4.5 mmol, 1.0 eq.), and NEt_4I (0.154 g, 0.6 mmol) in anhydrous MeCN (15 mL) was electrolyzed in an undivided 25 mL batch-type glass cell under inert conditions, applying 2.0 *F* of electric charge. The crude product was recrystallized from boiling ethyl acetate, followed by purification of the residual filtrate by column chromatography (silica gel, cyclohexane/ ethyl acetate, gradient: 8% to 50% ethyl acetate *v*/*v*), yielding **18** (1.524 g, 3.7 mmol, 81%) as a colorless solid.

^{1}H NMR (400 MHz, $CDCl_3$): δ = 7.79 – 7.69 (m, 6H), 7.64 – 7.53 (m, 5H), 7.49 – 7.40 (m, 6H), 7.32 – 7.26 (m, 1H), 7.23 – 7.16 (m, 2H) ppm.

^{13}C NMR (101 MHz, $CDCl_3$): δ = 146.1 (d, *J* = 2.3 Hz), 133.2 (d, *J* = 10.7 Hz), 132.9 (d, *J* = 3.0 Hz), 130.3, 128.8 (d, *J* = 12.9 Hz), 128.1, 127.2 (d, *J* = 104.3 Hz), 125.8 ppm.

^{31}P NMR (162 MHz, $CDCl_3$): δ = 14.82 (s) ppm.

HRMS for $C_{24}H_{20}NO_2PS$ (ESI+) $[M+H]^+$ m/z: calc: 418.1025, found: 418.1016.

The analytical data are in accordance with the literature.[17,18]

2,4,6-Triisopropyl-*N*-(triphenyl-*λ*⁵-phosphanylidene)benzenesulfonamide (19)

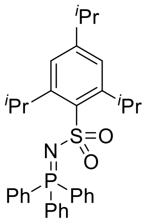

According to general protocol **A**, a solution of triphenylphosphine (1.180 g, 4.5 mmol, 1.0 eq.), 2,4,6-triisopropylbenzenesulfonamide (**24**, 1.275 g, 4.5 mmol, 1.0 eq.), and NEt_4I (0.154 g, 0.6 mmol) in anhydrous MeCN (15 mL) was electrolyzed in an undivided 25 mL batch-type glass cell under inert conditions, applying 2.0 *F* of electric charge. Purification by column chromatography (silica gel, cyclohexane/ ethyl acetate, gradient: 4% to 38% ethyl acetate *v*/*v*) yielded **19** (2.210 g, 4.1 mmol, 90%) as a colorless solid.

^{1}H NMR (400 MHz, $CDCl_3$): δ = 7.74 – 7.64 (m, 6H), 7.57 – 7.49 (m, 3H), 7.45 – 7.37 (m, 6H), 6.97 (s, 2H), 4.38 (hept, *J* = 6.8 Hz, 2H), 2.83 (hept, *J* = 7.0 Hz, 1H), 1.22 (d, *J* = 7.0 Hz, 6H), 1.10 (d, *J* = 6.8 Hz, 12H) ppm.

^{13}C NMR (101 MHz, $CDCl_3$): δ = 150.2, 148.2, 140.1 (d, *J* = 2.9 Hz), 133.3 (d, *J* = 10.7 Hz), 132.6 (d, *J* = 3.0 Hz), 128.7 (d, *J* = 13.0 Hz), 128.2 (d, *J* = 104.0 Hz), 122.7, 34.2, 29.3, 25.0, 24.0 ppm.

^{31}P NMR (162 MHz, $CDCl_3$): δ = 14.86 (s) ppm.

HRMS for $C_{33}H_{38}NO_2PS$ (ESI+) $[M+H]^+$ m/z: calc: 544.2434, found: 544.2431.

N-(Triphenyl-λ⁵-phosphanylidene)methanesulfonamide (20)

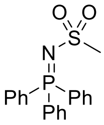

According to general protocol **A**, a solution of triphenylphosphine (1.180 g, 4.5 mmol, 1.0 eq.), methanesulfonamide (0.428 g, 4.5 mmol, 1.0 eq.), and NEt_4I (0.154 g, 0.6 mmol) in anhydrous MeCN (15 mL) was electrolyzed in an undivided 25 mL batch-type glass cell under inert conditions, applying 2.0 *F* of electric charge. The product precipitated from the electrolyte, was filtered off, and washed with acetonitrile, yielding pure **20** (0.456 g). The filtrate was then concentrated under reduced pressure, water (50 mL) was added, and the mixture was extracted with dichloromethane (3 x 50 mL). The combined organic fractions were dried over anhydrous magnesium sulfate and the solvent was removed under reduced pressure. The crude product was recrystallized from boiling ethyl acetate, followed by purification of the residual filtrate by column chromatography (silica gel, cyclohexane/ ethyl acetate, gradient: 50% to 75% ethyl acetate *v*/*v*), yielding **20** (1.210 g, 3.4 mmol, 76%) as a colorless solid.

^{1}H NMR (400 MHz, $CDCl_3$): *δ* = 7.87 – 7.74 (m, 6H), 7.66 – 7.56 (m, 3H), 7.56 – 7.43 (m, 6H), 2.75 (d, *J* = 1.2 Hz, 3H) ppm.
^{13}C NMR (101 MHz, $CDCl_3$): *δ* = 133.2 (d, *J* = 10.8 Hz), 133.0 (d, *J* = 3.0 Hz), 128.9 (d, *J* = 12.8 Hz), 127.6 (d, *J* = 104.2 Hz), 44.4 (d, *J* = 3.0 Hz) ppm.
^{31}P NMR (162 MHz, $CDCl_3$): *δ* = 14.93 (s) ppm.
HRMS for $C_{19}H_{18}NO_2PS$ (ESI+) $[M+H]^+$ m/z: calc: 356.0869, found: 356.0870.

1,1,1-Trifluoro-N-(triphenyl-λ⁵-phosphanylidene)methanesulfonamide (21)

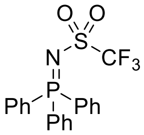

According to general protocol **A**, a solution of triphenylphosphine (1.180 g, 4.5 mmol, 1.0 eq.), trifluoromethanesulfonamide (0.671 g, 4.5 mmol, 1.0 eq.), and NEt_4I (0.154 g, 0.6 mmol) in anhydrous MeCN (15 mL) was electrolyzed in an undivided 25 mL batch-type glass cell under inert conditions, applying 2.0 *F* of electric charge. Purification by column chromatography (silica gel, cyclohexane/ ethyl acetate, gradient: 4% to 64% ethyl acetate *v*/*v*) yielded **21** (1.604 g, 3.9 mmol, 87%) as a colorless solid.

^{1}H NMR (400 MHz, $CDCl_3$): *δ* = 7.81 – 7.71 (m, 6H), 7.70 – 7.61 (m, 3H), 7.59 – 7.48 (m, 6H) ppm.
^{13}C NMR (101 MHz, $CDCl_3$): *δ* = 133.7, 133.0 (d, *J* = 11.1 Hz), 129.1 (d, *J* = 13.3 Hz), 125.8 (d, *J* = 105.7 Hz), 120.0 (qd, *J* = 320.4, 5.9 Hz) ppm.
^{31}P NMR (162 MHz, $CDCl_3$): *δ* = 20.51 (s) ppm.
^{19}F NMR (376 MHz, $CDCl_3$): *δ* = -80.55 (s) ppm.
HRMS for $C_{19}H_{15}F_3NO_2PS$ (ESI+) $[M+H]^+$ m/z: calc: 410.0586, found: 410.0590.
The analytical data are in accordance with the literature.[19,20]

(1S)-10-Camphor-(N-(triphenyl-λ⁵-phosphanylidene))sulfonamide (22)

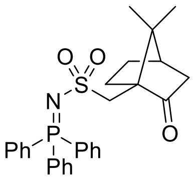

According to general protocol **A**, a solution of triphenylphosphine (1.180 g, 4.5 mmol, 1.0 eq.), (1*S*)-10-camphorsulfonamide (**25**, 1.041 g, 4.5 mmol, 1.0 eq.), and NEt_4I (0.154 g, 0.6 mmol) in anhydrous MeCN (15 mL) was electrolyzed in an undivided 25 mL batch-type glass cell under inert conditions, applying 2.0 *F* of electric charge. The product precipitated from the electrolyte, was filtered off and washed with acetonitrile, yielding pure **22** (1.292 g). The filtrate was then concentrated under reduced pressure, water (50 mL) was added, and the mixture was extracted with dichloromethane (3 x 50 mL). The combined organic fractions were dried over anhydrous magnesium sulfate and the solvent was removed under reduced pressure. The residue was purified by column chromatography (silica gel, cyclohexane/ ethyl acetate, gradient: 8% to 50% ethyl acetate *v*/*v*), yielding **22** (1.599 g, 3.3 mmol, 72%) as a colorless solid.

^{1}H NMR (400 MHz, $CDCl_3$): *δ* = 7.86 – 7.75 (m, 6H), 7.63 – 7.55 (m, 3H), 7.54 – 7.45 (m, 6H), 3.38 (d, *J* = 14.8 Hz, 1H), 2.80 – 2.69 (m, 2H), 2.27 (dt, *J* = 18.2, 4.1 Hz, 1H), 2.03 – 1.91 (m, 2H), 1.83 (d, *J* = 18.2 Hz, 1H), 1.56* – 1.48 (m, 1H), 1.36 – 1.27 (m, 1H), 1.07 (s, 3H), 0.71 (s, 3H) ppm.
^{13}C NMR (101 MHz, $CDCl_3$): *δ* = 216.2, 133.3 (d, *J* = 10.7 Hz), 132.9 (d, *J* = 2.9 Hz), 128.9 (d, *J* = 12.9 Hz), 127.9 (d, *J* = 104.2 Hz), 59.0, 52.4 (d, *J* = 3.1 Hz), 47.8, 42.9**, 42.8**, 27.1, 24.7, 20.4, 19.8 ppm.
^{31}P NMR (162 MHz, $CDCl_3$): *δ* = 15.09 (s) ppm.
HRMS for $C_{28}H_{30}NO_3PS$ (ESI+) $[M+H]^+$ m/z: calc: 492.1757, found: 492.1769.
* Signal partially overlaps with the water signal.
** Separate signals, which overlap due to the similar chemical shift.

3,3,5,5-Tetraphenyl-4H-1,2,6,3λ⁵,5λ⁵-thiadiazadiphosphanine-1,1-dioxide (23)

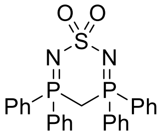

According to general protocol **A**, a suspension of 1,1-bis(diphenylphosphino)methane (1.730 g, 4.5 mmol, 1.0 eq.), sulfamide (0.433 g, 4.5 mmol, 1.0 eq.), and NEt_4I (0.154 g, 0.6 mmol) in anhydrous MeCN (15 mL) was electrolyzed in an undivided 25 mL batch-type glass cell under inert conditions, applying 4.0 *F* of electric charge. The reaction mixture was concentrated, followed by purification by reversed phase column chromatography (C18 silica gel, water/ MeCN, gradient: 20% to 30% acetonitrile *v*/*v*), yielding **23** (0.419 g, 0.9 mmol, 20%) as a colorless solid.

^{1}H NMR (400 MHz, CD_2Cl_2): *δ* = 7.77 – 7.67 (m, 6H), 7.59 – 7.52 (m, 3H), 7.47 – 7.40 (m, 6H), 3.18 (t, *J* = 11.2 Hz, 2H) ppm.
^{13}C NMR (151 MHz, CD_2Cl_2): *δ* = 133.5 (d, *J* = 3.0 Hz), 131.7 (d, *J* = 11.1 Hz), 129.4 (d, *J* = 13.4 Hz), 128.6 (d, *J* = 109.8 Hz), 18.6 (t, *J* = 48.5 Hz) ppm.
^{31}P NMR (162 MHz, CD_2Cl_2): *δ* = 8.15 (s) ppm.
HRMS for $C_{25}H_{22}N_2O_2P_2S$ (ESI+) $[M+H]^+$ m/z: calc: 477.0950, found: 477.0944.

7 NMR Spectra

2,4,6-Triisopropylbenzenesulfonamide (24)

^1H NMR ($CDCl_3$, 400 MHz)

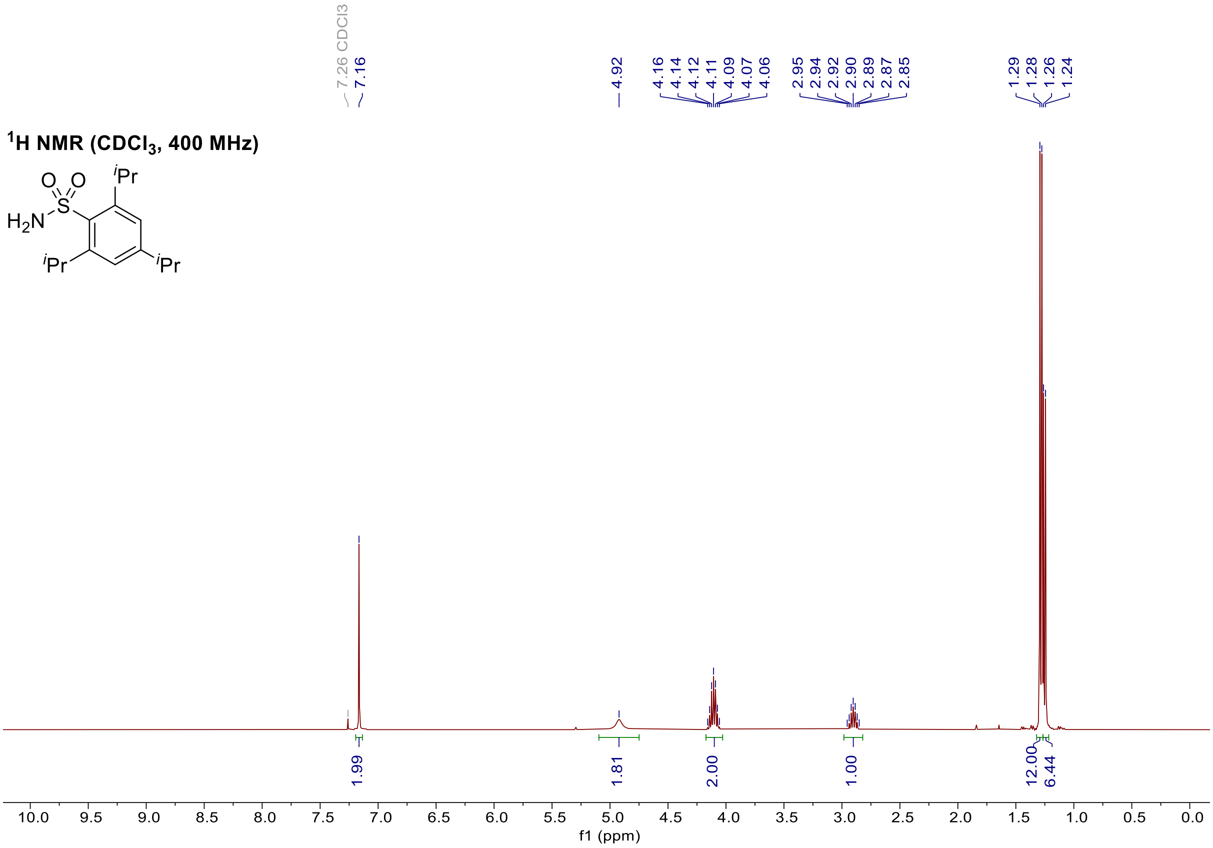

^{13}C NMR ($CDCl_3$, 101 MHz)

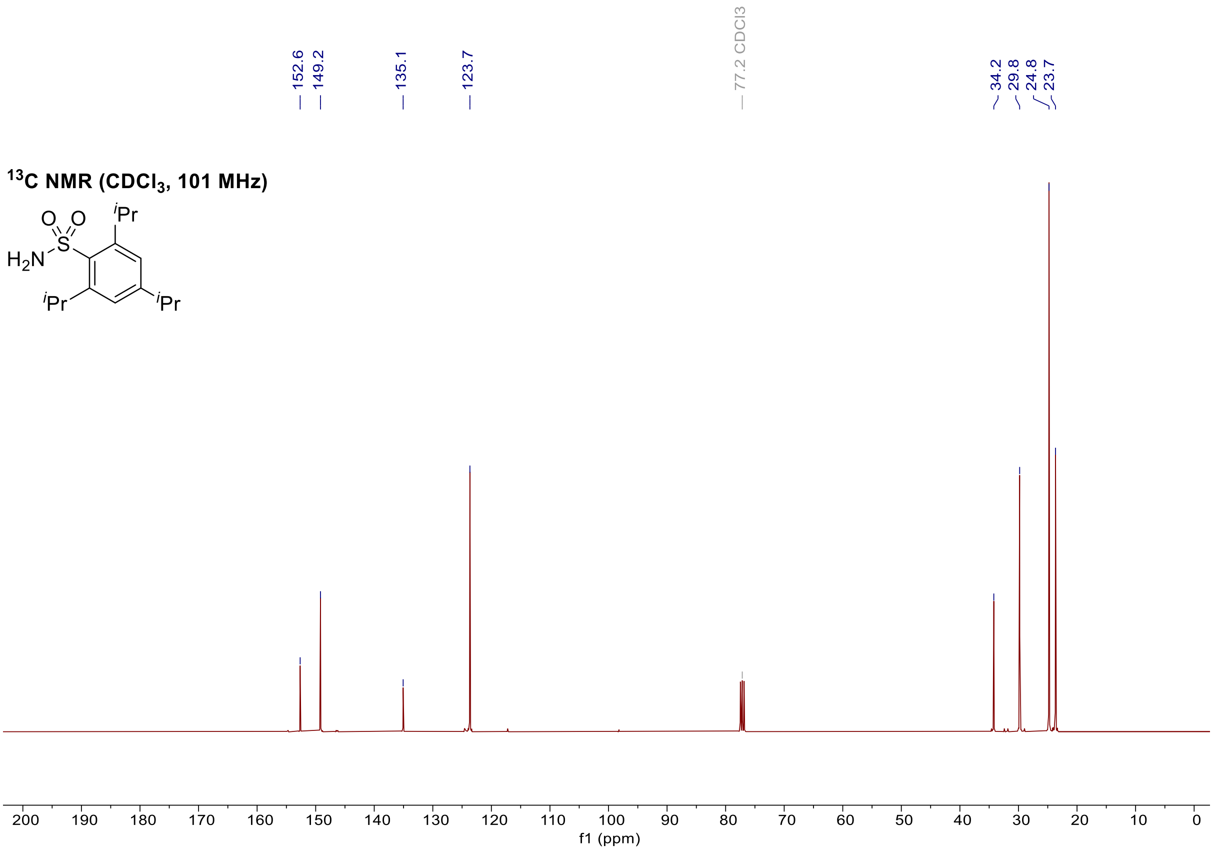

(1*S*)-10-Camphorsulfonamide (25)

^{1}H NMR ($CDCl_3$, 400 MHz)

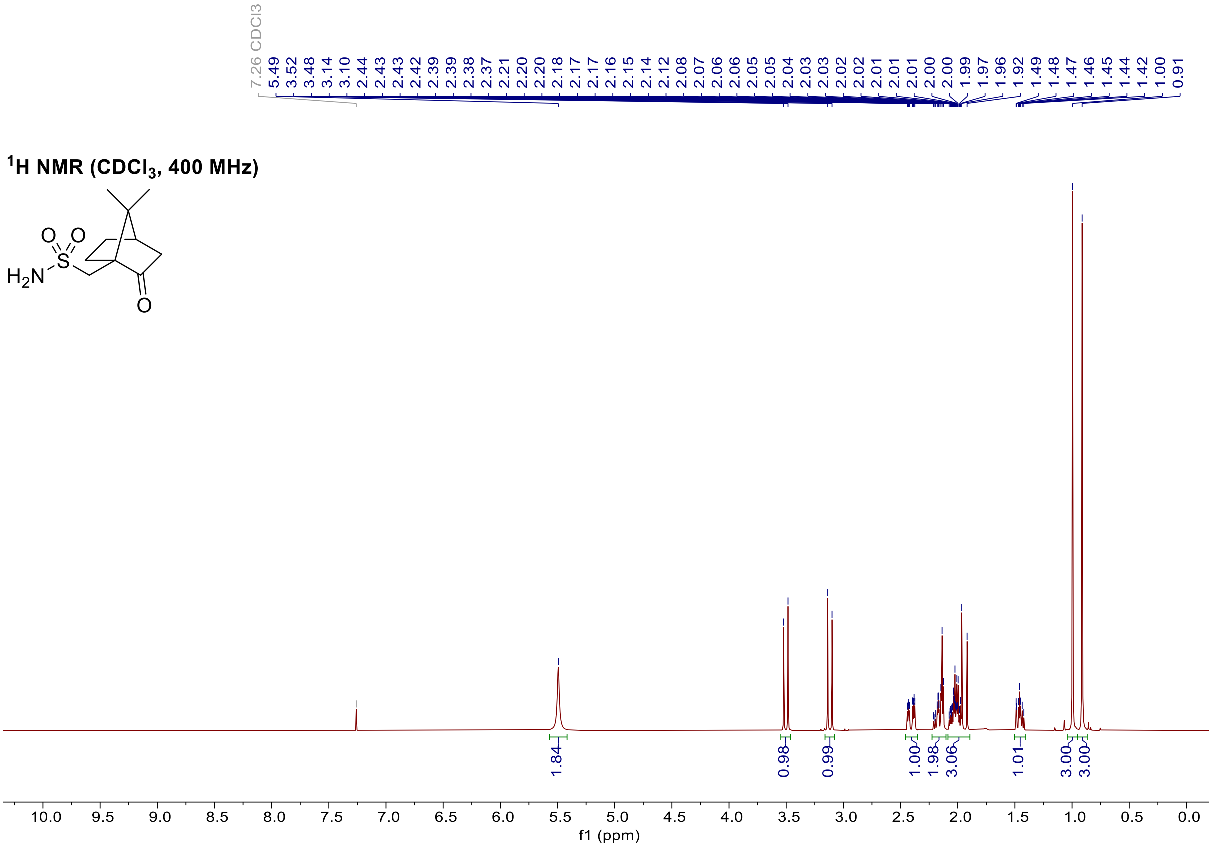

^{13}C NMR ($CDCl_3$, 101 MHz)

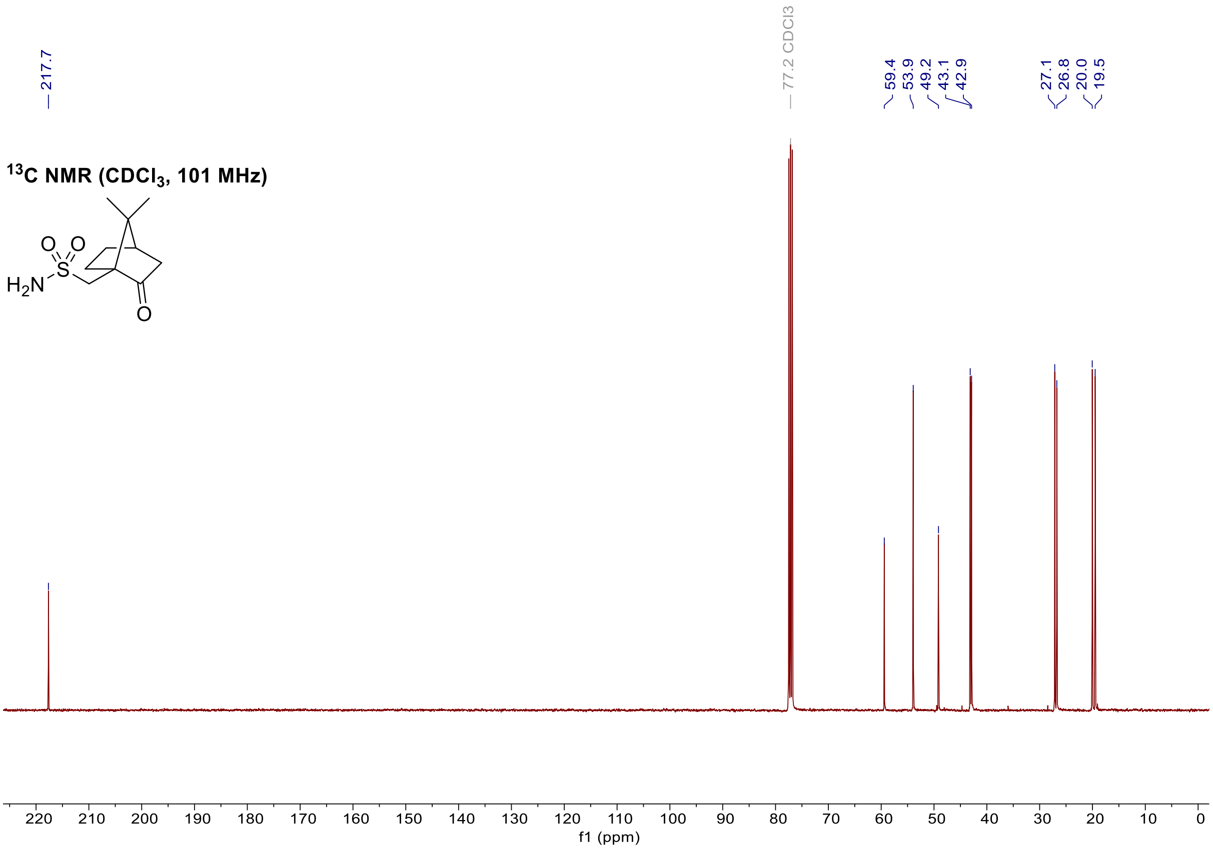

4-Methyl-*N*-(triphenyl-λ^5-phosphanylidene)benzenesulfonamide (3)

^{1}H NMR ($CDCl_3$, 400 MHz)

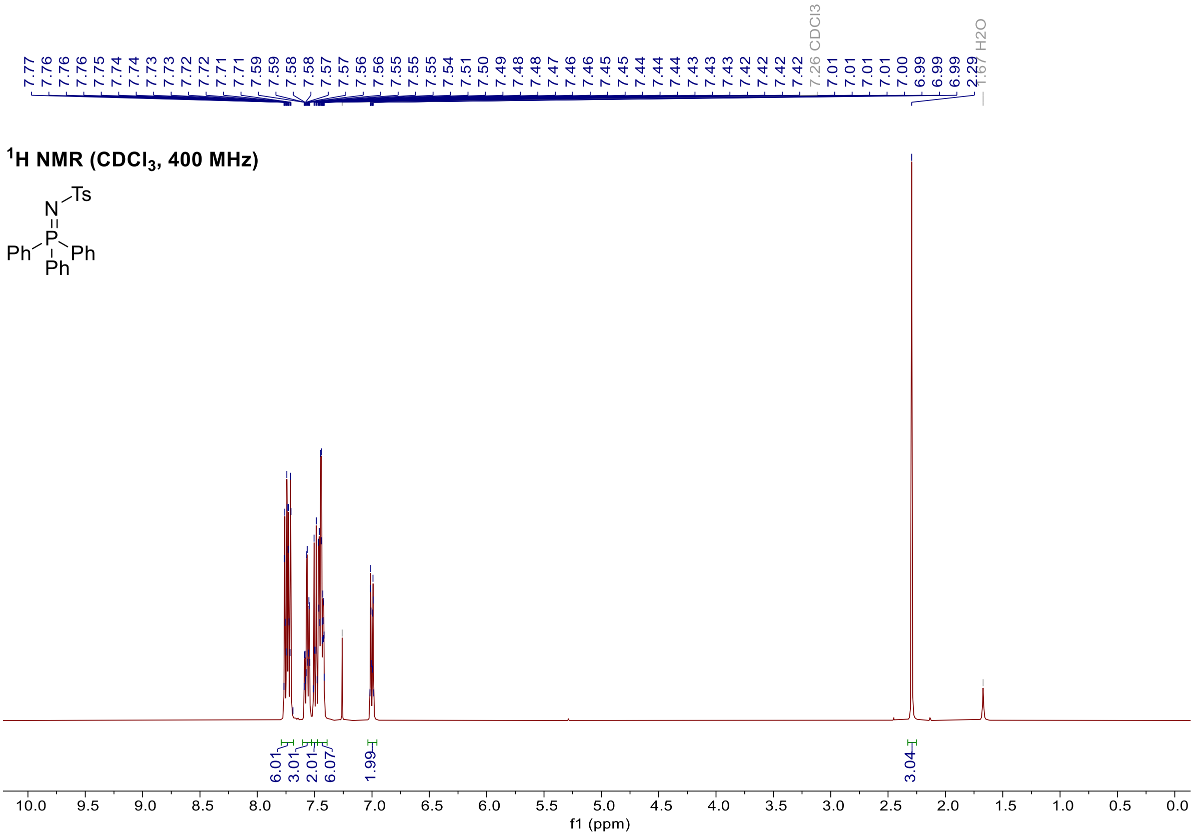

^{13}C NMR ($CDCl_3$, 101 MHz)

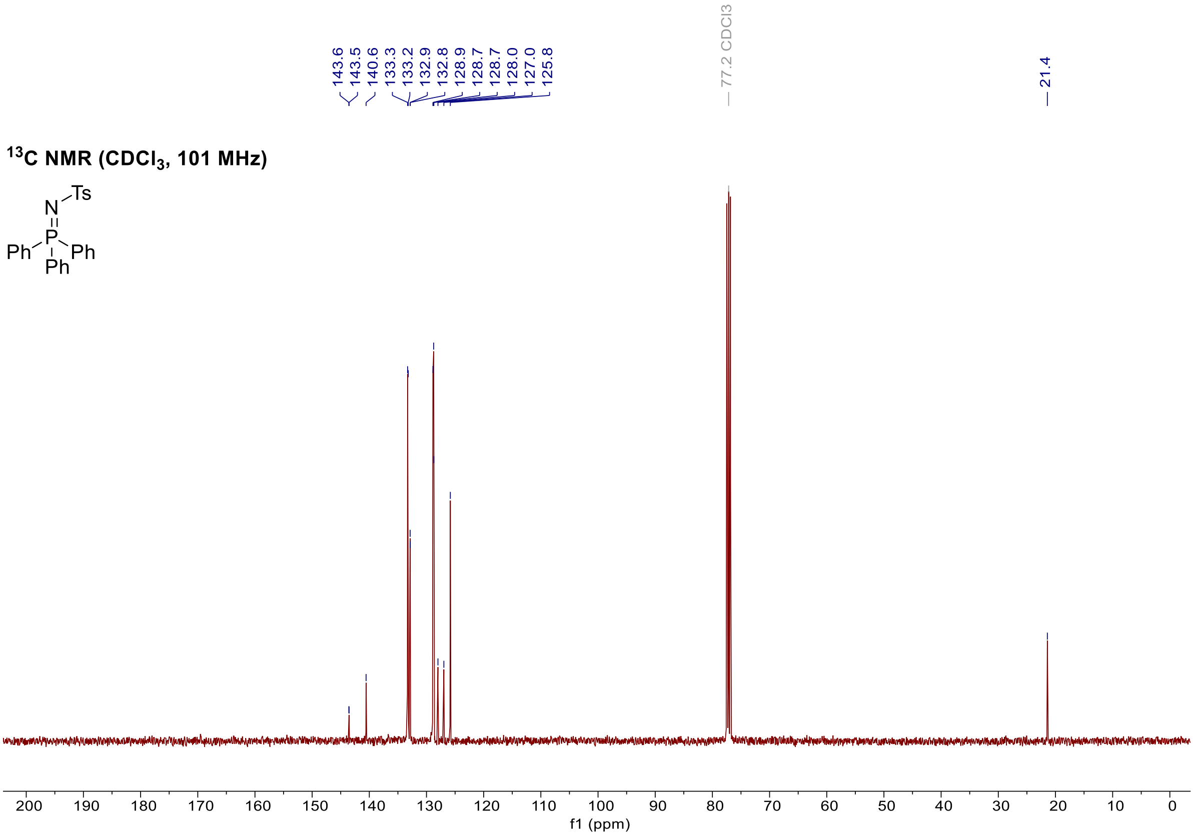

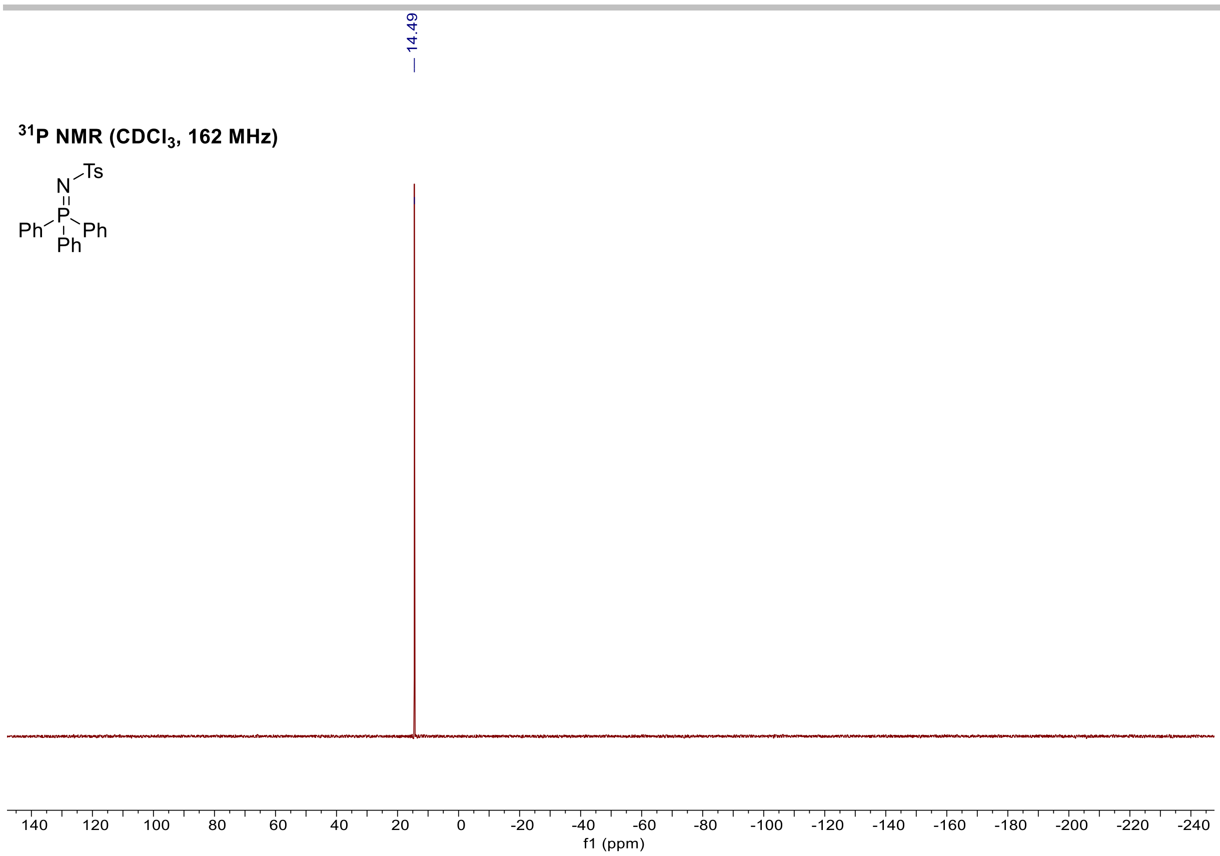

4-Methyl-*N*-(tri-*o*-tolyl-*λ*[5]-phosphanylidene)benzenesulfonamide (4)

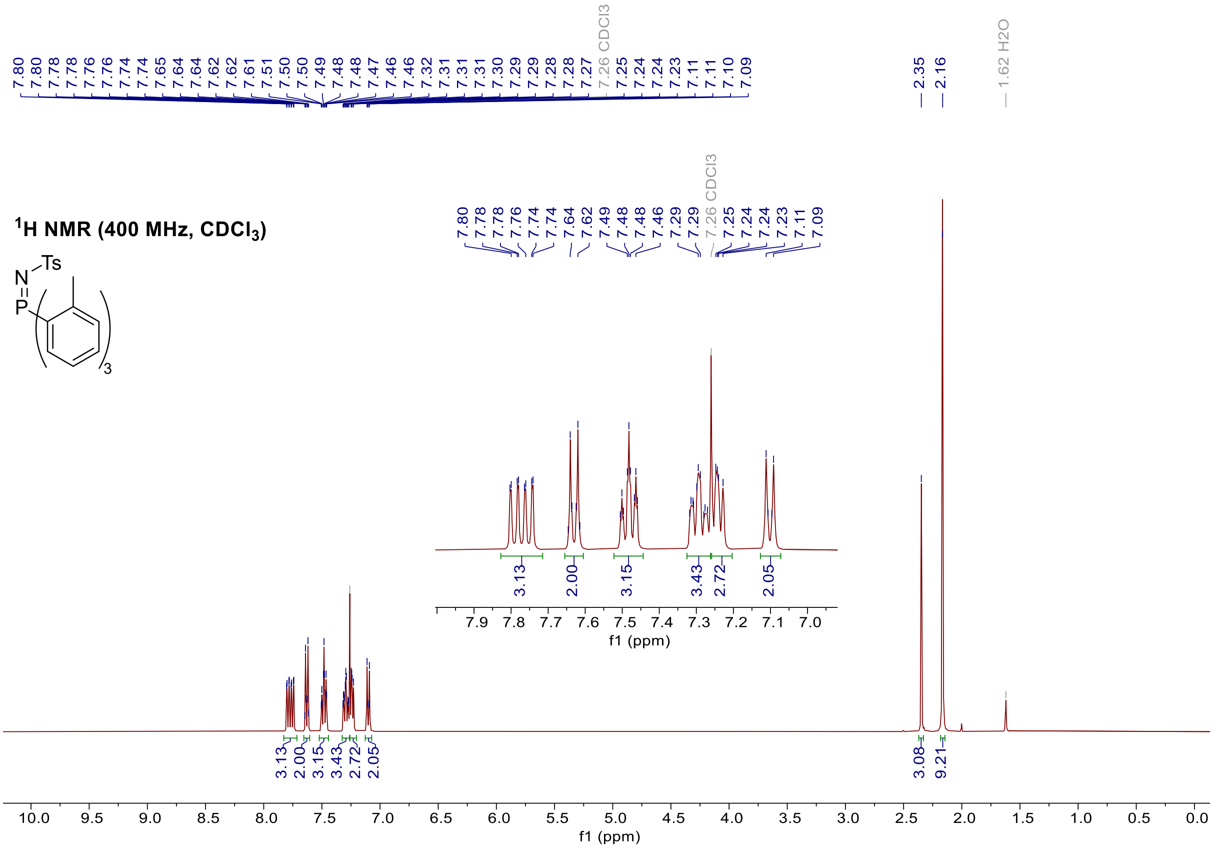

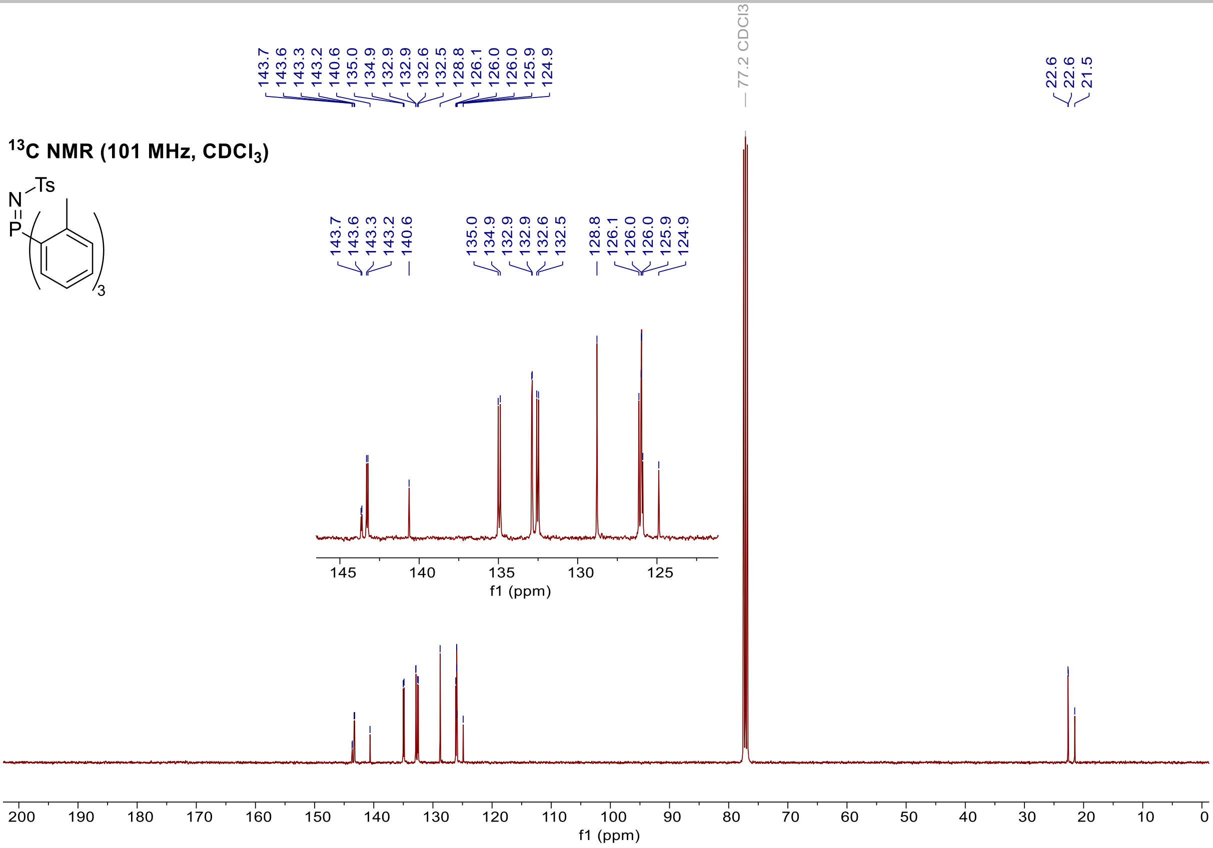
13C NMR (101 MHz, CDCl3)
Ts
N
P
3
143.7
143.6
143.3
143.2
140.6
135.0
134.9
132.9
132.9
132.6
132.5
128.8
126.1
126.0
126.0
125.9
124.9
77.2 CDCl3
22.6
22.6
21.5
145 140 135 130 125
f1 (ppm)
200 190 180 170 160 150 140 130 120 110 100 90 80 70 60 50 40 30 20 10 0
f1 (ppm)

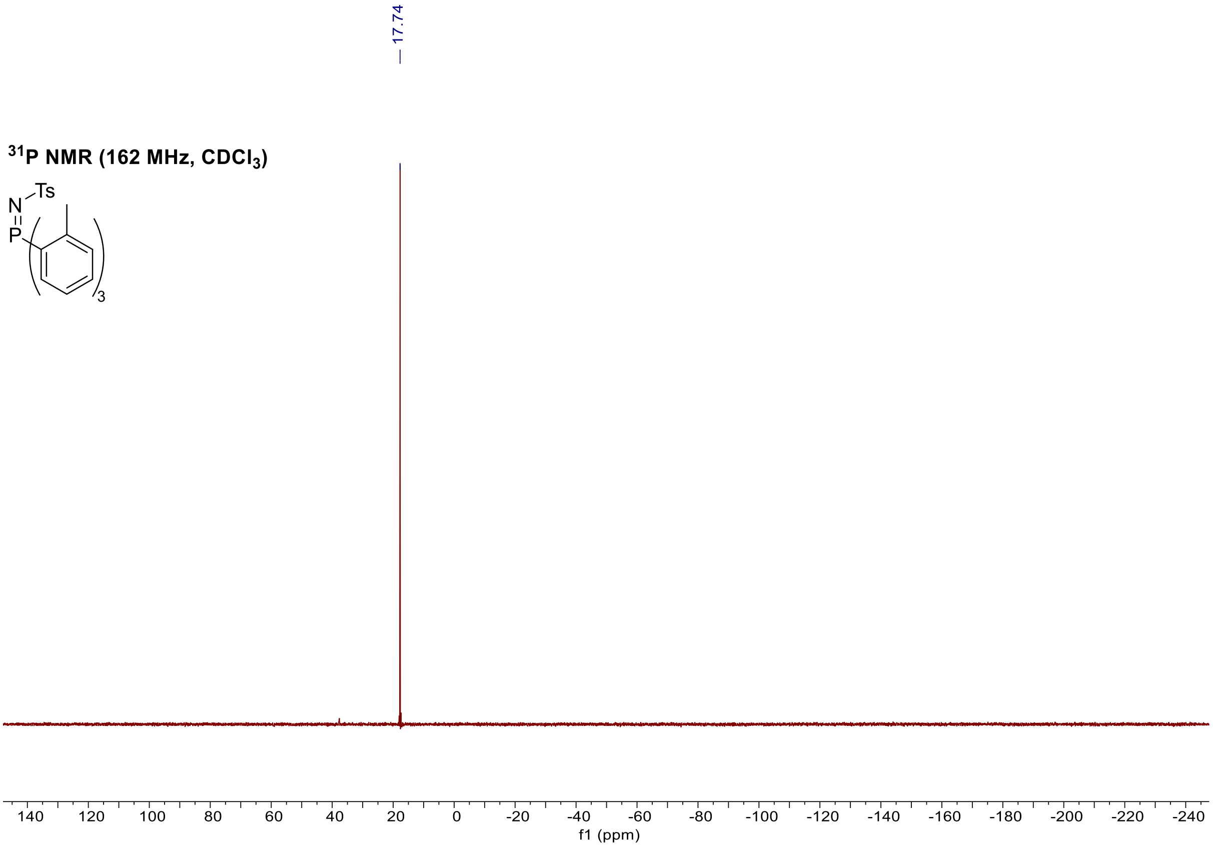
17.74
31P NMR (162 MHz, CDCl3)
Ts
N
P
3
140 120 100 80 60 40 20 0 -20 -40 -60 -80 -100 -120 -140 -160 -180 -200 -220 -240
f1 (ppm)

4-Methyl-*N*-(tris(4-fluorophenyl)-*λ*[5]-phosphanylidene)benzenesulfonamide (5)

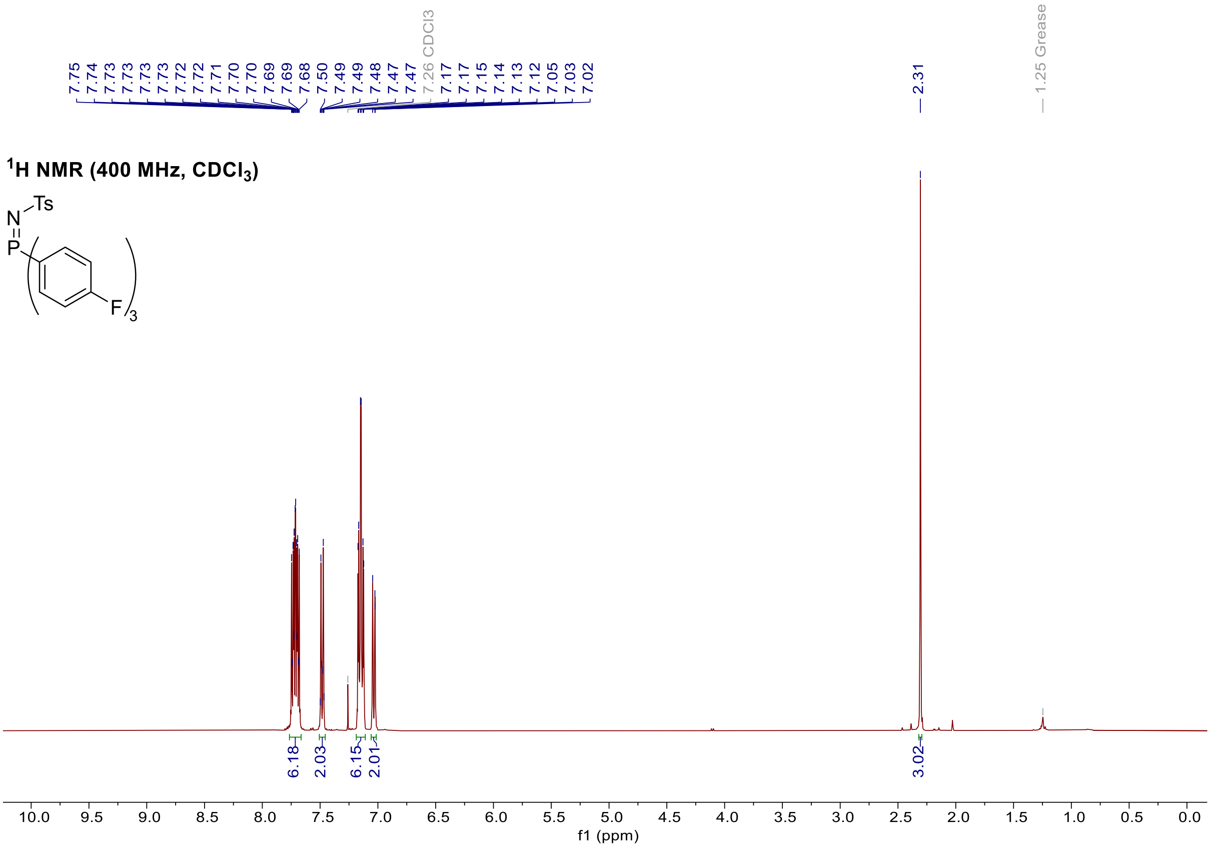

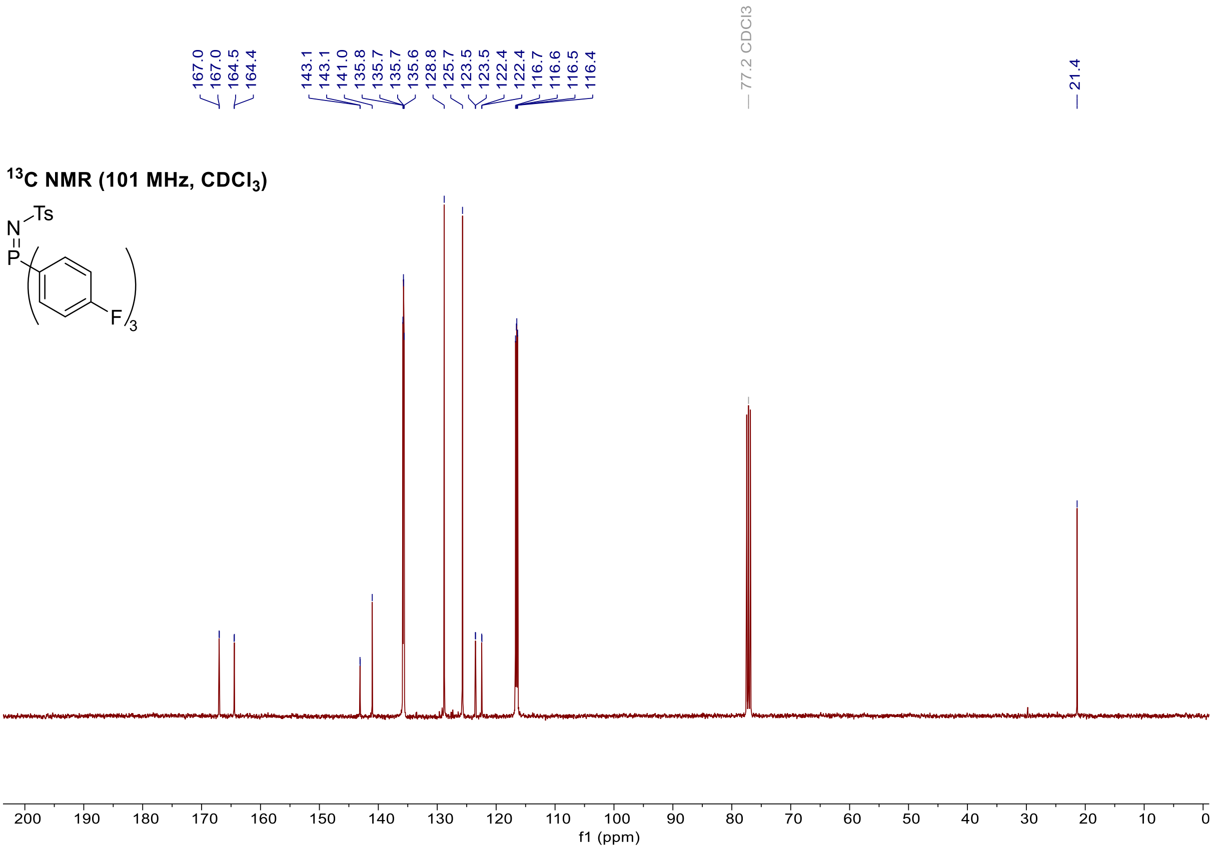

^{31}P NMR (162 MHz, $CDCl_3$)

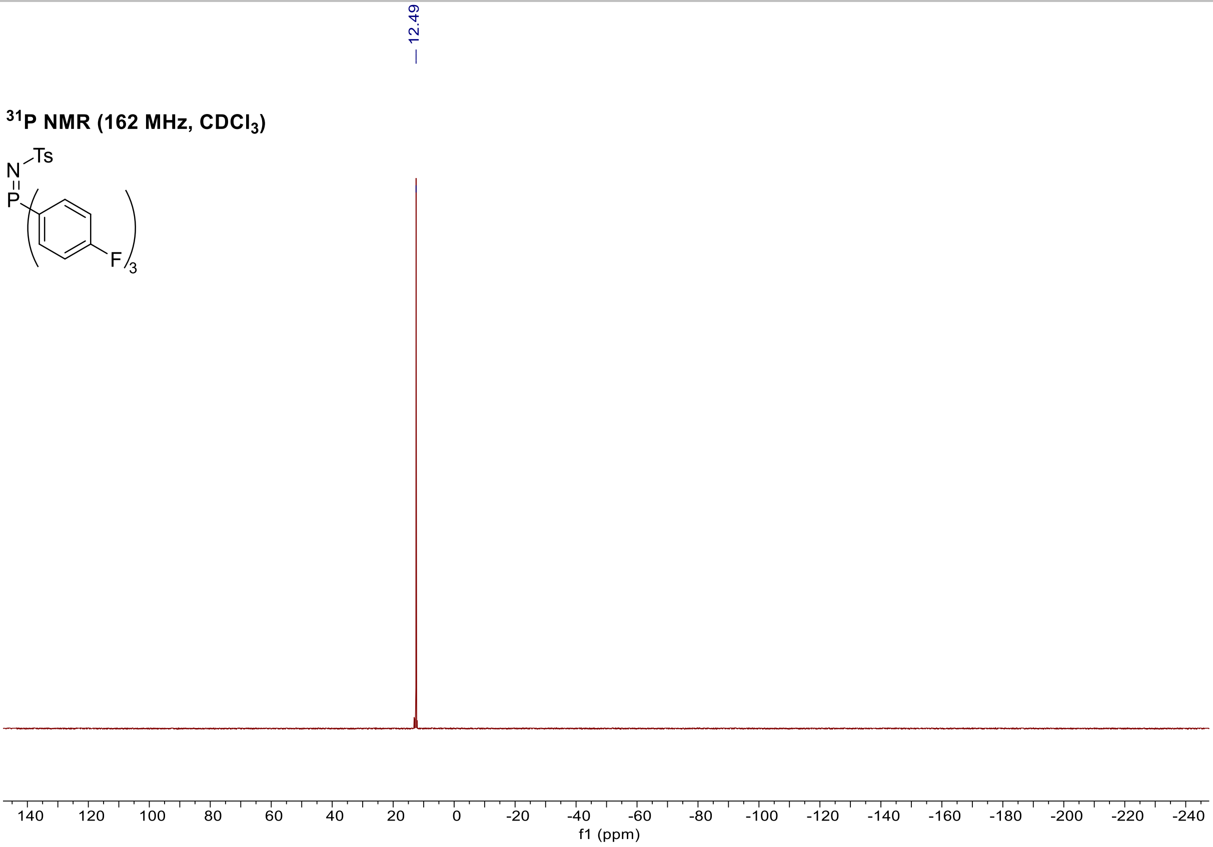

^{19}F NMR (376 MHz, $CDCl_3$)

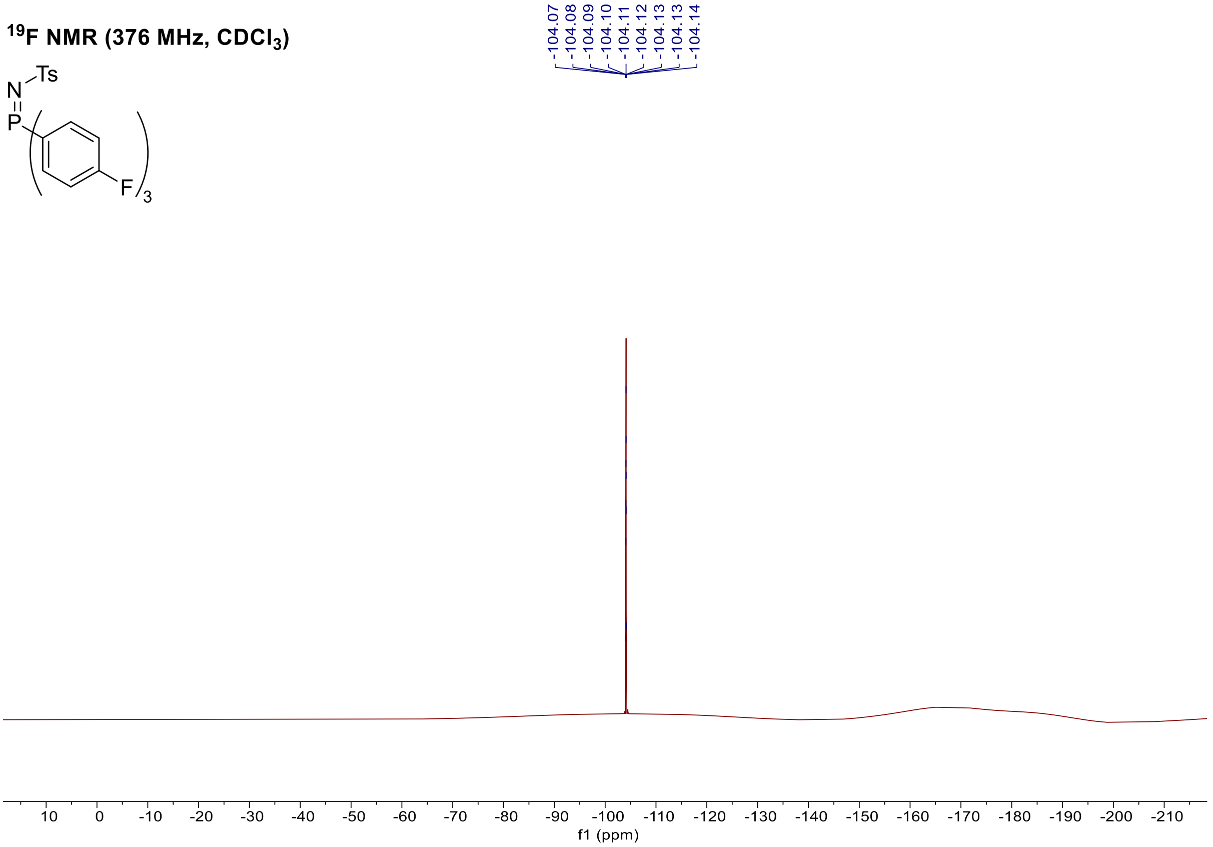

4-Methyl-*N*-(tri(furan-2-yl)-λ[5]-phosphanylidene)benzenesulfonamide (6)

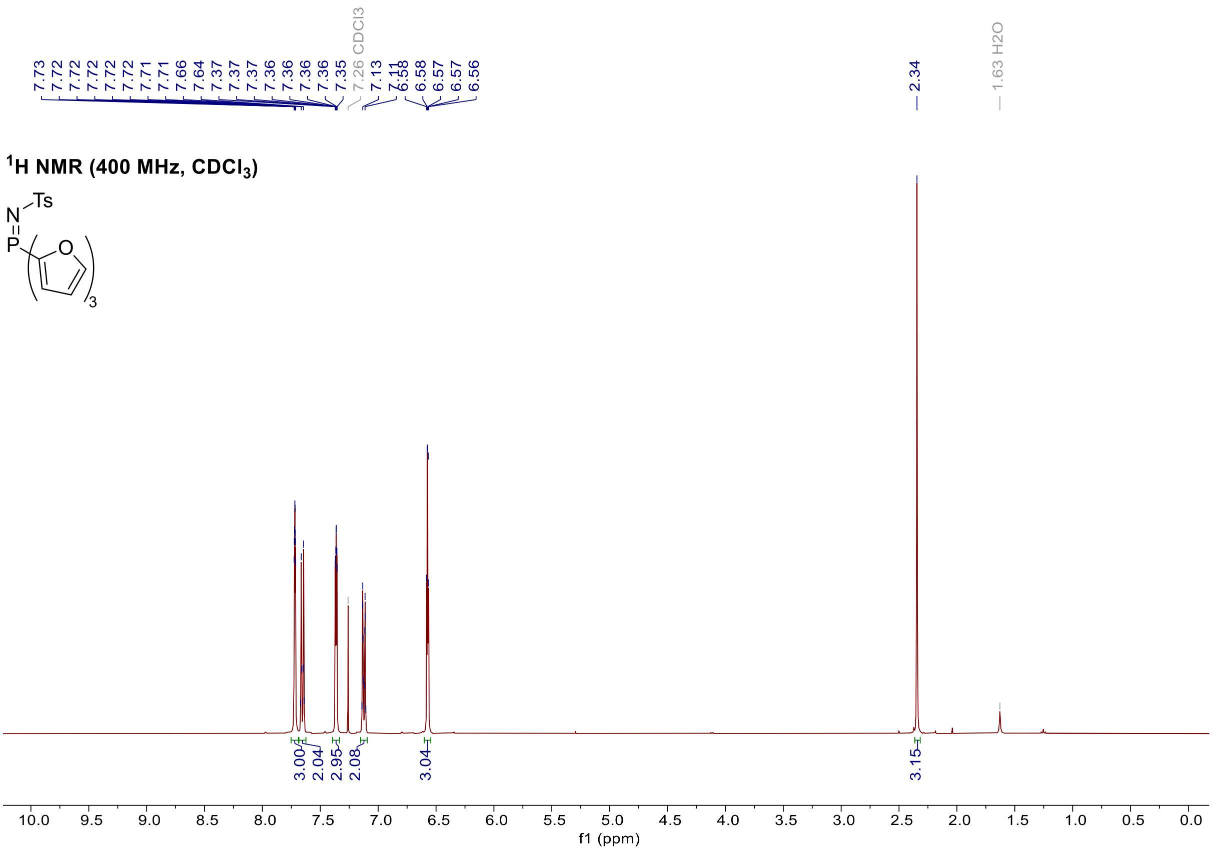

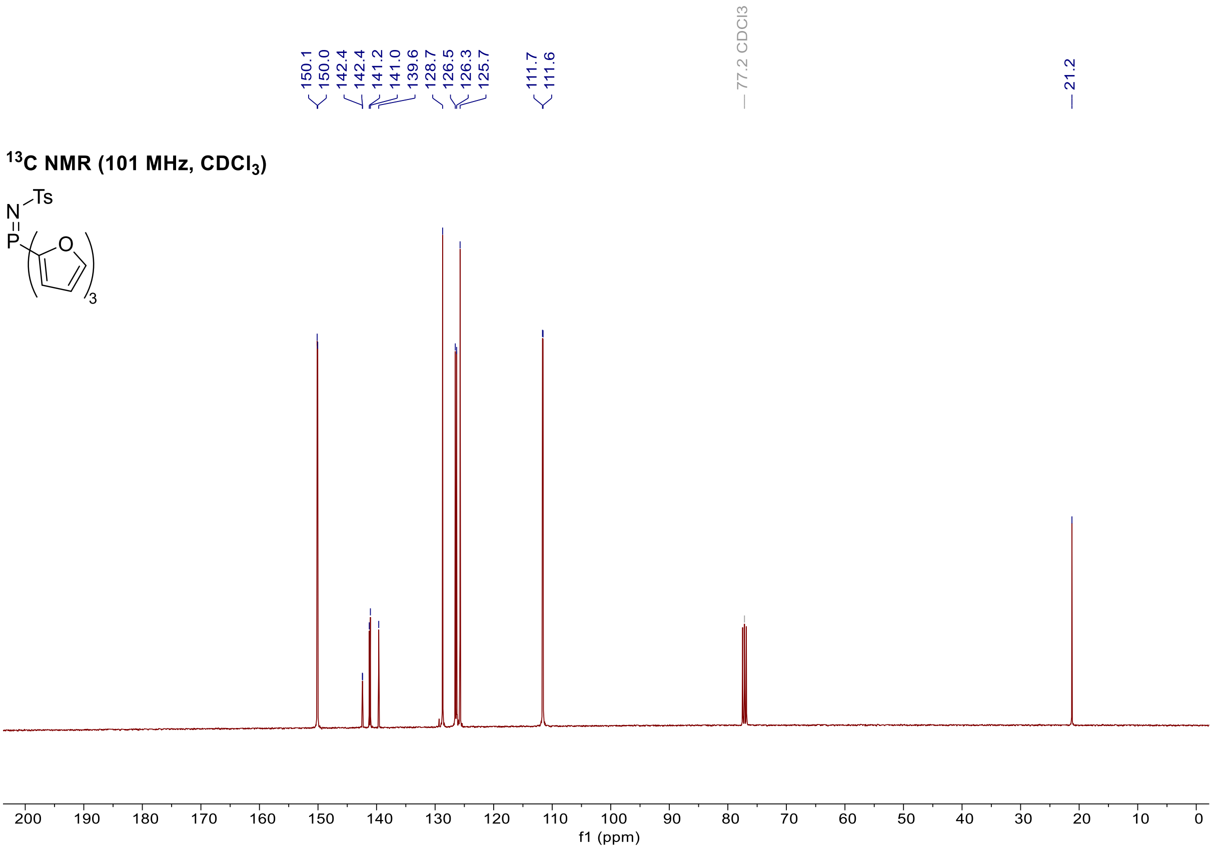

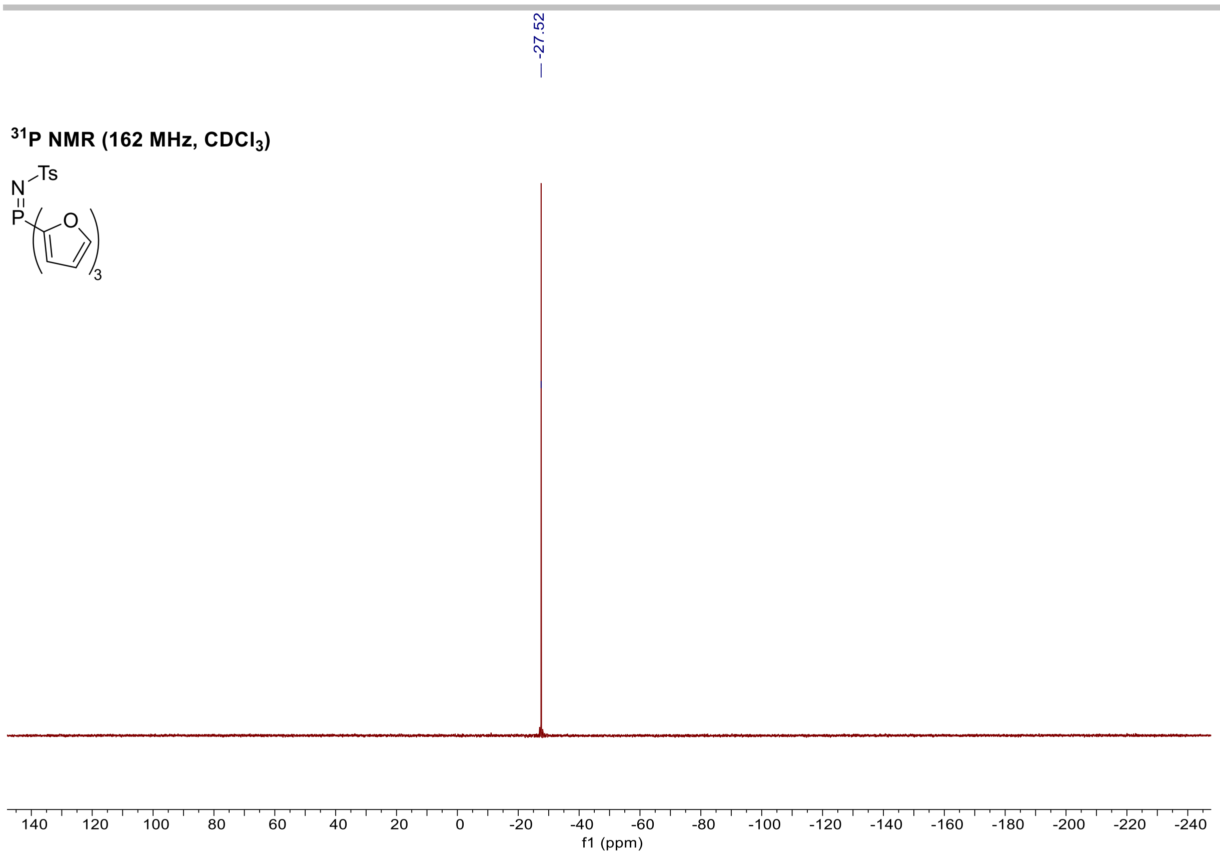

4-Methyl-*N*-(tributyl-λ^5-phosphanylidene)benzenesulfonamide (7)

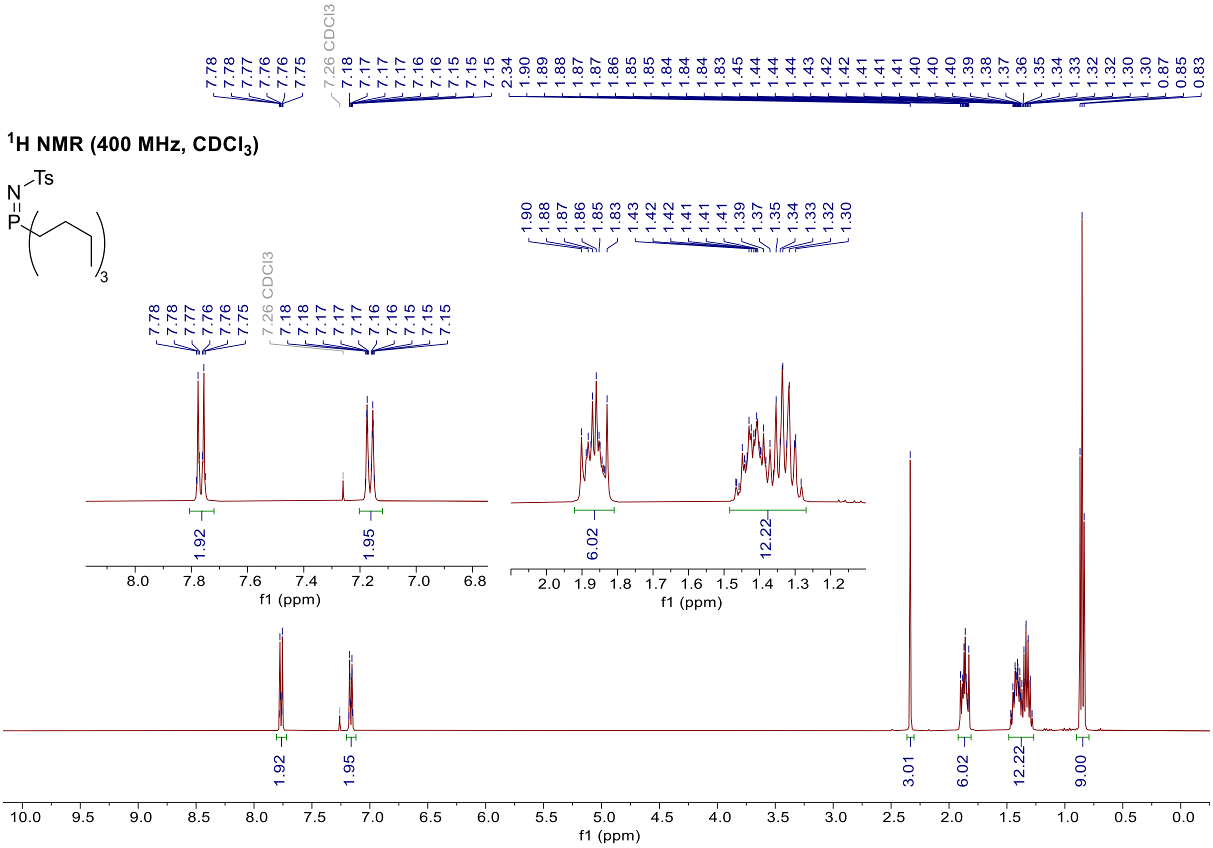

^{13}C NMR (101 MHz, $CDCl_3$)

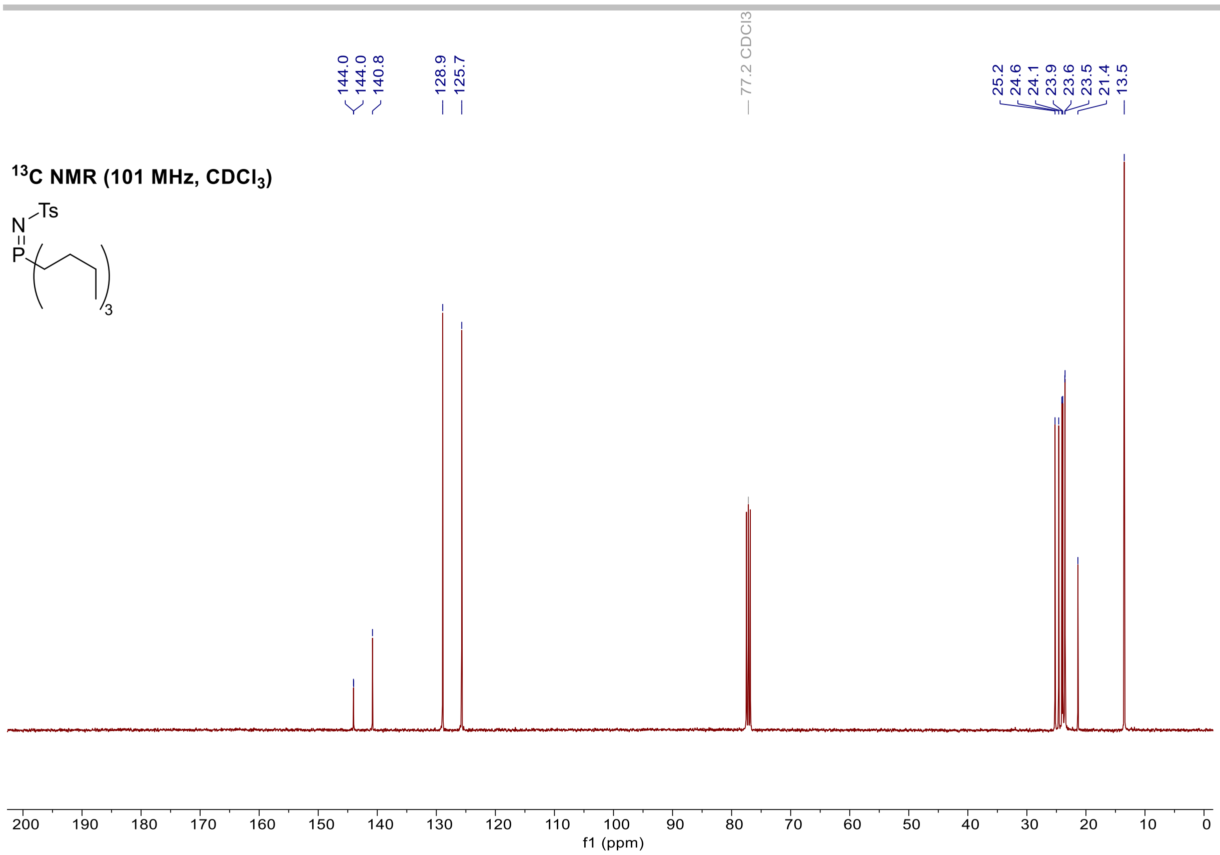

^{31}P NMR (162 MHz, $CDCl_3$)

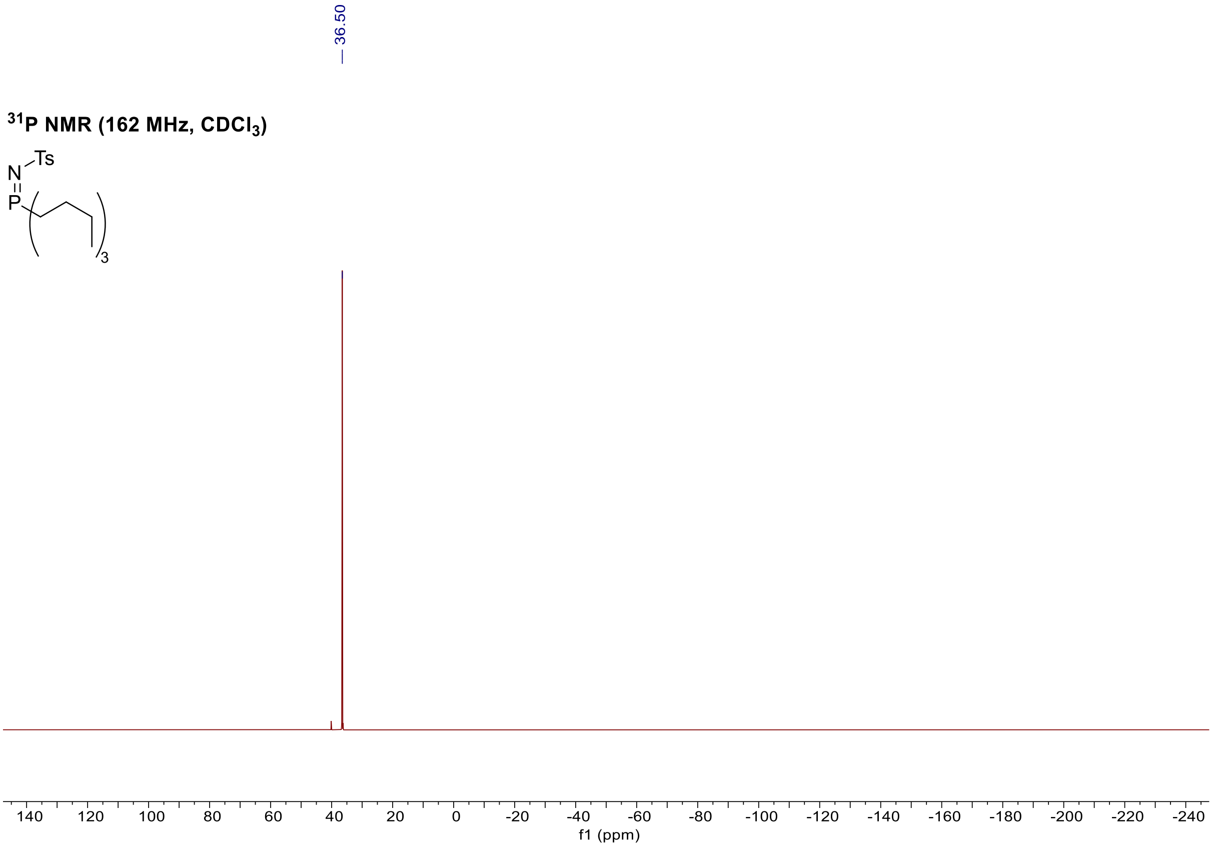

4-Methyl-*N*-(tricyclohexyl-λ^5-phosphanylidene)benzenesulfonamide (8)

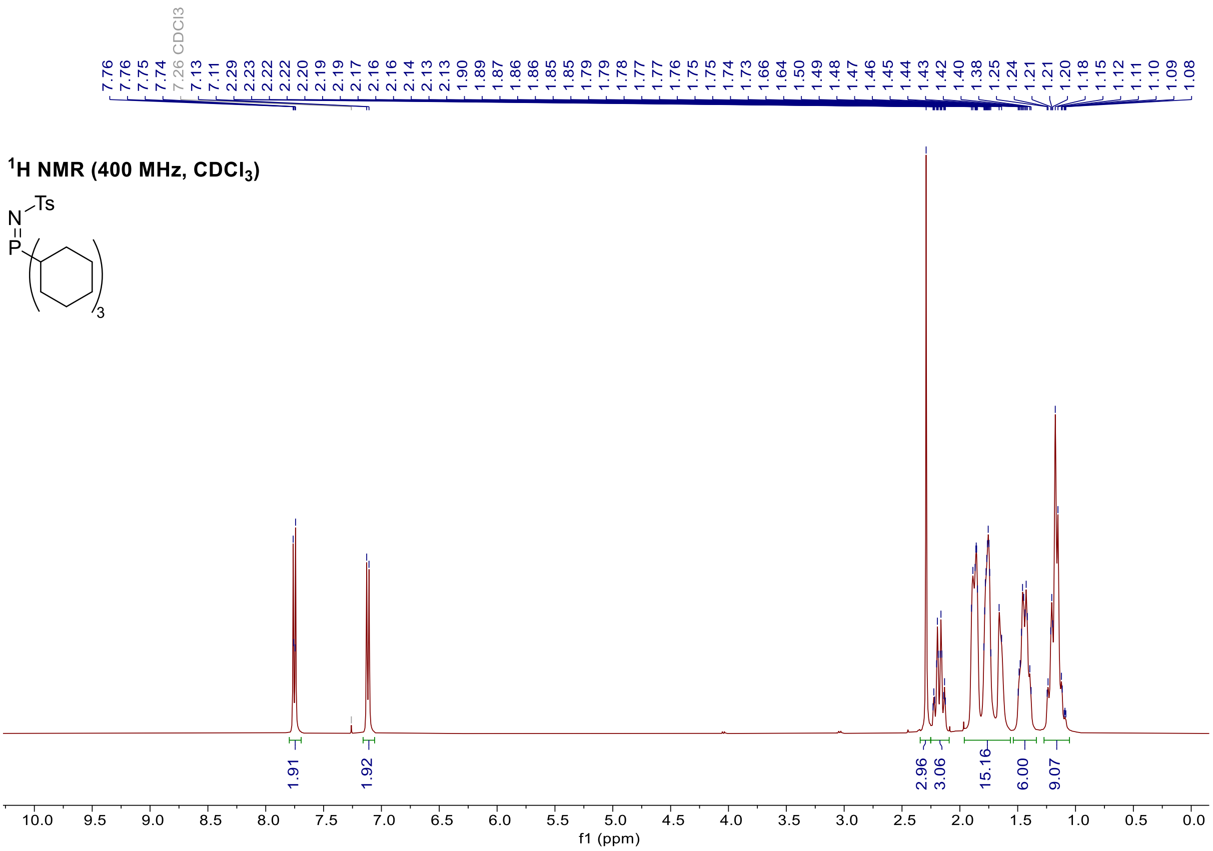

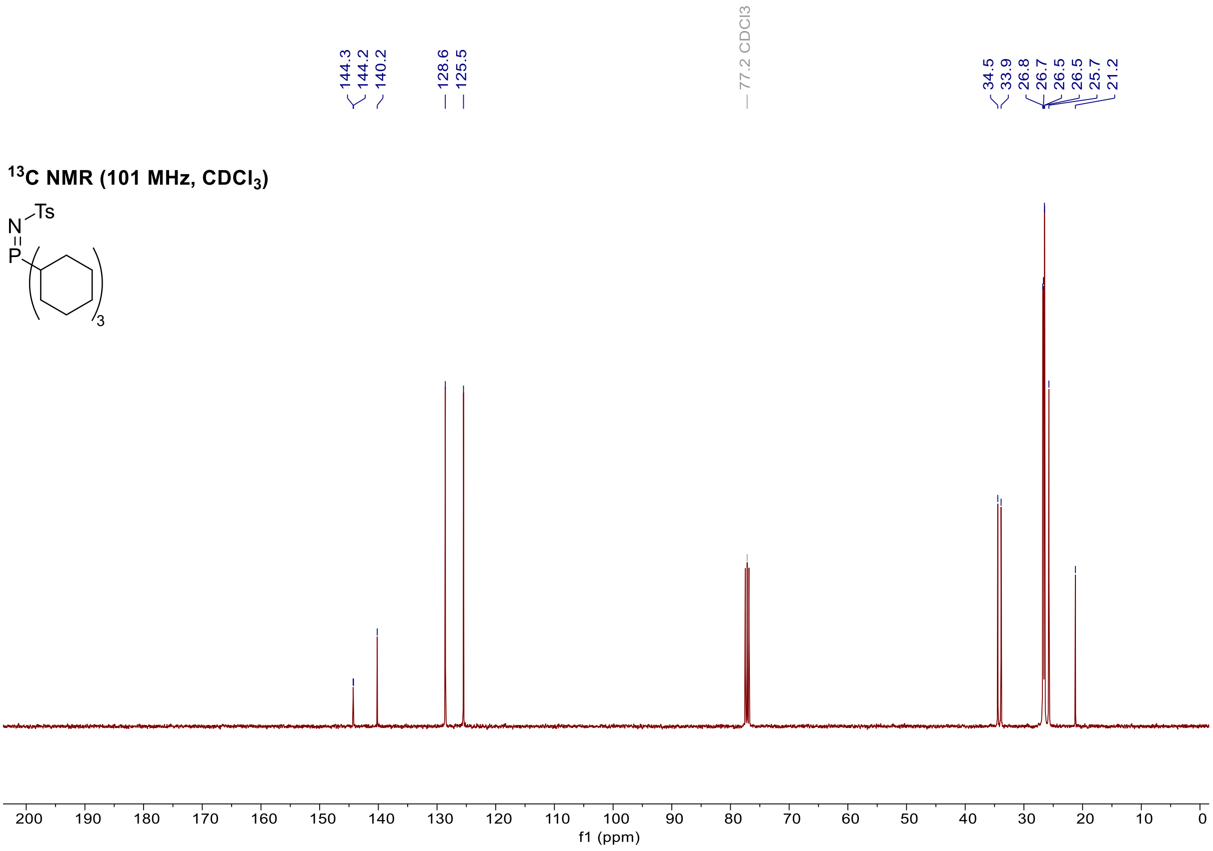

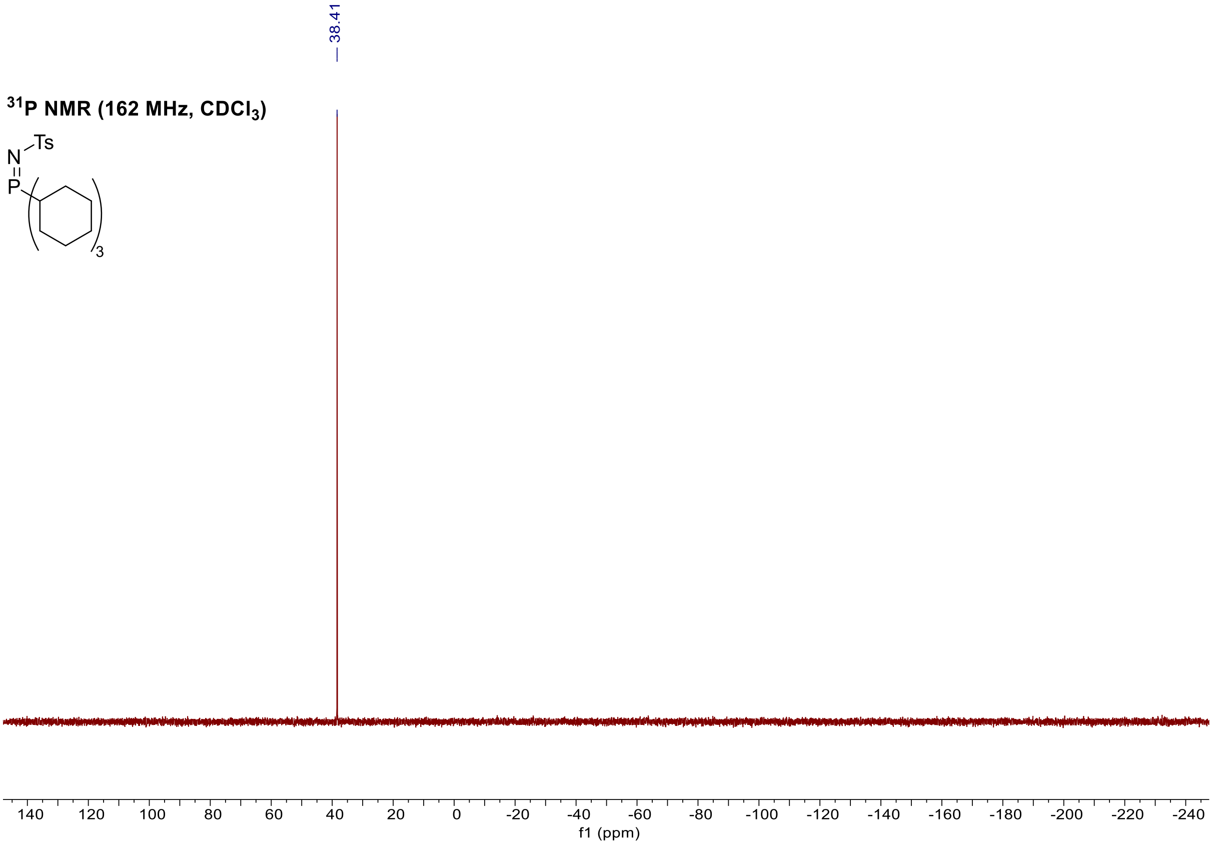

4-Methyl-*N*-(tris(2-cyanoethyl)-λ^5-phosphanylidene)benzenesulfonamide (10)

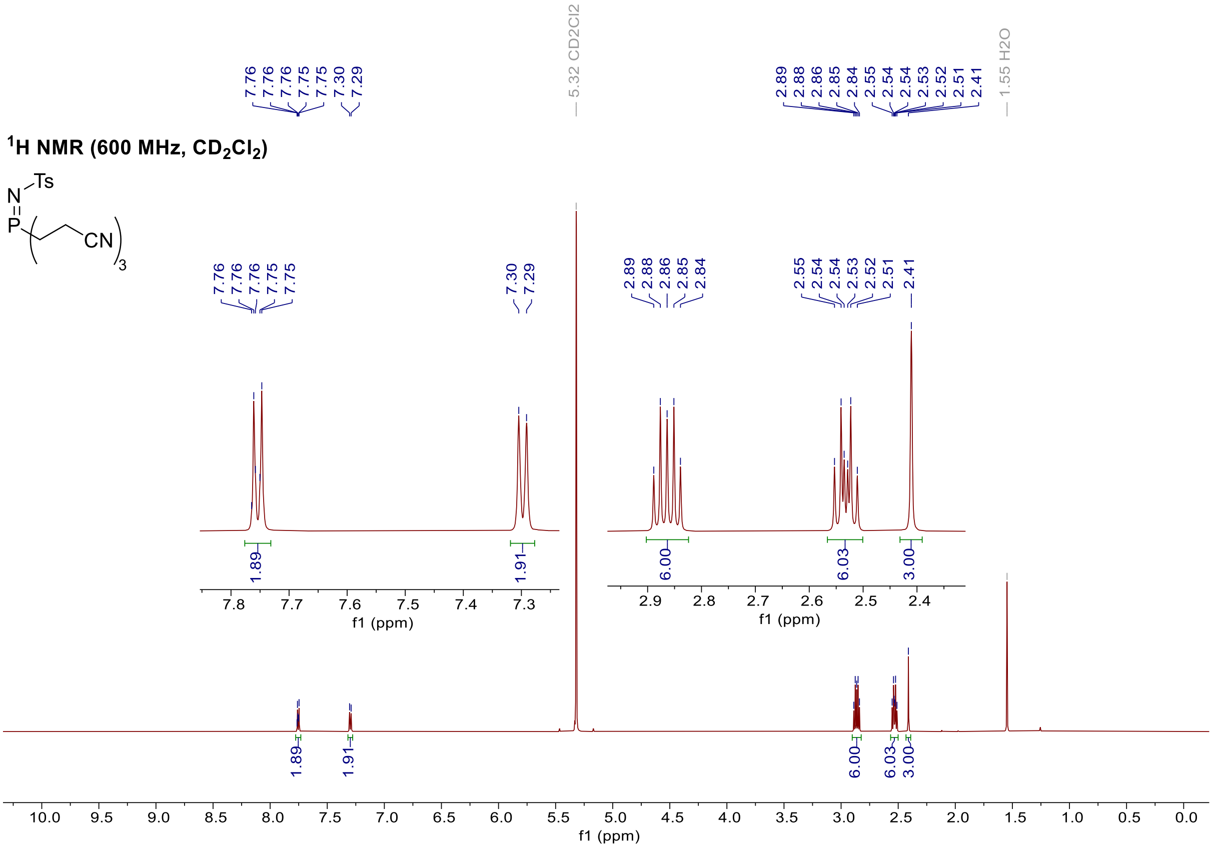

^{13}C NMR (151 MHz, CD_2Cl_2)

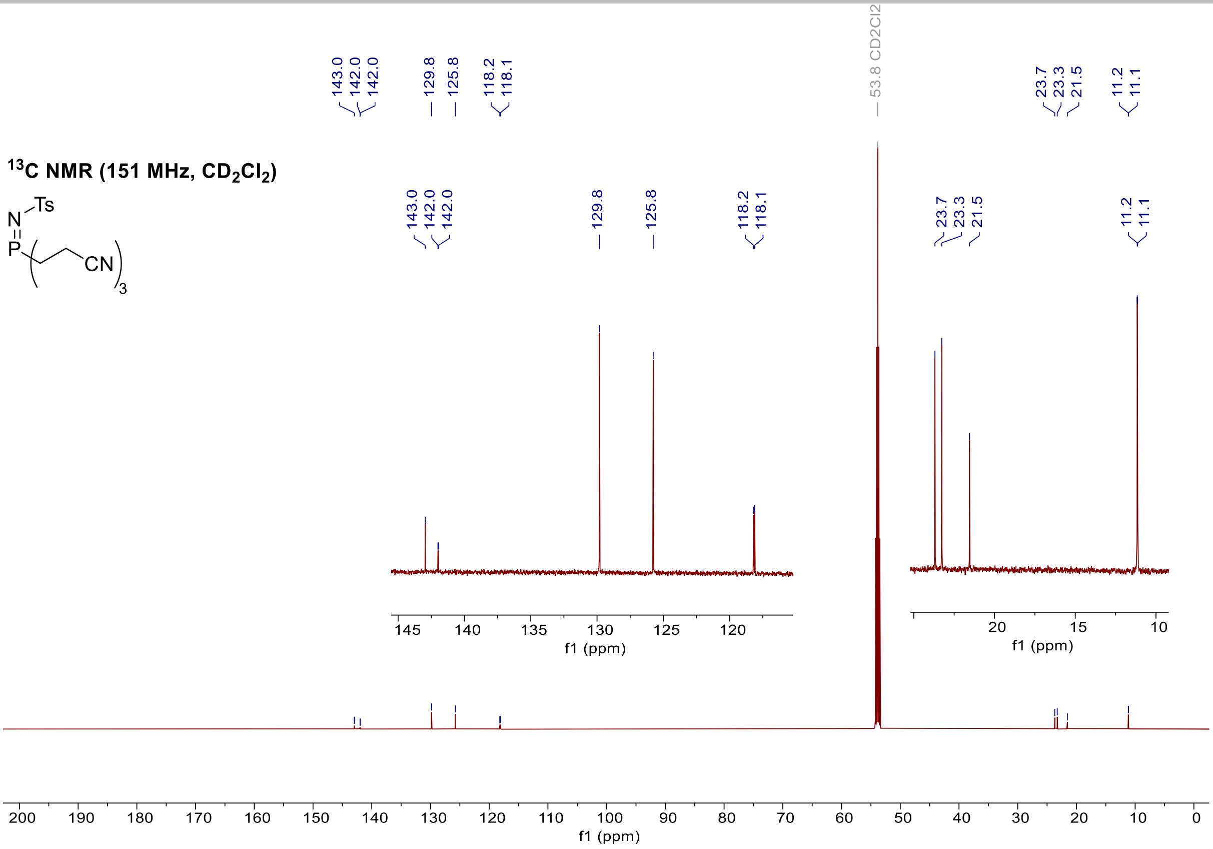

^{31}P NMR (162 MHz, CD_2Cl_2)

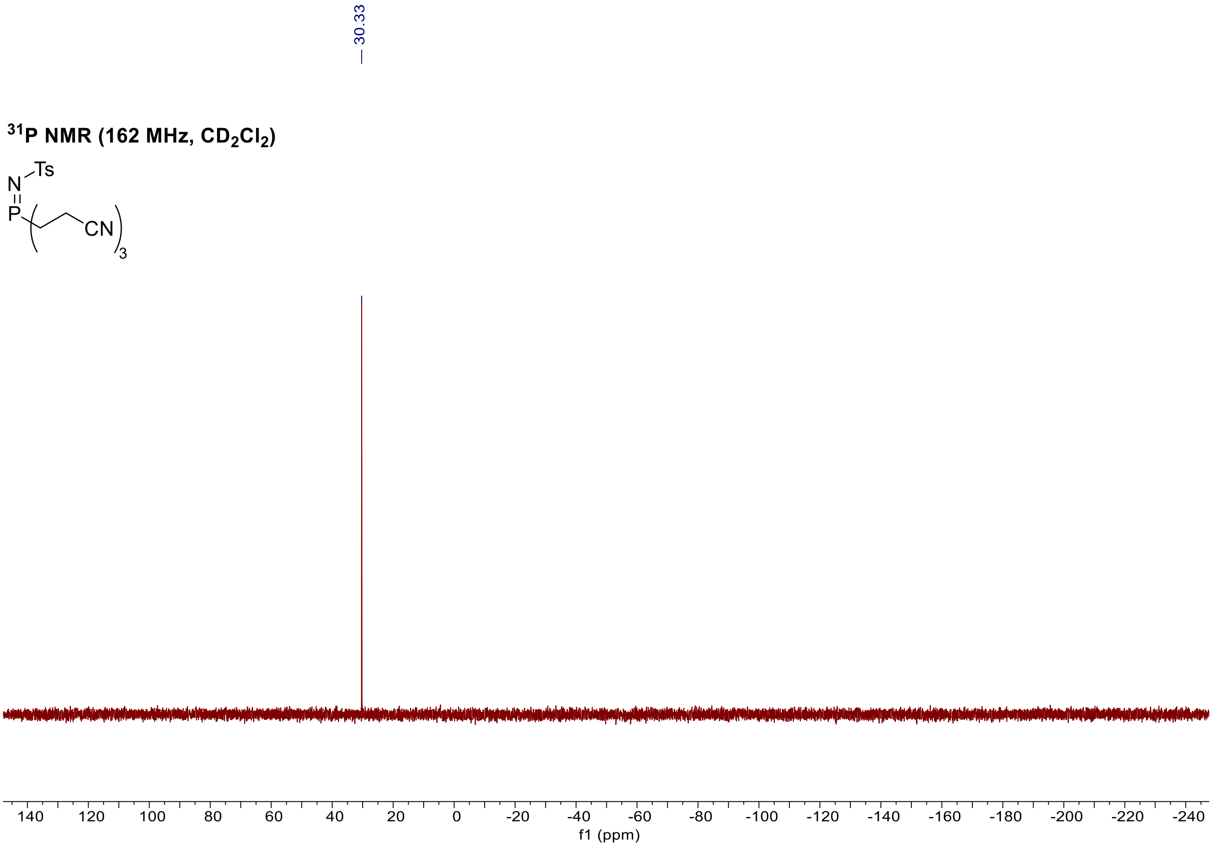

N,N'-(Ethane-1,2-diylbis(diphenyl-λ⁵-phosphanylylidene))bis(4-methylbenzenesulfonamide) (11)

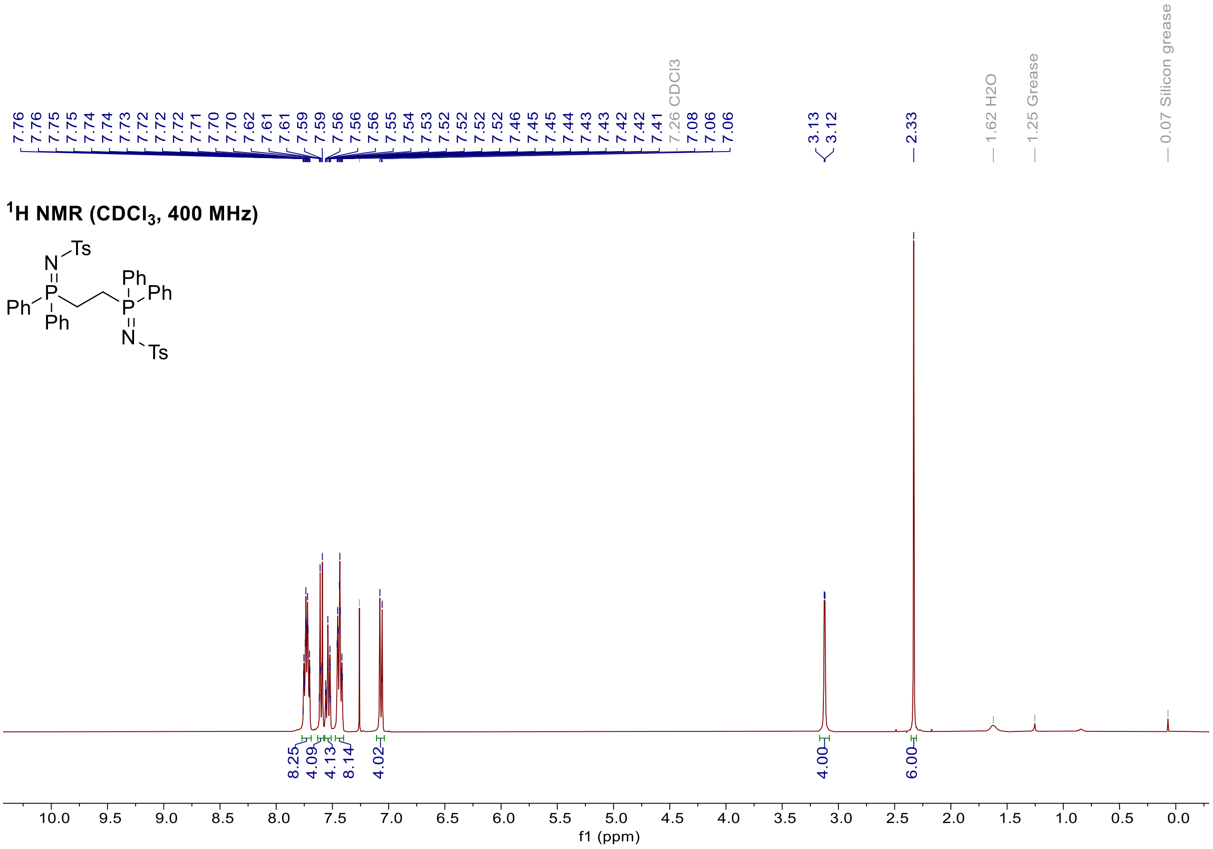

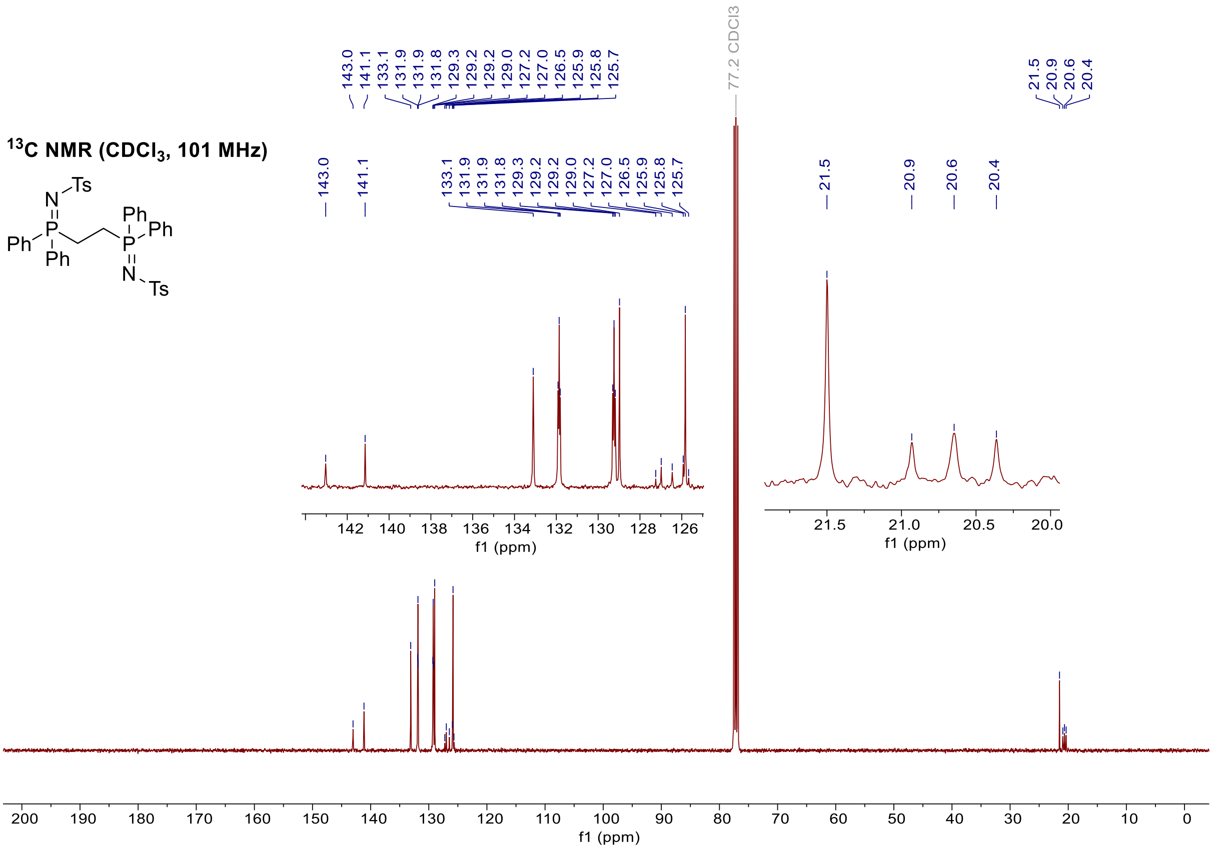

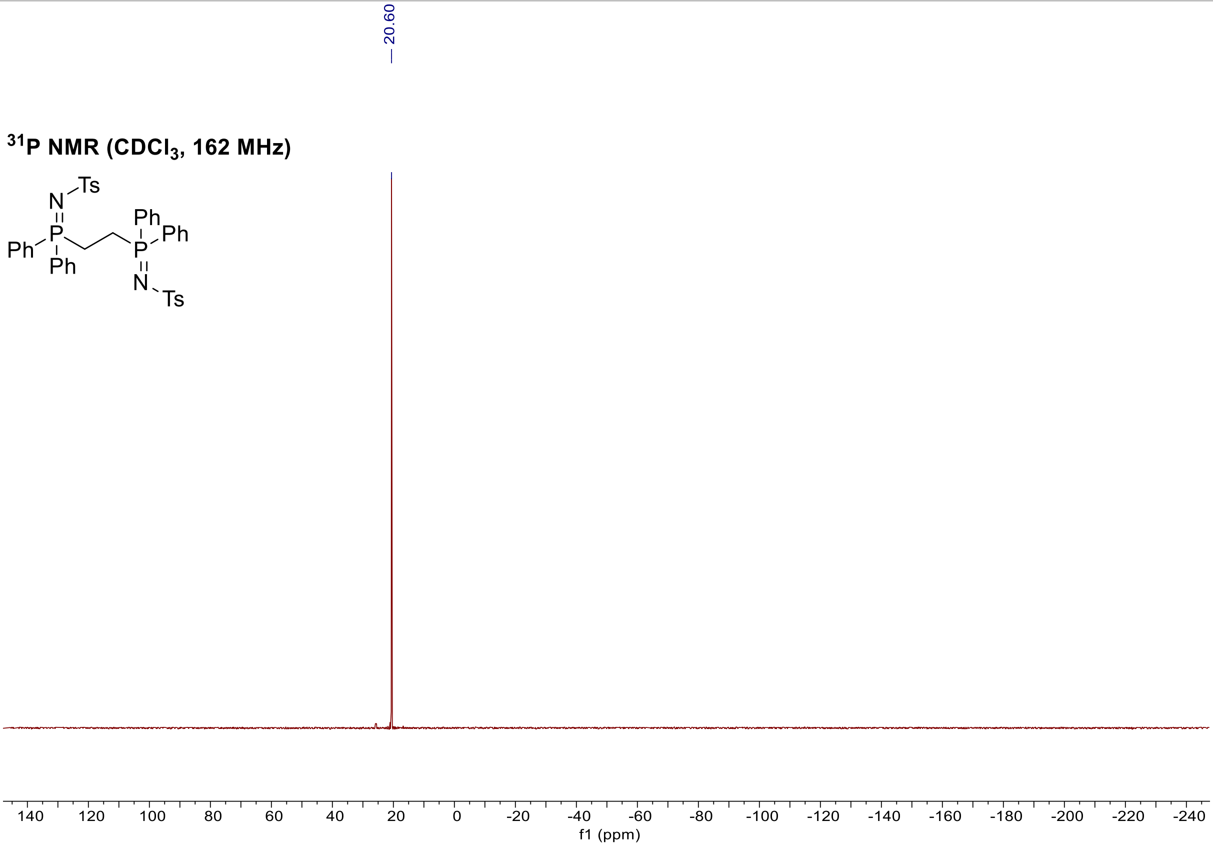

N-(Ethyldiphenyl-λ^5-phosphanylidene)-4-methylbenzenesulfonamide (12)

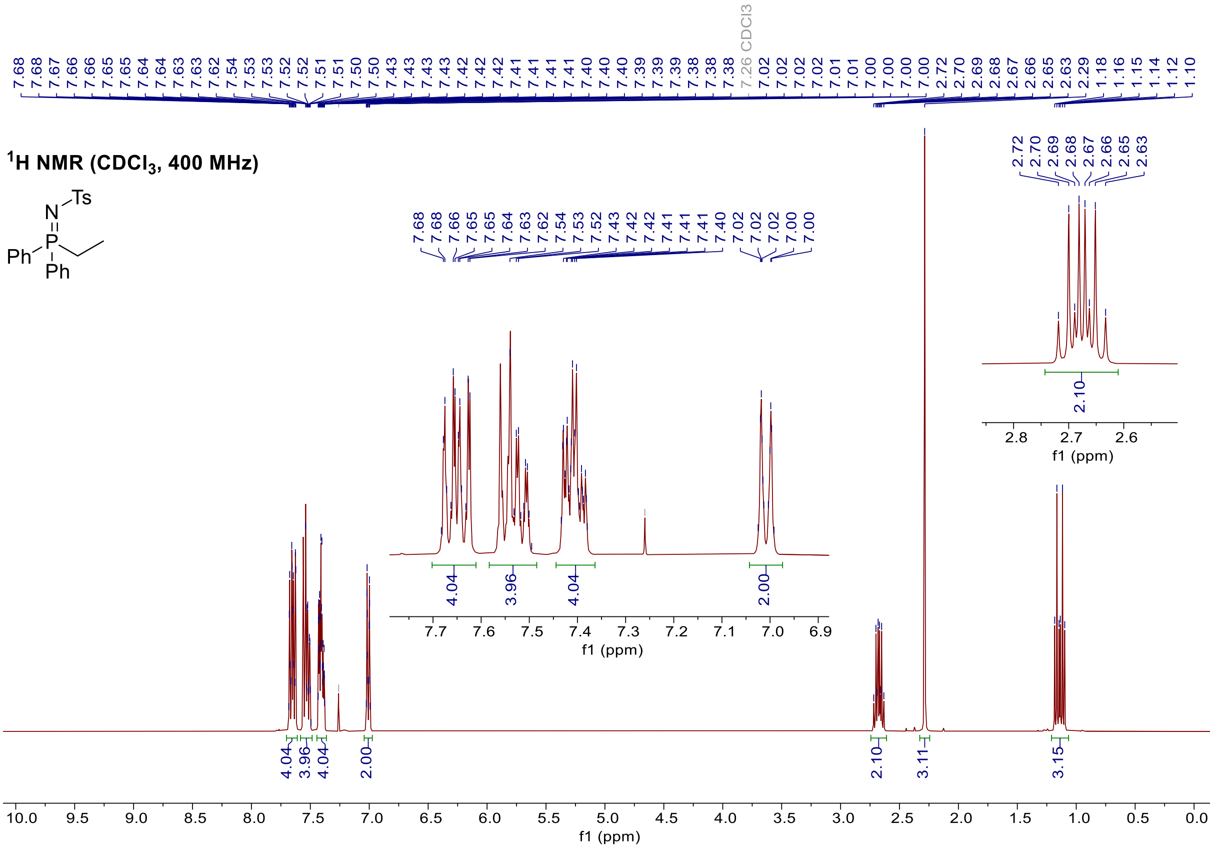

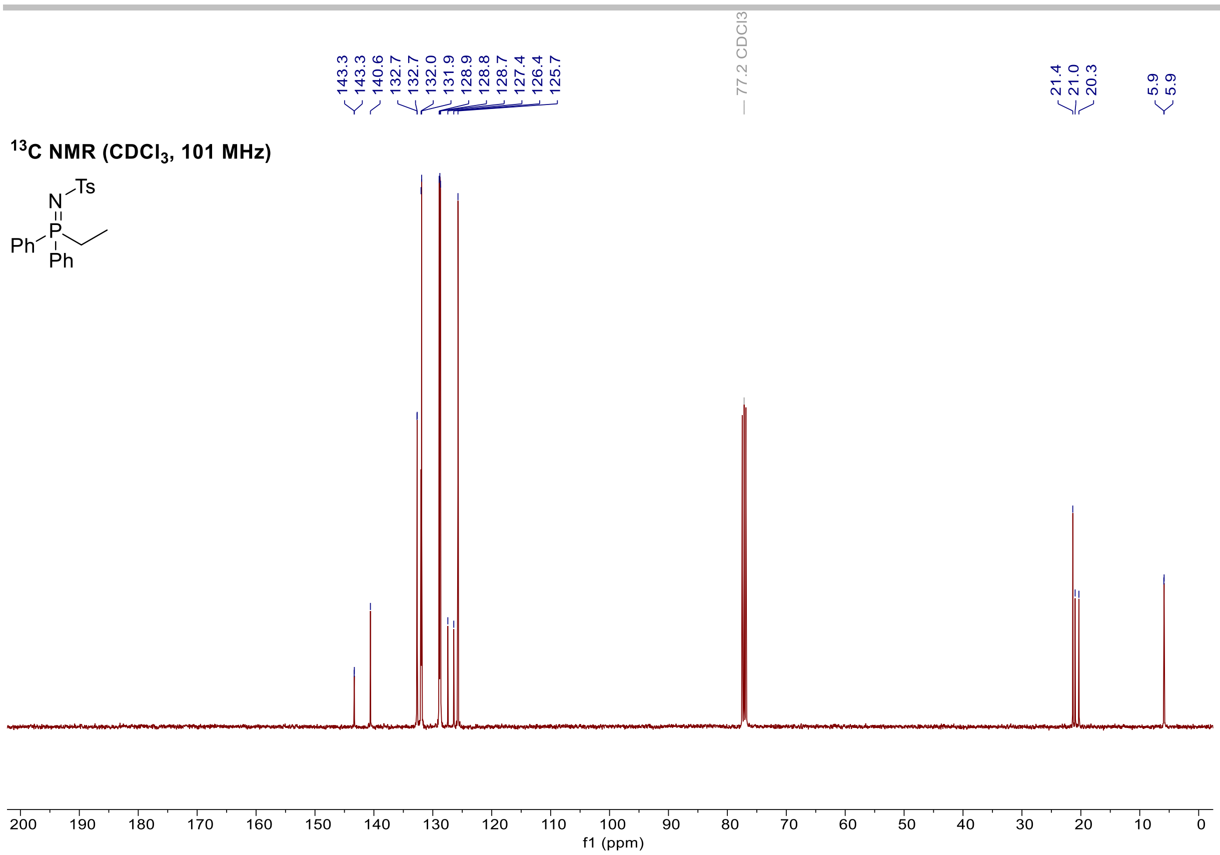
143.3
143.3
140.6
132.7
132.7
132.0
131.9
128.9
128.8
128.7
127.4
126.4
125.7
77.2 CDCl3
21.4
21.0
20.3
5.9
5.9
^{13}C NMR (CDCl$_3$, 101 MHz)
Ts
N
P
Ph
Ph
200 190 180 170 160 150 140 130 120 110 100 90 80 70 60 50 40 30 20 10 0
f1 (ppm)

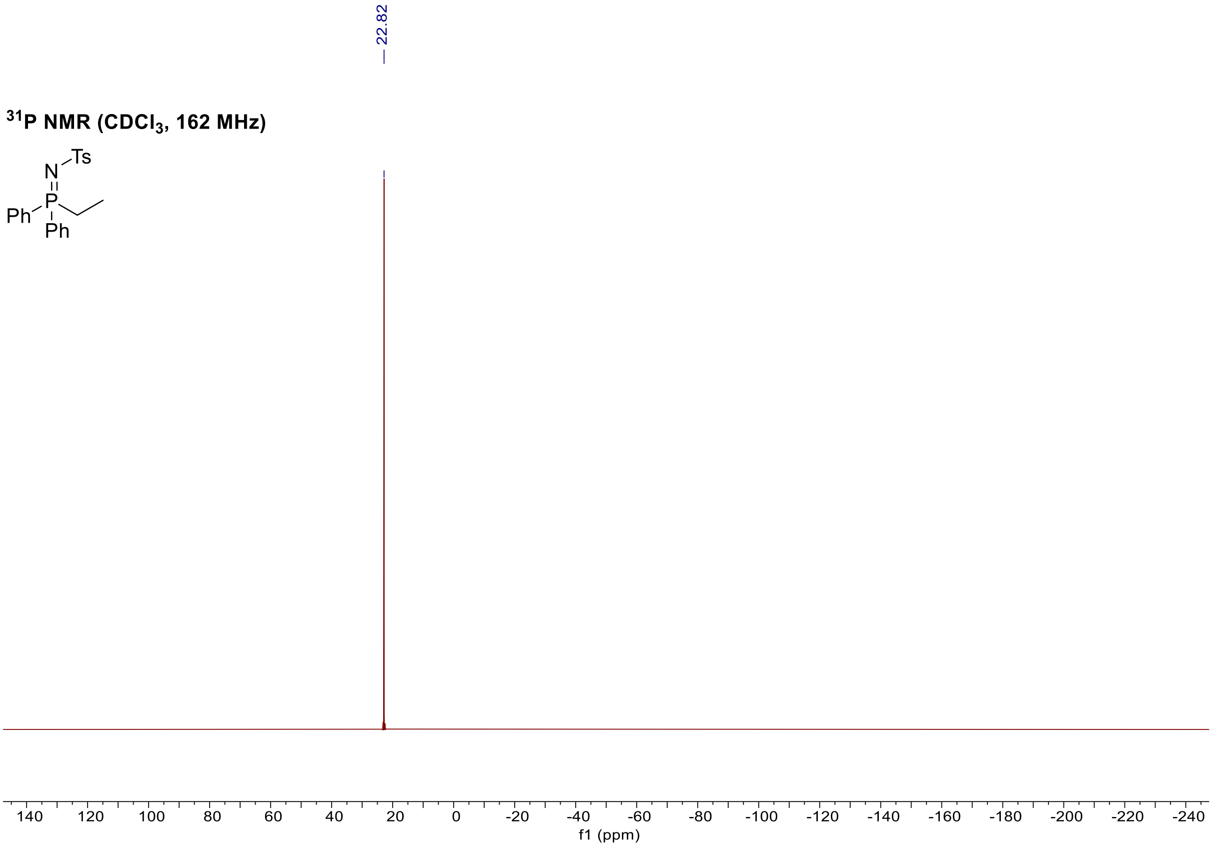
22.82
^{31}P NMR (CDCl$_3$, 162 MHz)
Ts
N
P
Ph
Ph
140 120 100 80 60 40 20 0 -20 -40 -60 -80 -100 -120 -140 -160 -180 -200 -220 -240
f1 (ppm)

N-(Diphenyl(4-vinylphenyl)-λ⁵-phosphanylidene)-4-methylbenzenesulfonamide (13)

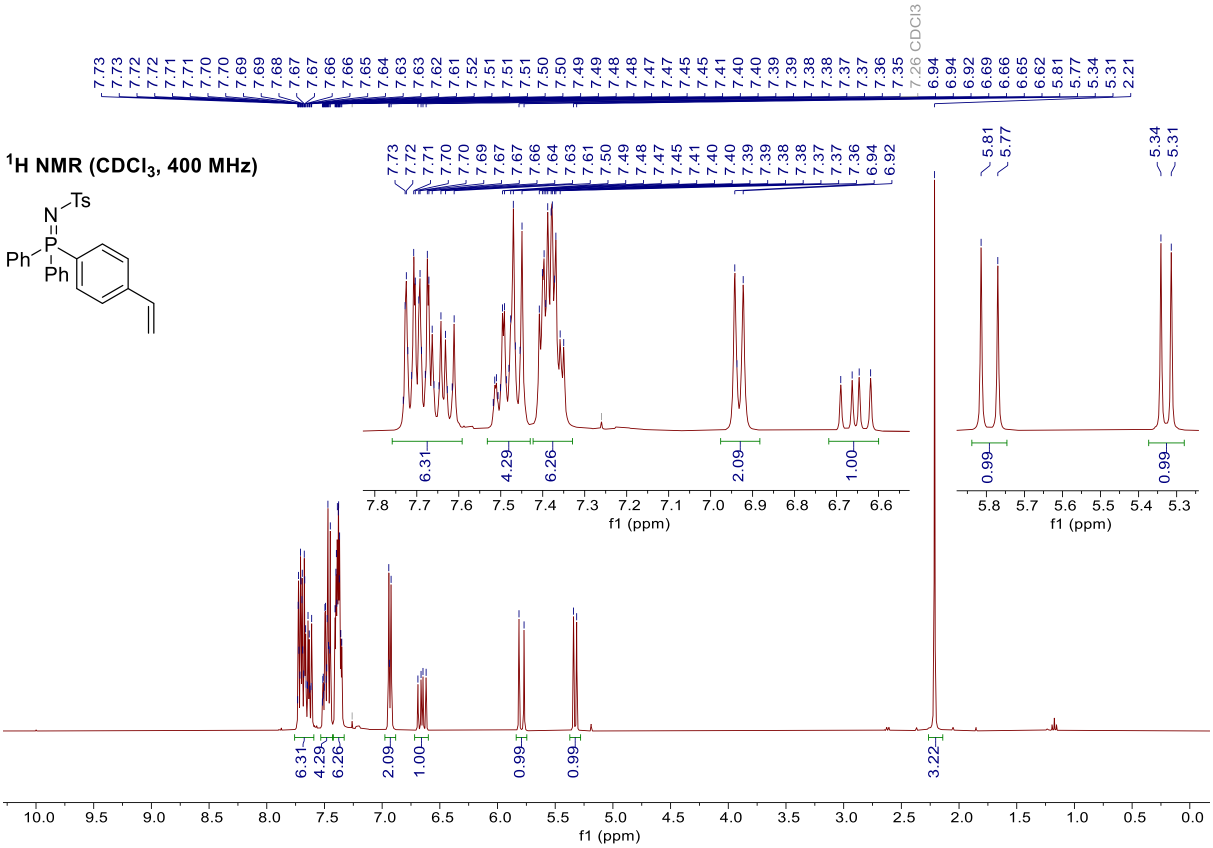

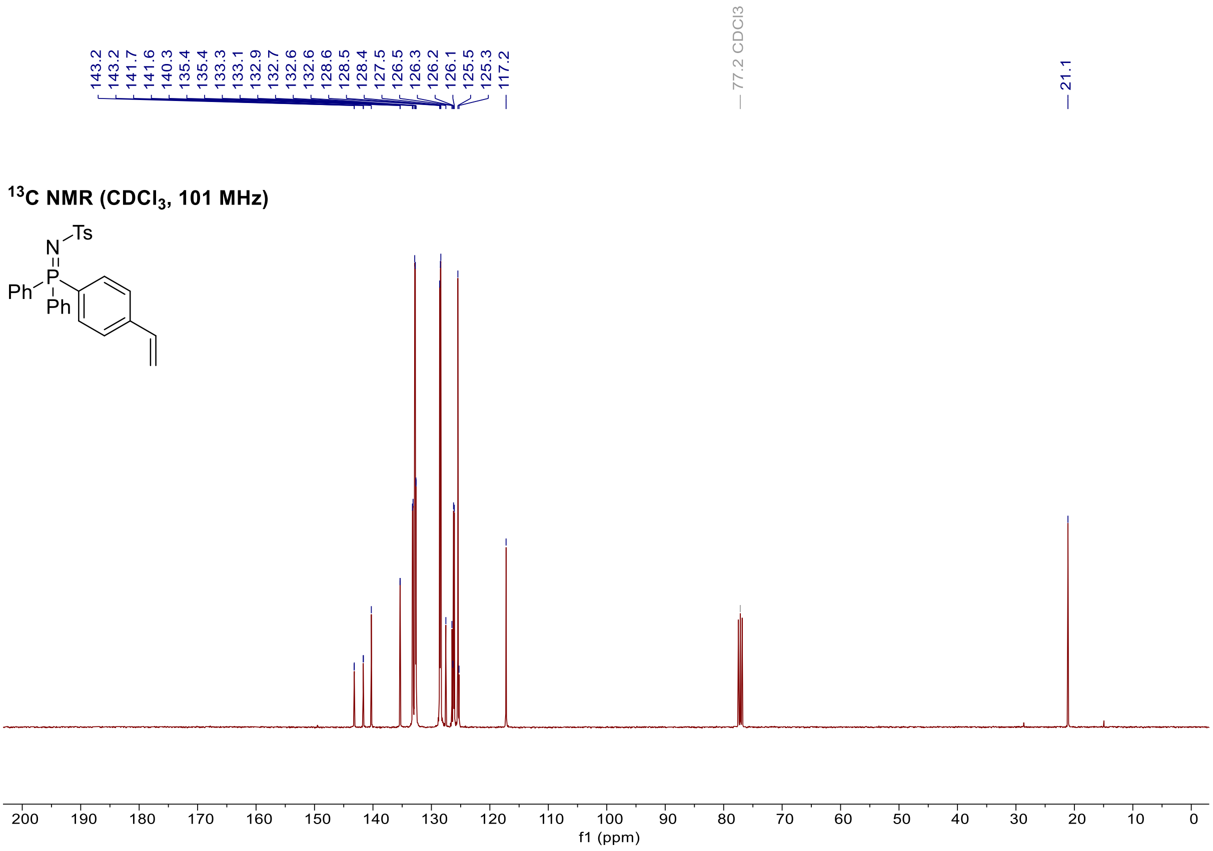

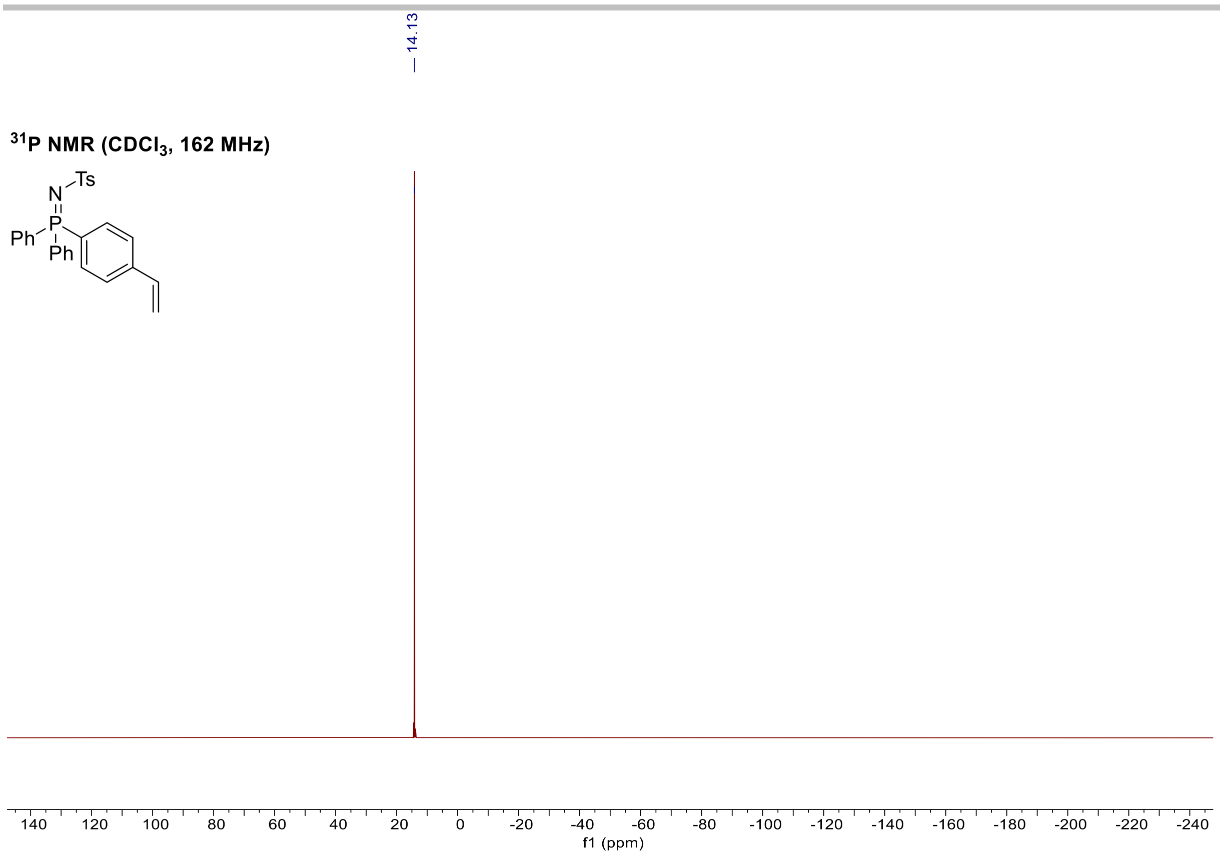

***N*-((4-Ethylphenyl)diphenyl-λ^5-phosphanylidene)-4-methylbenzenesulfonamide (13')**

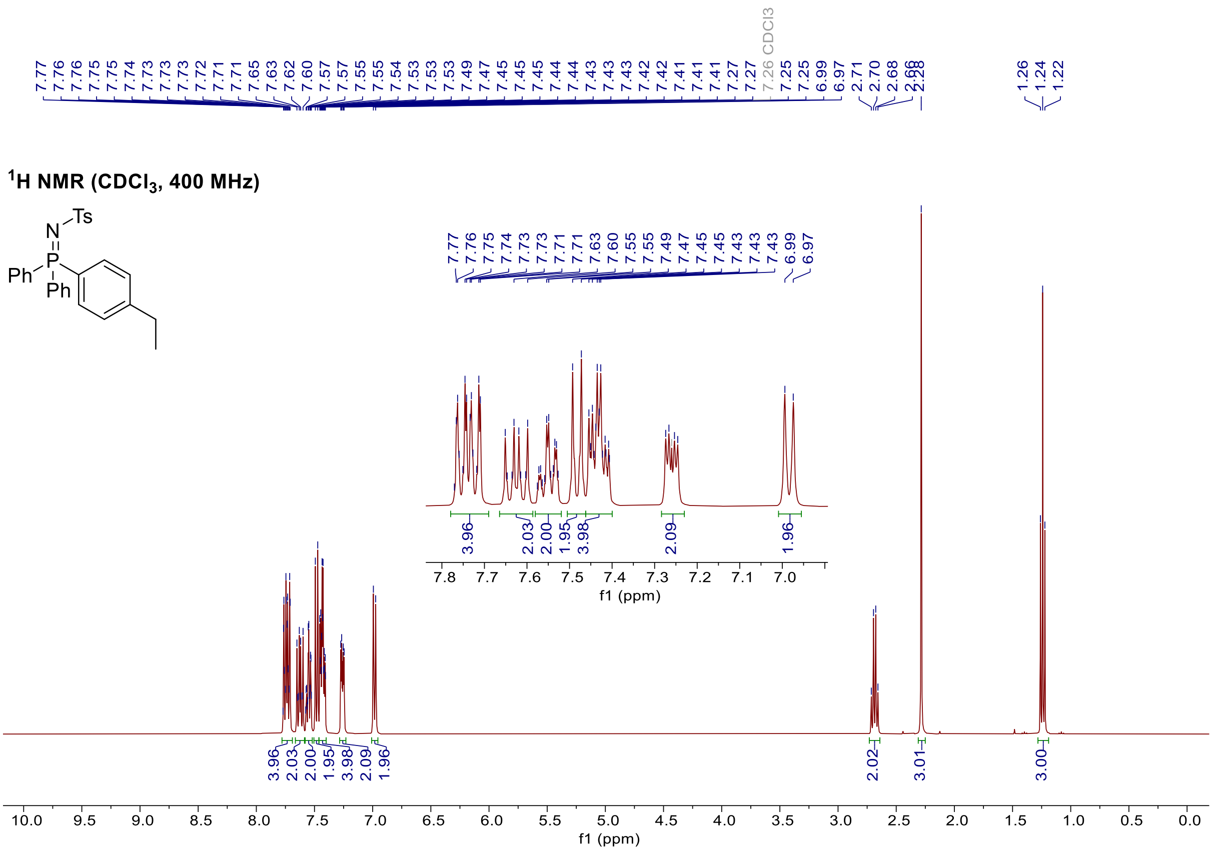

^{13}C NMR ($CDCl_3$, 101 MHz)

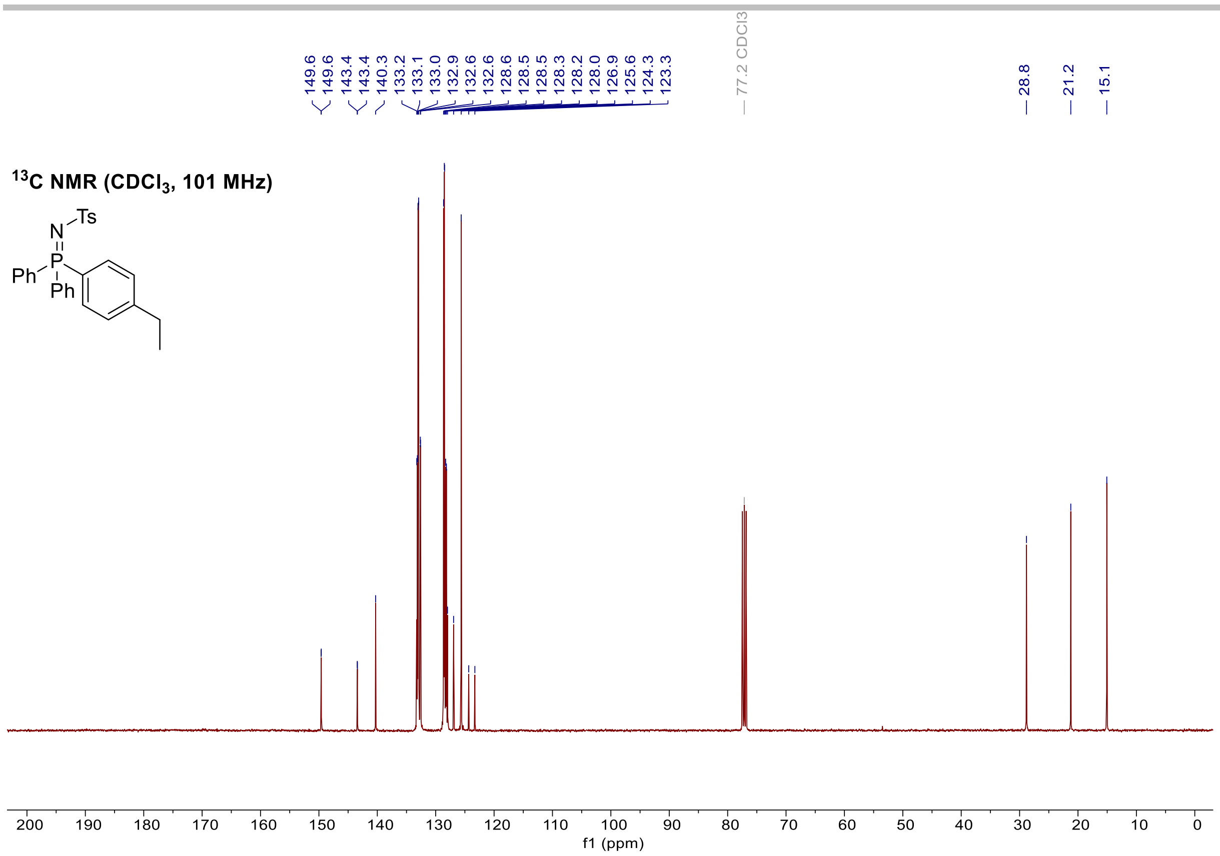

^{31}P NMR ($CDCl_3$, 162 MHz)

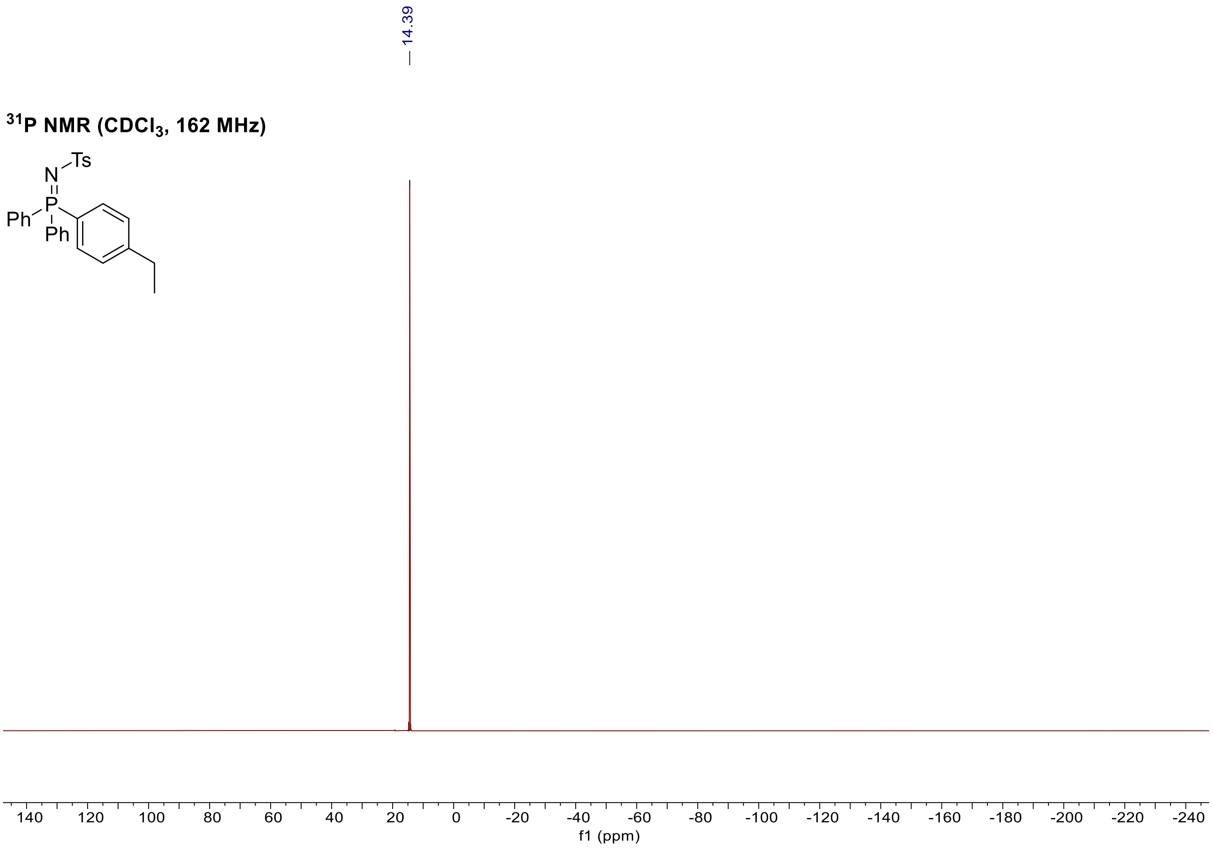

4-Methoxy-*N*-(triphenyl-λ^5-phosphanylidene)benzenesulfonamide (14)

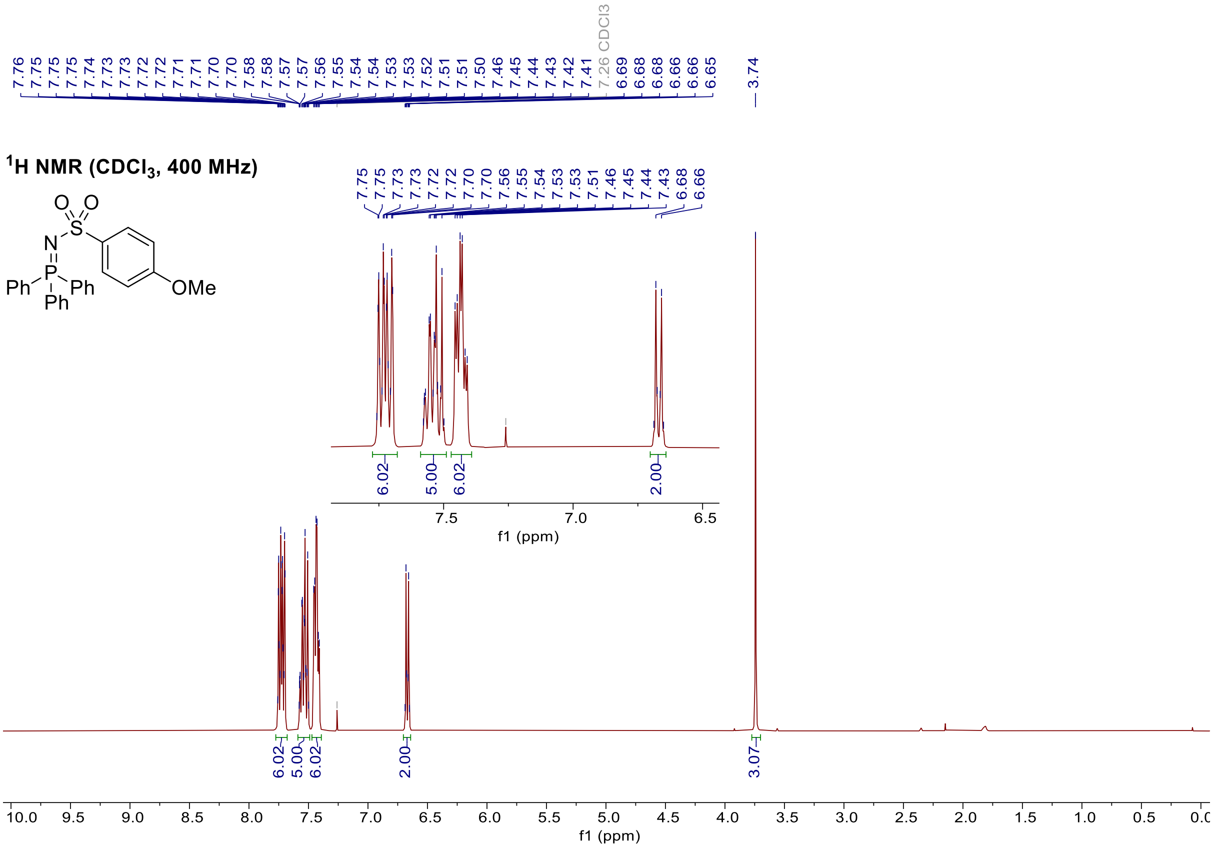

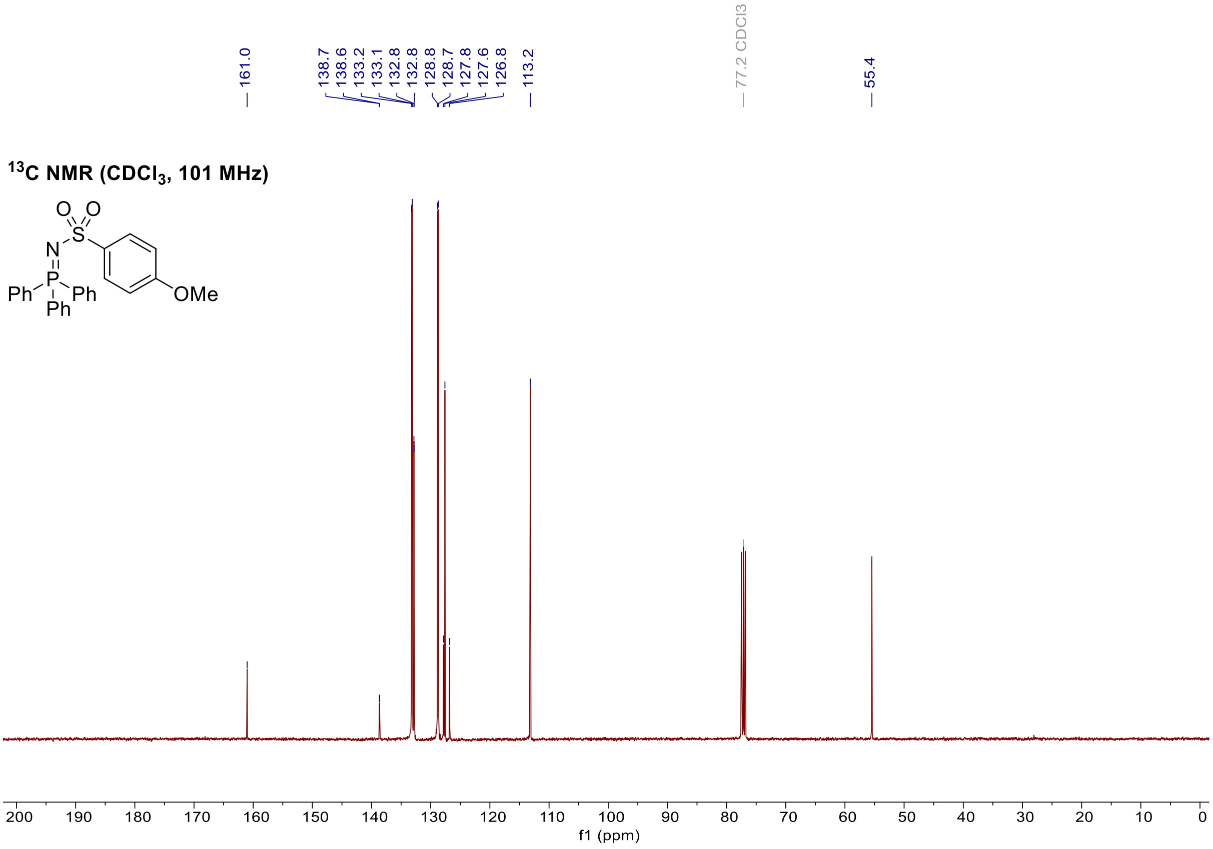

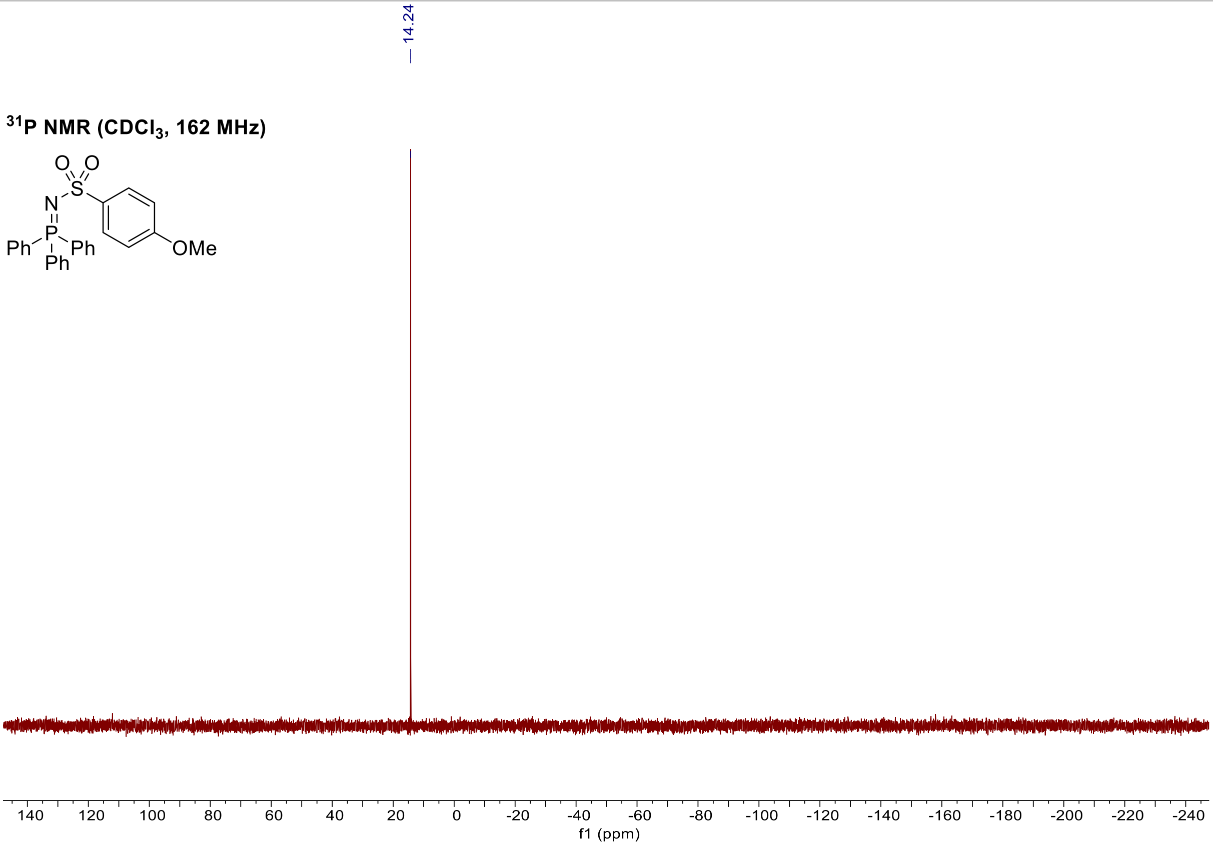

4-Chloro-*N*-(triphenyl-λ^5-phosphanylidene)benzenesulfonamide (15)

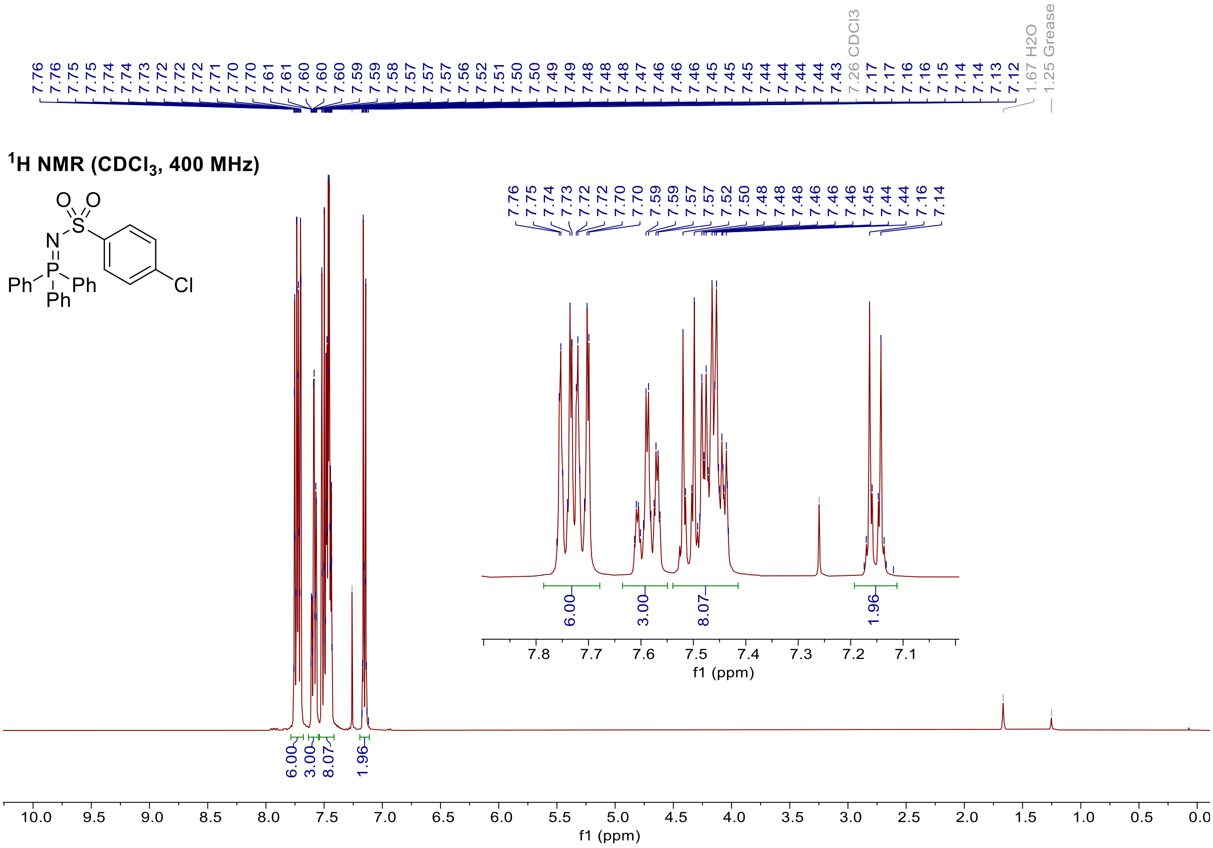

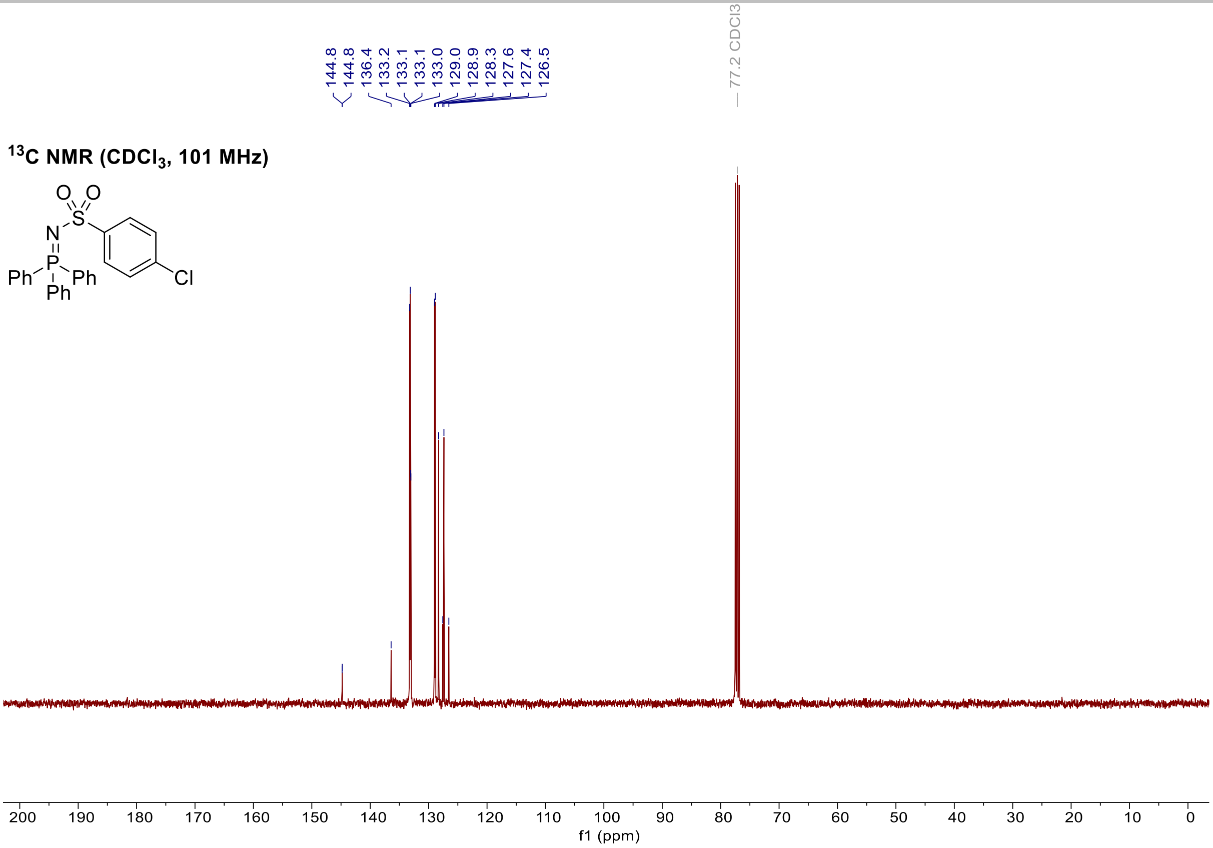
144.8
144.8
136.4
133.2
133.1
133.1
133.0
129.0
128.9
128.3
127.6
127.4
126.5
77.2 CDCl3
13C NMR (CDCl3, 101 MHz)
Ph
Ph
Ph
Cl
200 190 180 170 160 150 140 130 120 110 100 90 80 70 60 50 40 30 20 10 0
f1 (ppm)

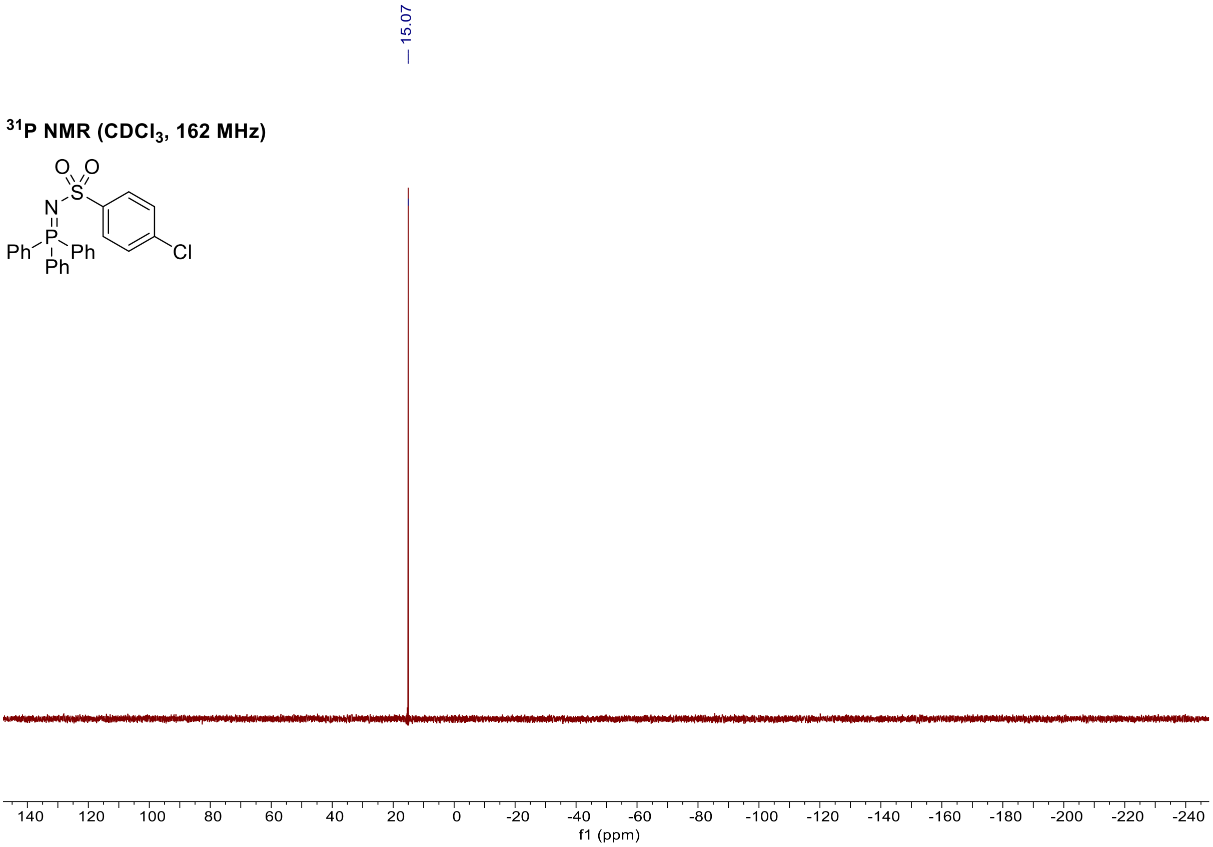
15.07
31P NMR (CDCl3, 162 MHz)
Ph
Ph
Ph
Cl
140 120 100 80 60 40 20 0 -20 -40 -60 -80 -100 -120 -140 -160 -180 -200 -220 -240
f1 (ppm)

4-Amino-*N*-(triphenyl-λ⁵-phosphanylidene)benzenesulfonamide (16)

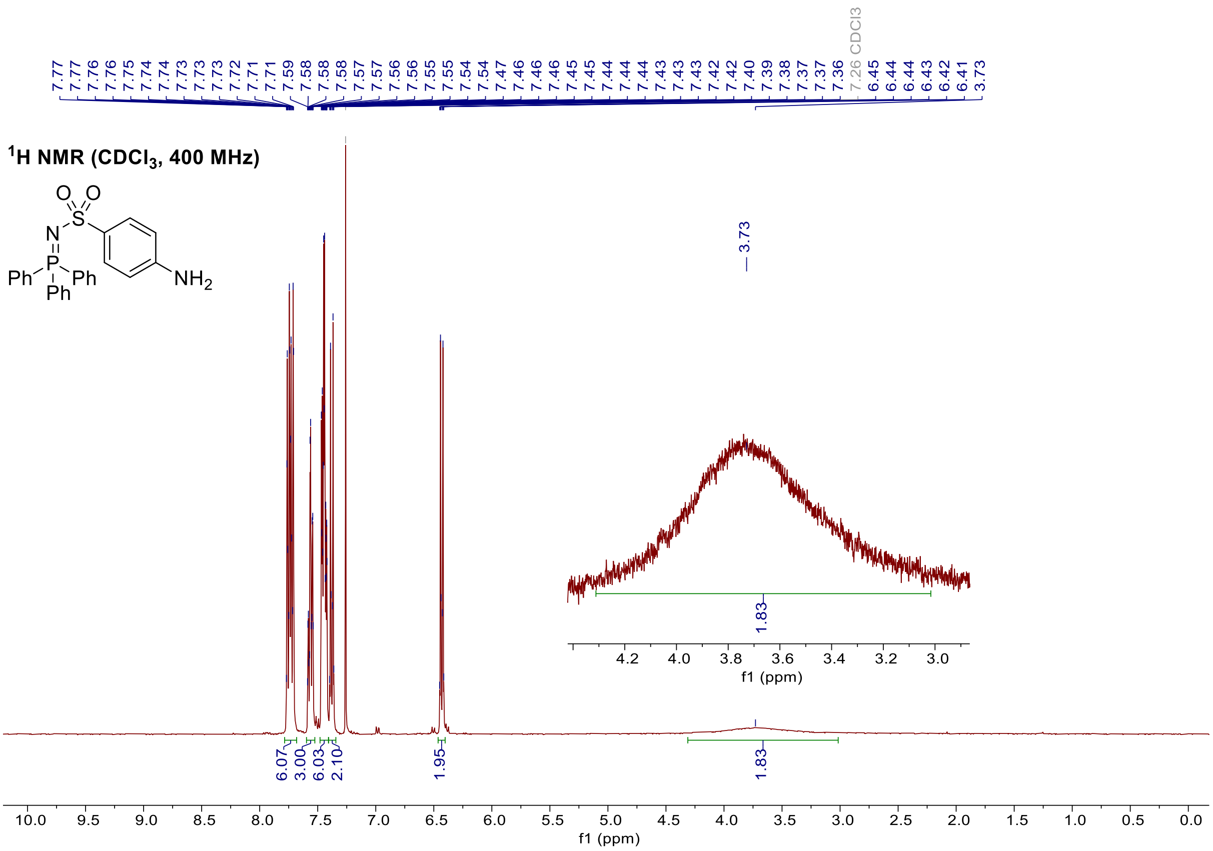

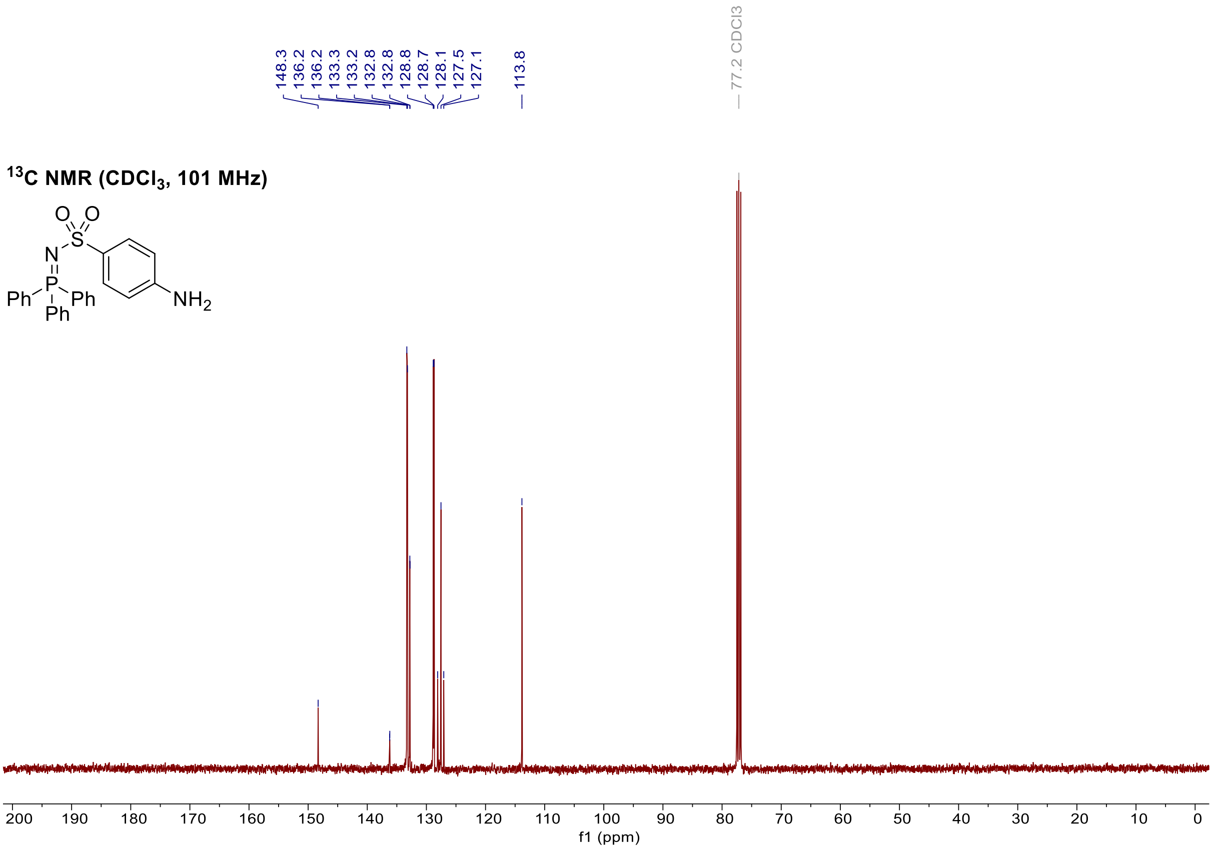

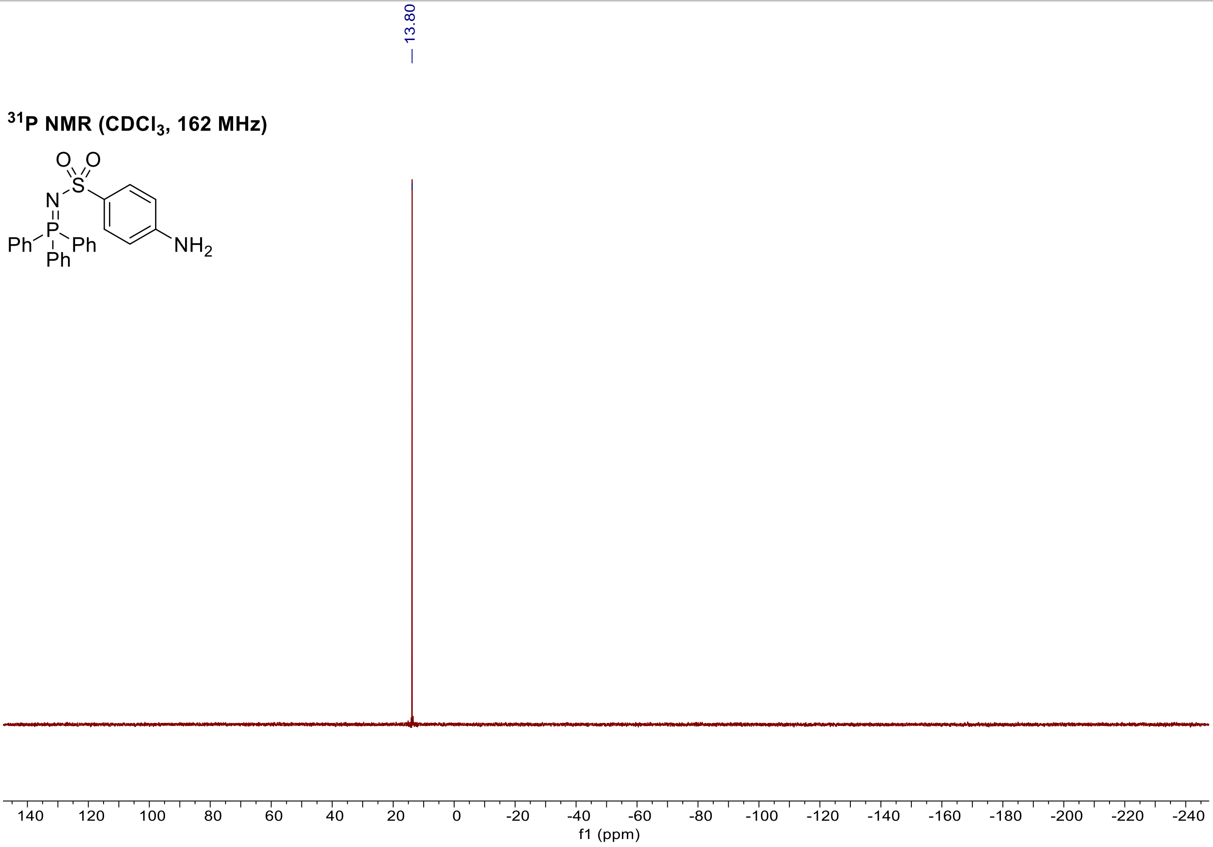

4-Nitro-*N*-(triphenyl-λ^5-phosphanylidene)benzenesulfonamide (17)

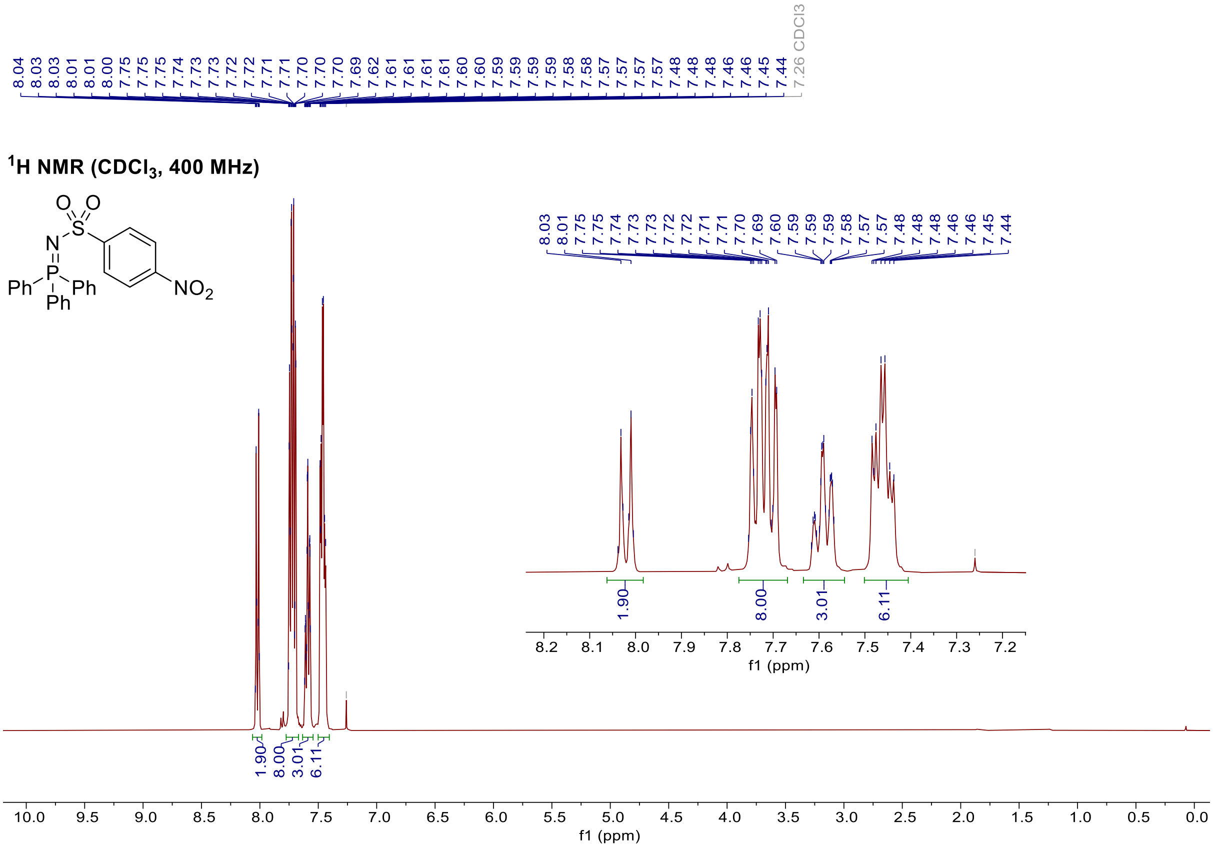

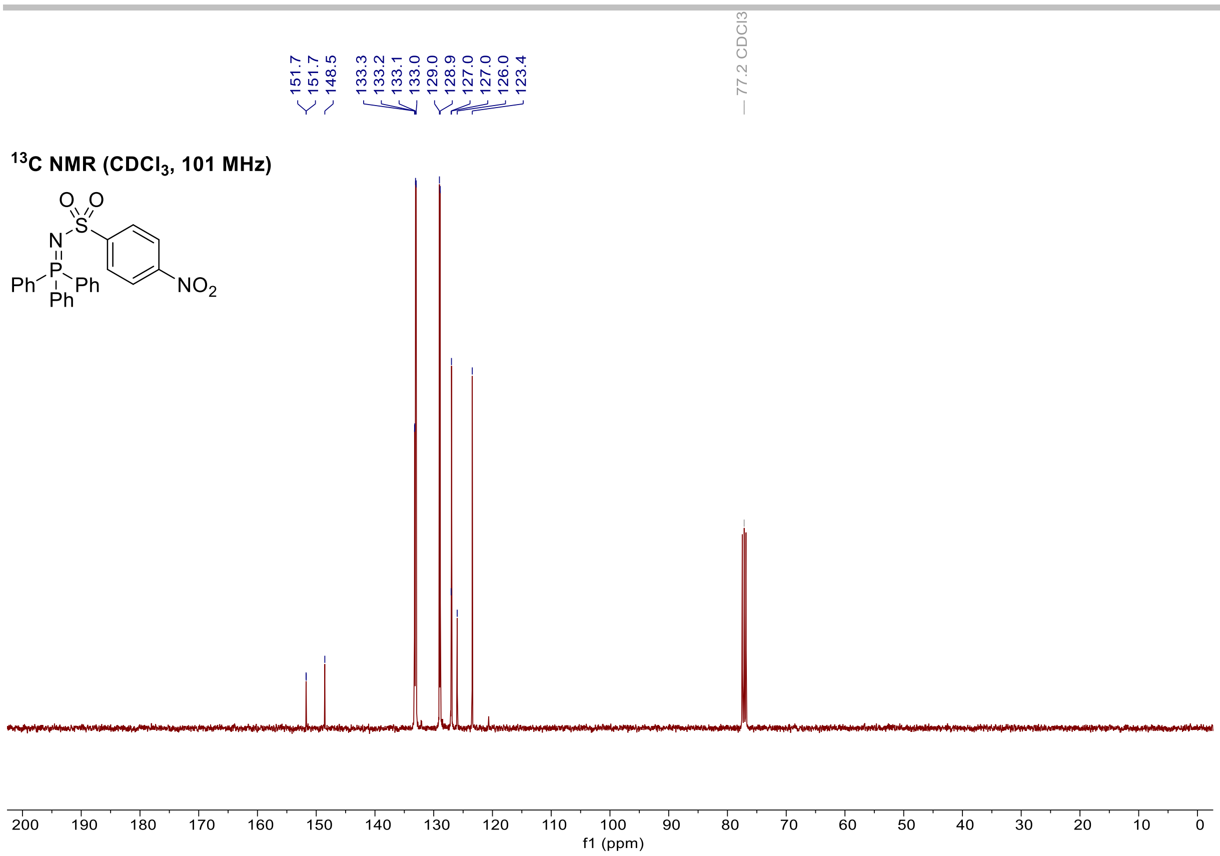

151.7
151.7
148.5
133.3
133.2
133.1
133.0
129.0
128.9
127.0
127.0
126.0
123.4
77.2 CDCl3
13C NMR (CDCl3, 101 MHz)
O O
S
N
P
Ph Ph
Ph
NO2
200 190 180 170 160 150 140 130 120 110 100 90 80 70 60 50 40 30 20 10 0
f1 (ppm)

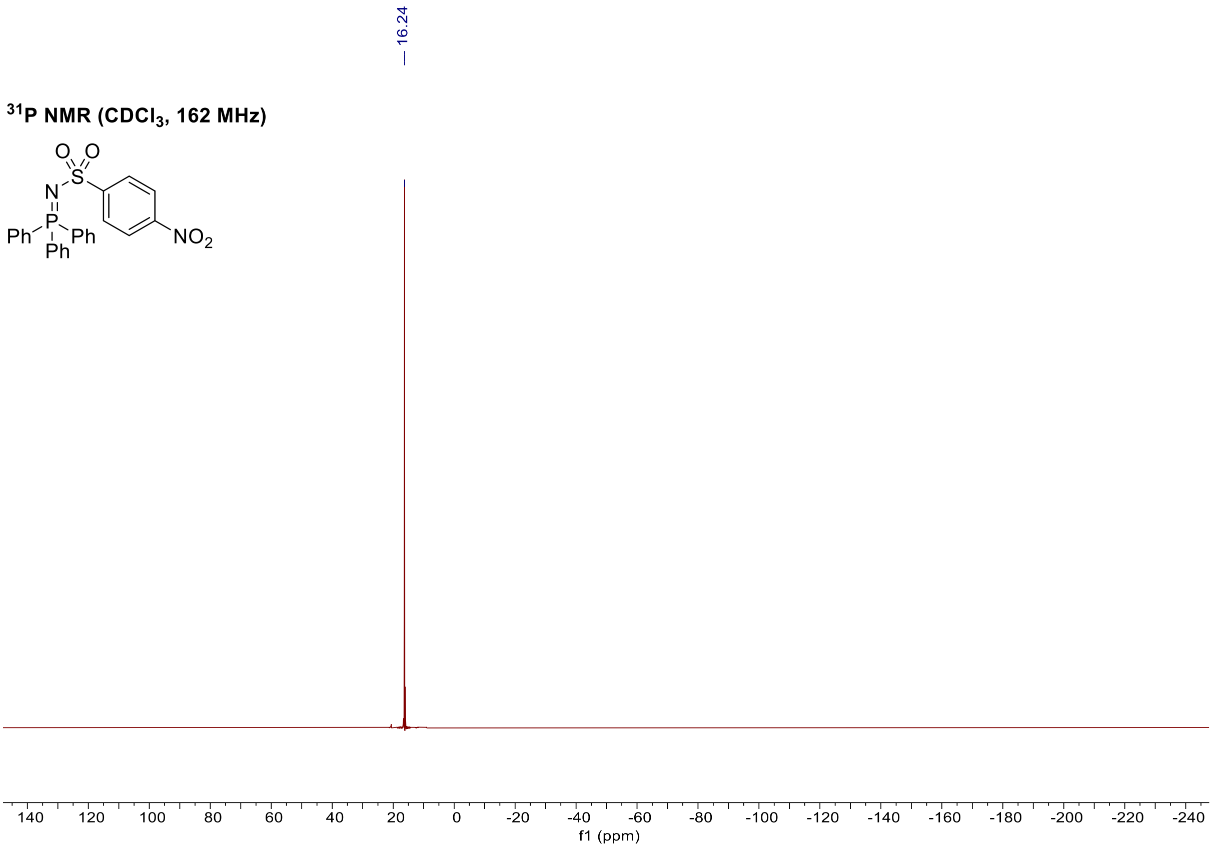

16.24
31P NMR (CDCl3, 162 MHz)
O O
S
N
P
Ph Ph
Ph
NO2
140 120 100 80 60 40 20 0 -20 -40 -60 -80 -100 -120 -140 -160 -180 -200 -220 -240
f1 (ppm)

N-(Triphenyl-λ5-phosphanylidene)benzenesulfonamide (18)

^{1}H NMR (CDCl$_3$, 400 MHz)

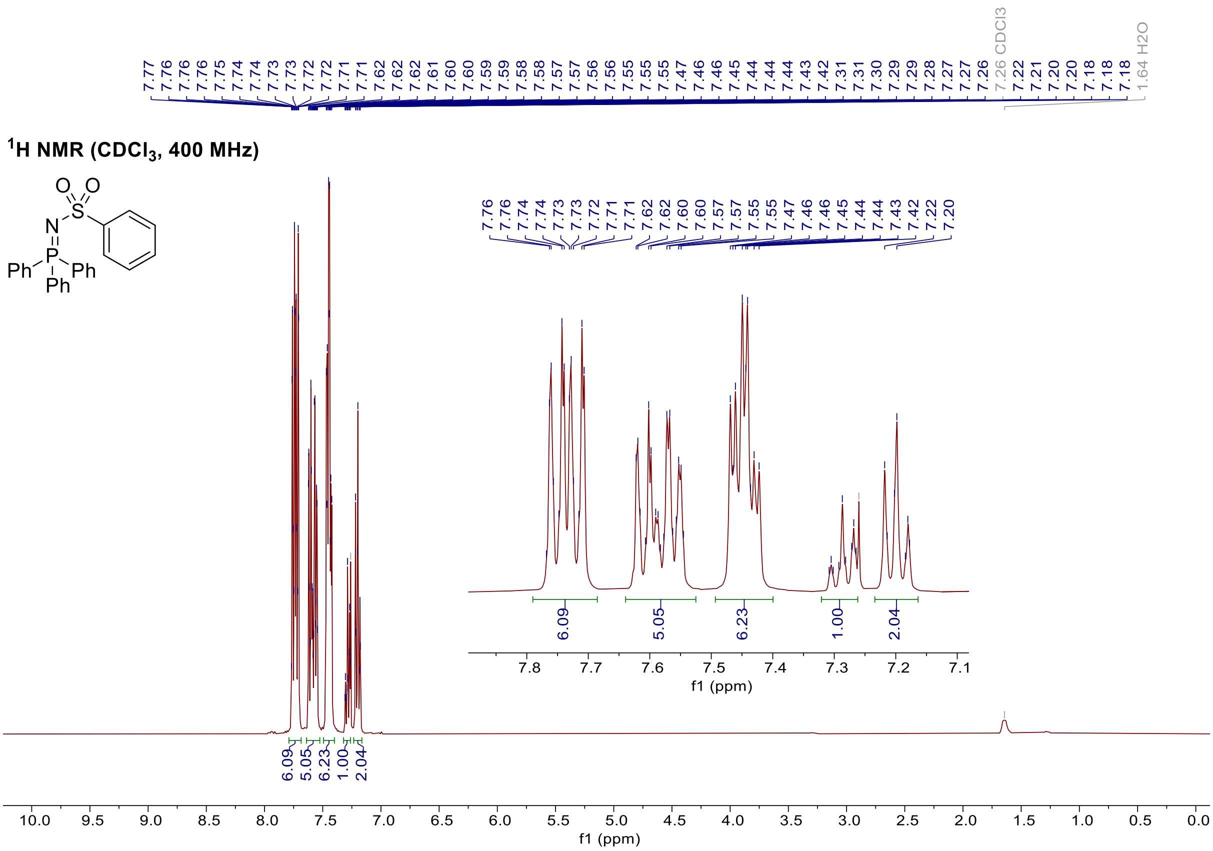

^{13}C NMR (CDCl$_3$, 101 MHz)

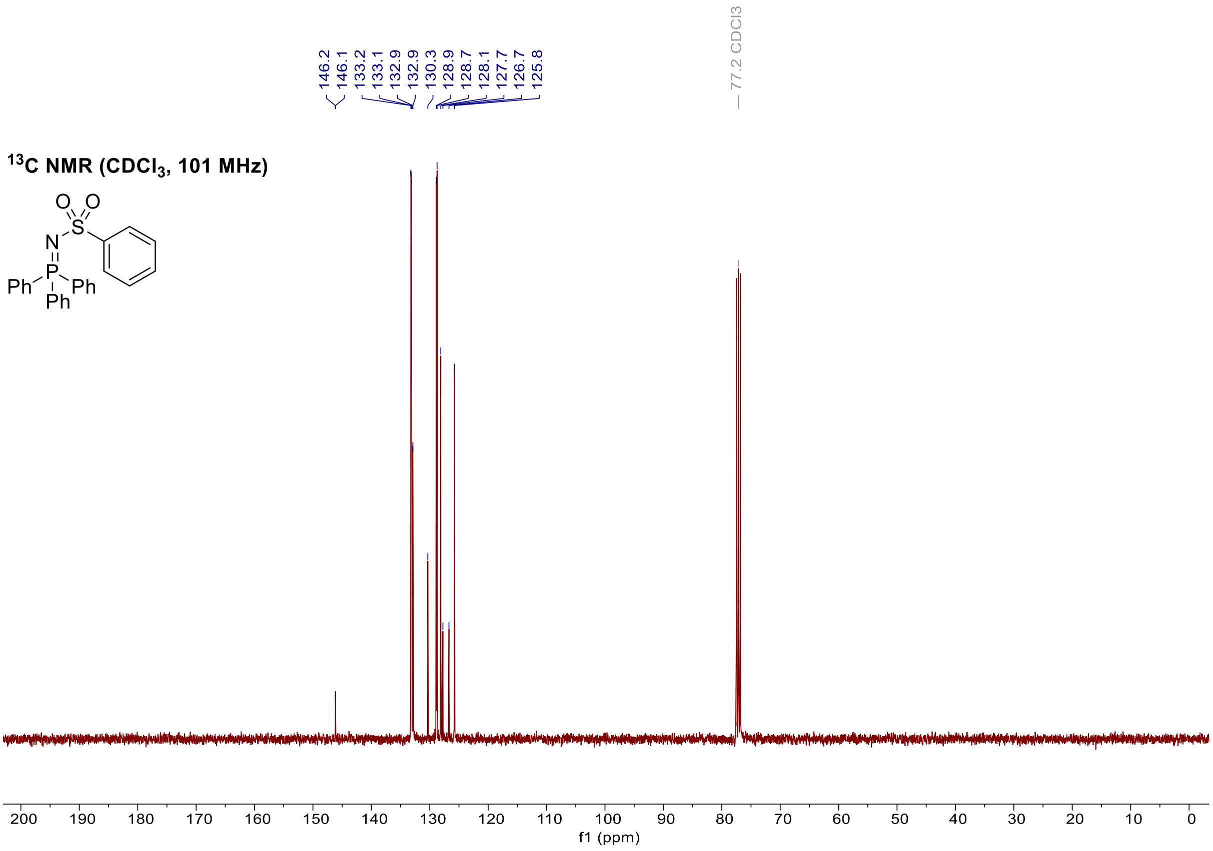

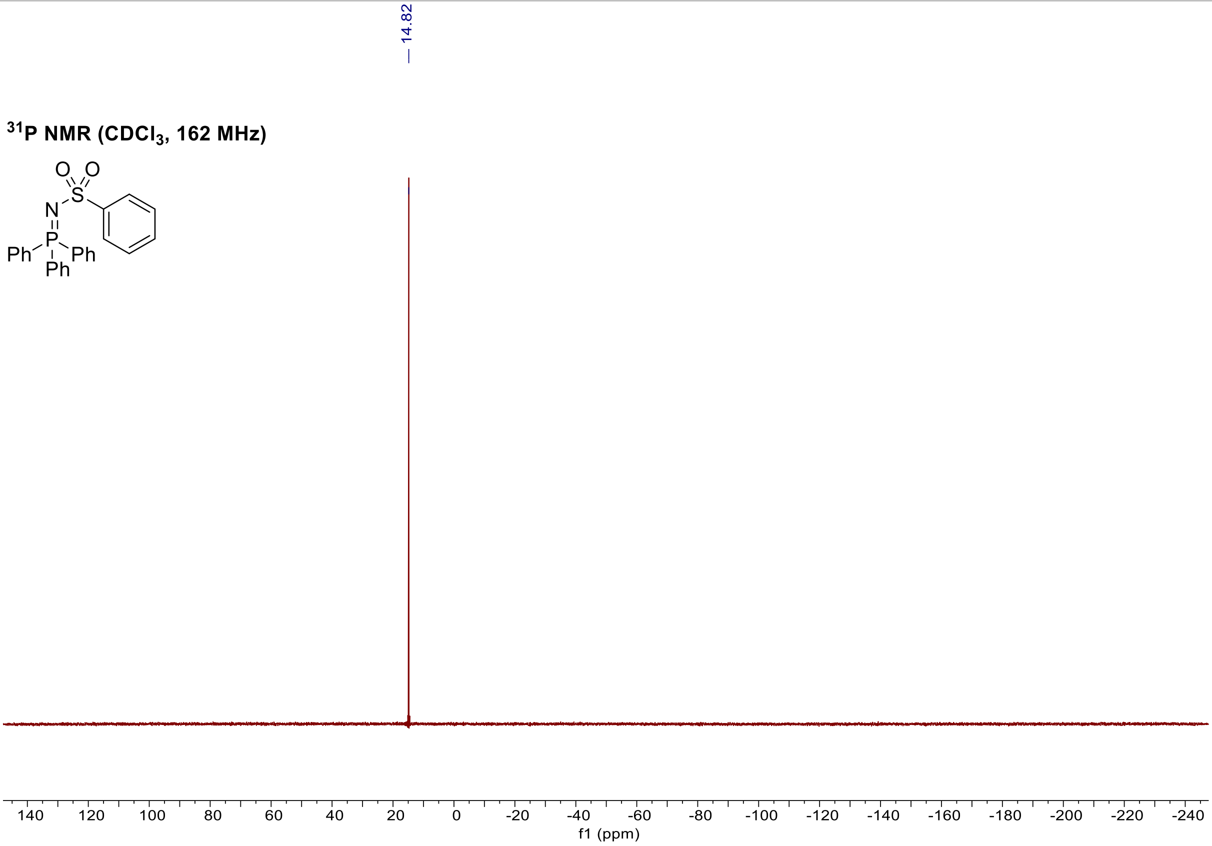

2,4,6-Triisopropyl-*N*-(triphenyl-λ^5-phosphanylidene)benzenesulfonamide (19)

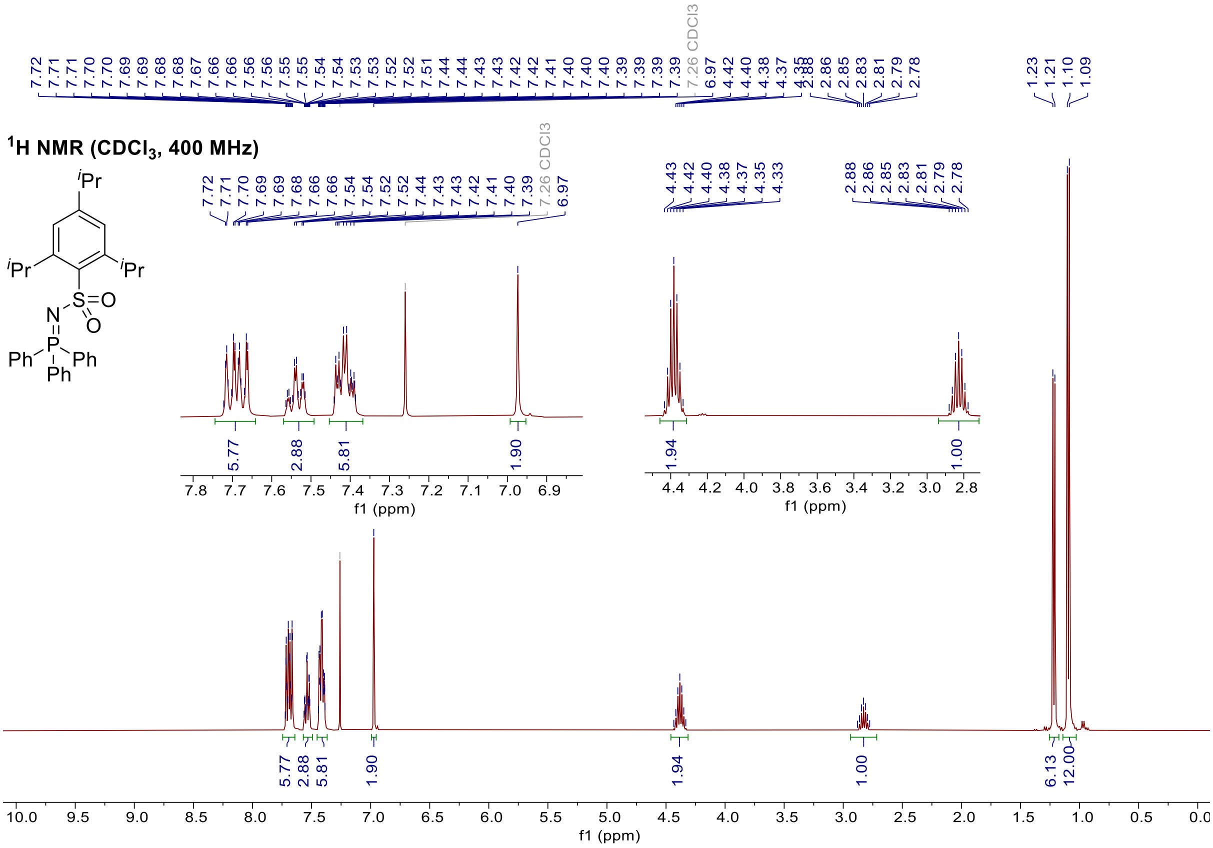

^{13}C NMR ($CDCl_3$, 101 MHz)

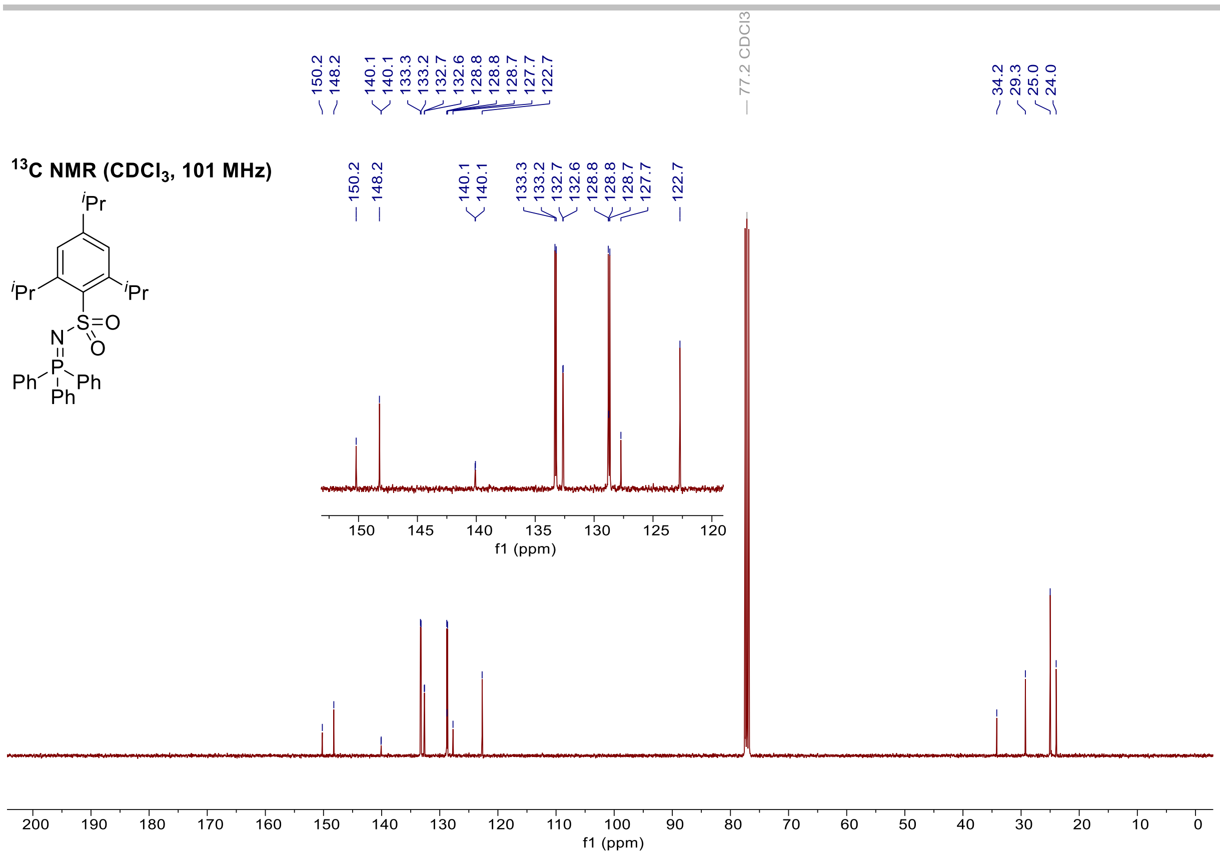

^{31}P NMR ($CDCl_3$, 162 MHz)

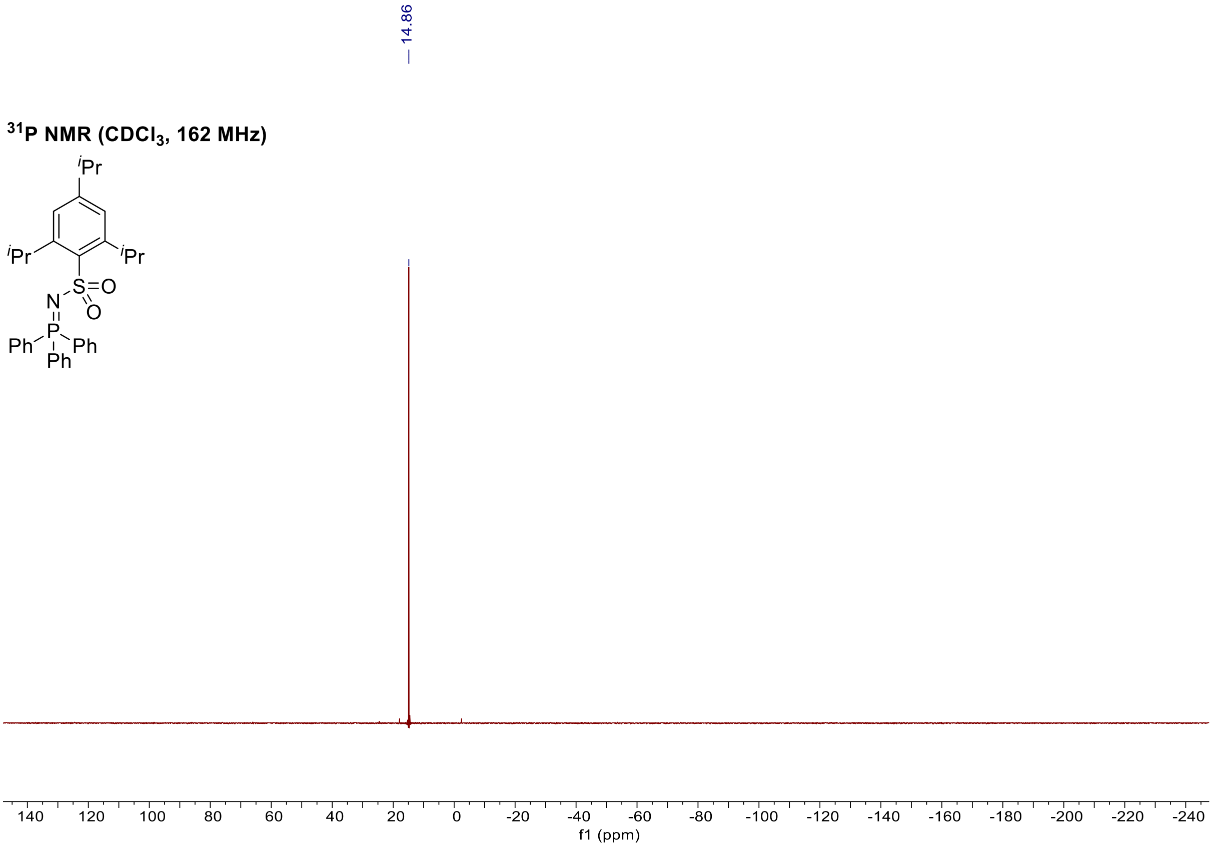

N-(Triphenyl-λ5-phosphanylidene)methanesulfonamide (20)

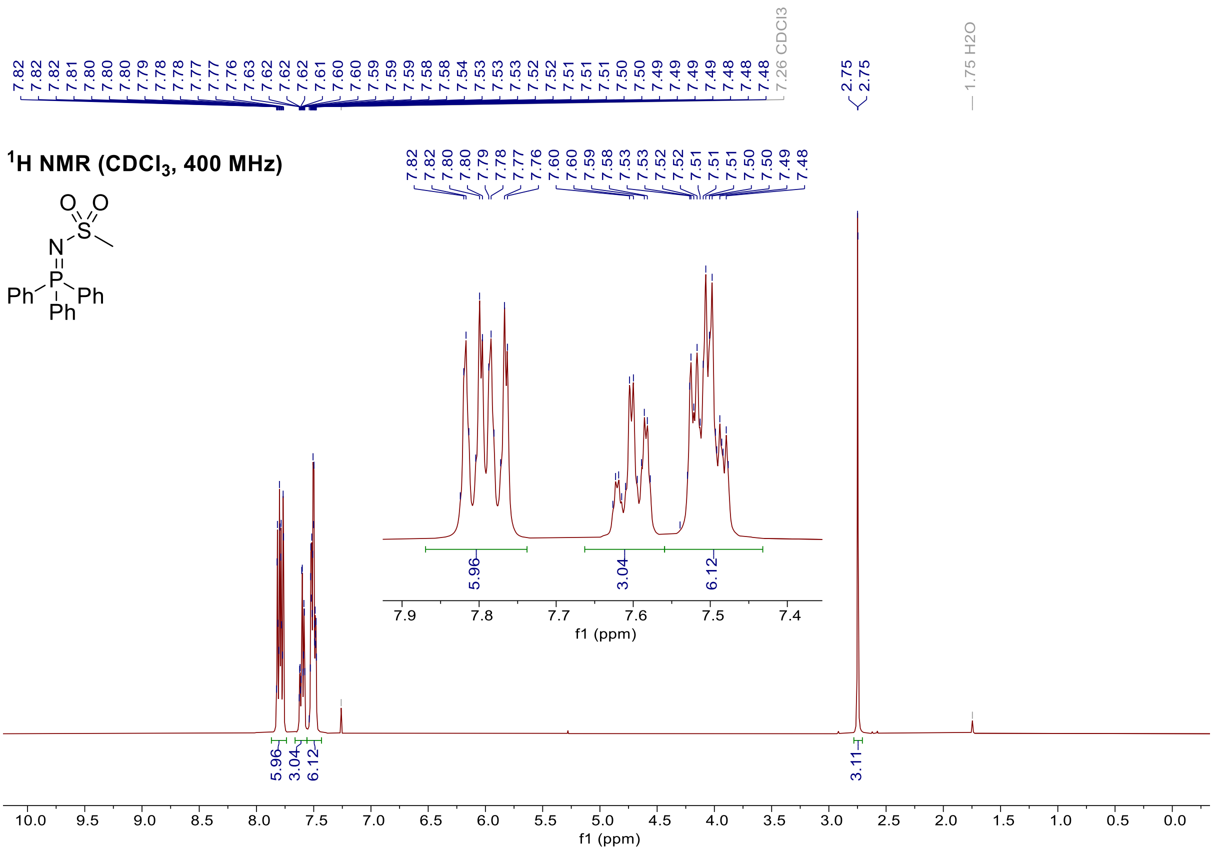

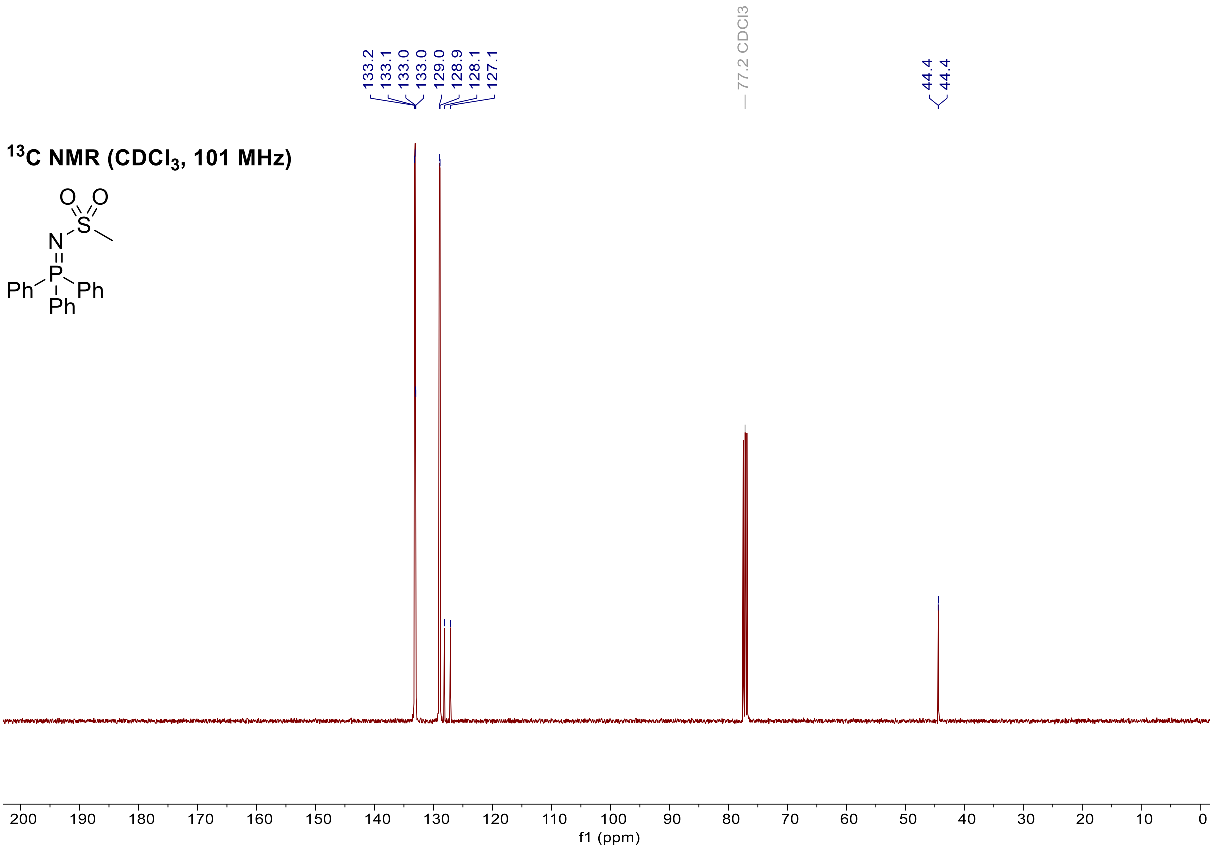

^{31}P NMR (CDCl$_3$, 162 MHz)

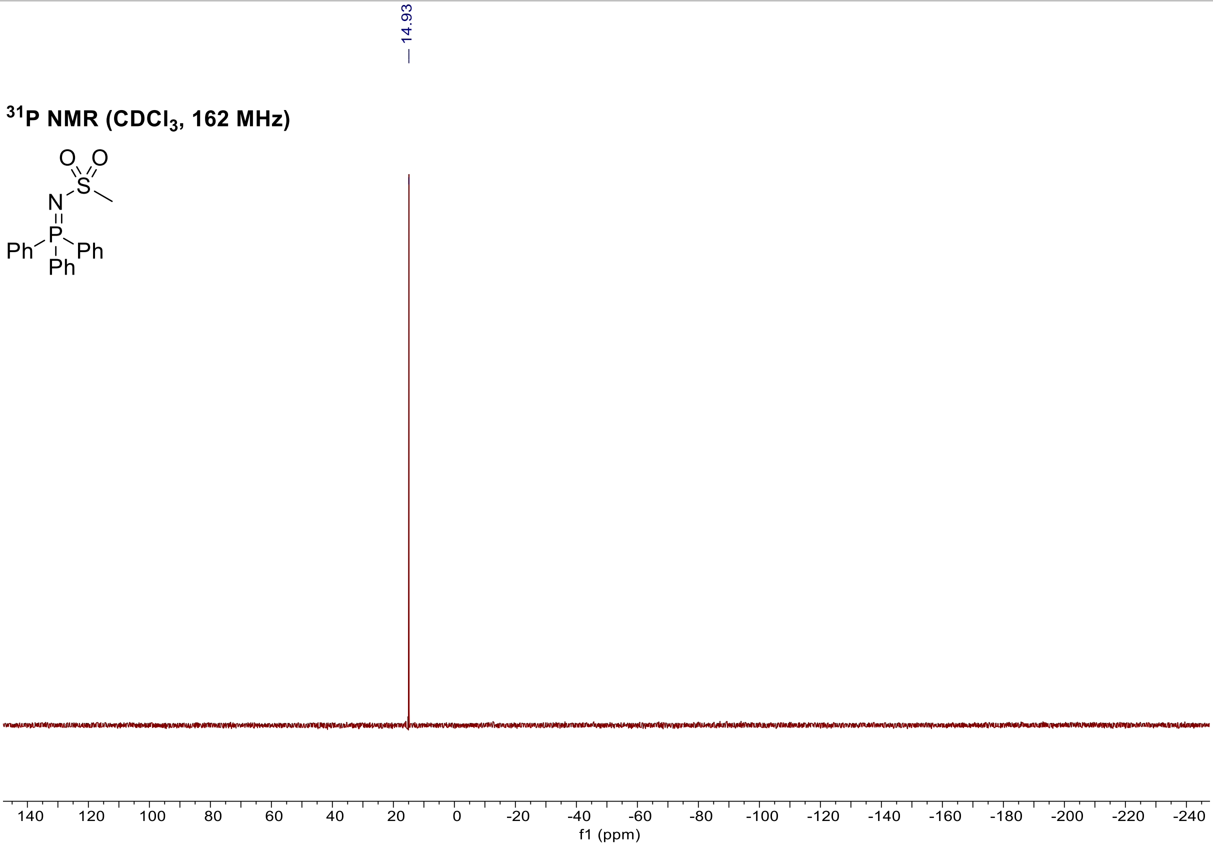

1,1,1-Trifluoro-*N*-(triphenyl-*λ*^{5}-phosphanylidene)methanesulfonamide (21)

^{1}H NMR (CDCl$_3$, 400 MHz)

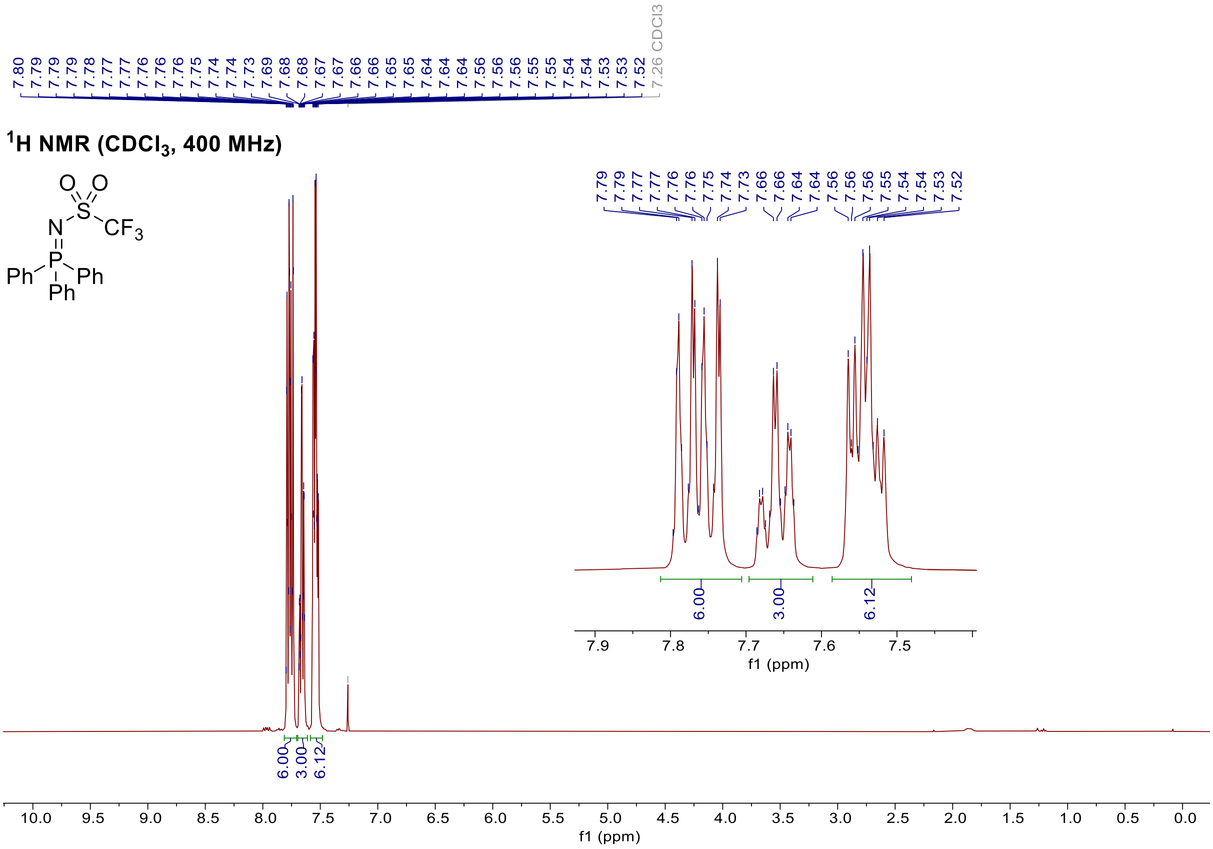

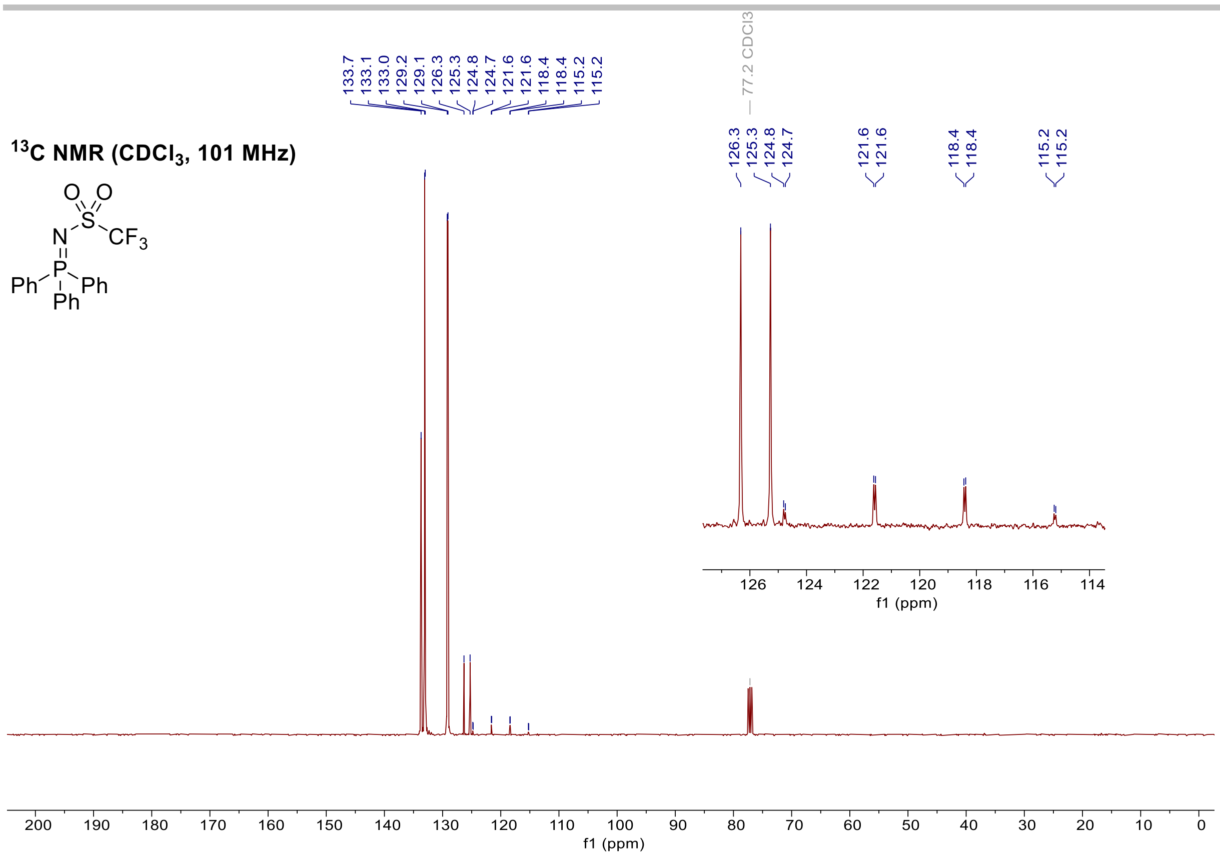
133.7
133.1
133.0
129.2
129.1
126.3
125.3
124.8
124.7
121.6
121.6
118.4
118.4
115.2
115.2
77.2 CDCl3
13C NMR (CDCl3, 101 MHz)
O O
S
N
CF3
P
Ph
Ph
Ph
126.3
125.3
124.8
124.7
121.6
121.6
118.4
118.4
115.2
115.2
126 124 122 120 118 116 114
f1 (ppm)
200 190 180 170 160 150 140 130 120 110 100 90 80 70 60 50 40 30 20 10 0
f1 (ppm)

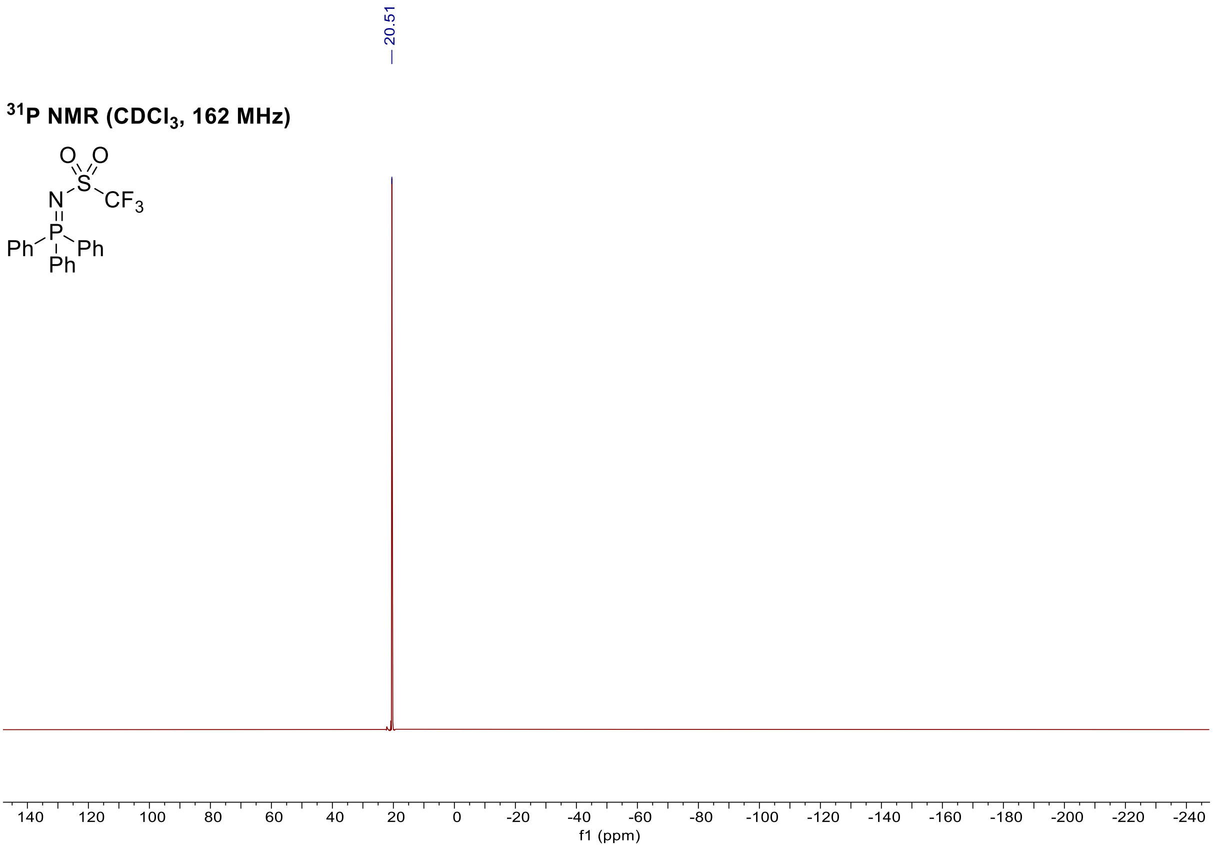
20.51
31P NMR (CDCl3, 162 MHz)
O O
S
N
CF3
P
Ph
Ph
Ph
140 120 100 80 60 40 20 0 -20 -40 -60 -80 -100 -120 -140 -160 -180 -200 -220 -240
f1 (ppm)

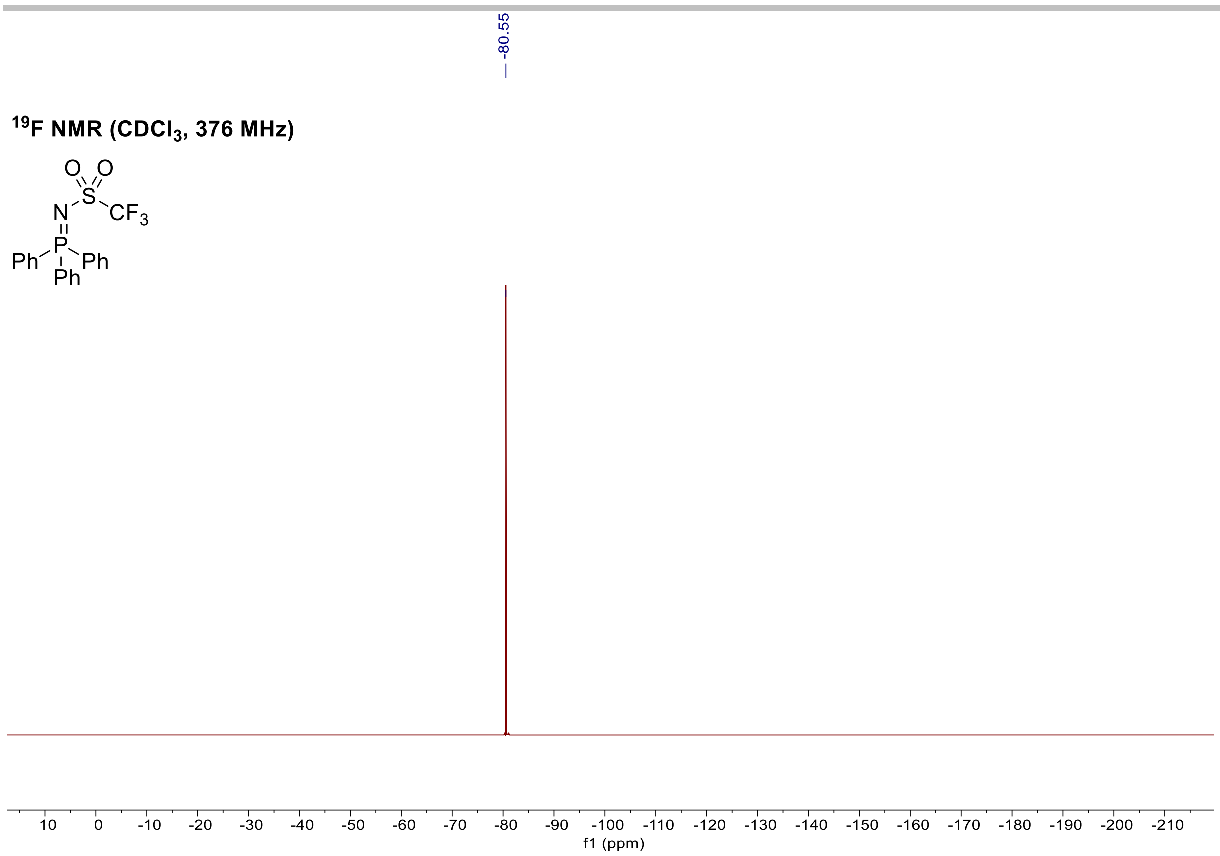

(1*S*)-10-Camphor-(*N*-(triphenyl-λ^5-phosphanylidene))sulfonamide (22)

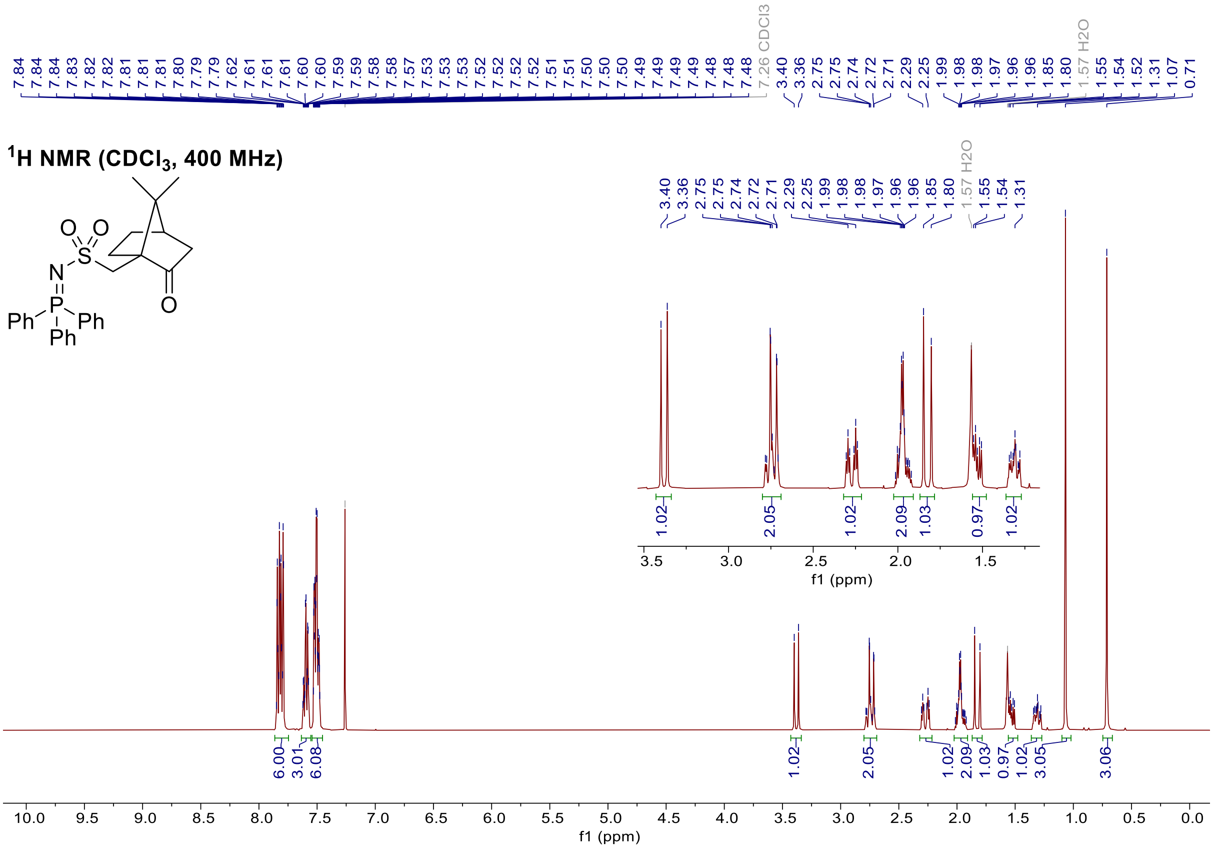

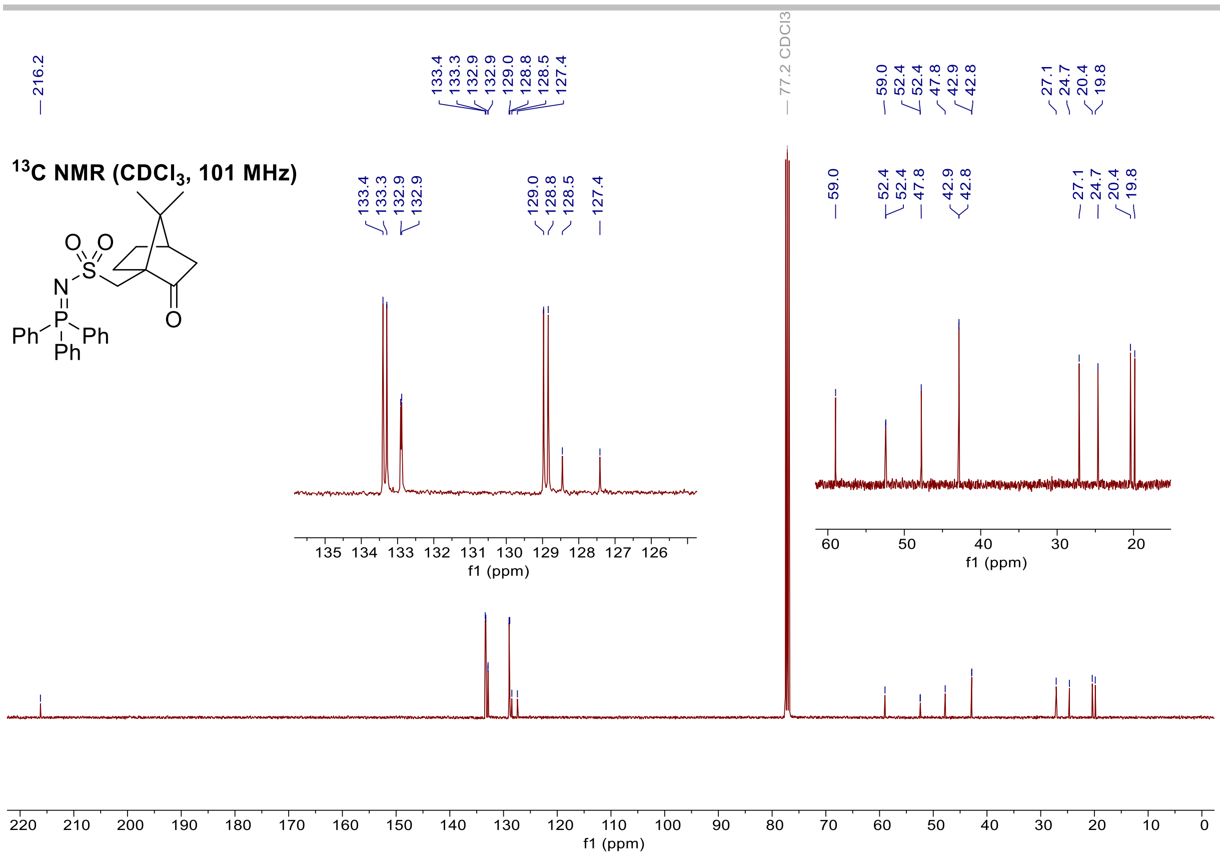
216.2
133.4
133.3
132.9
132.9
129.0
128.8
128.5
127.4
77.2 CDCl3
59.0
52.4
52.4
47.8
42.9
42.8
27.1
24.7
20.4
19.8
13C NMR (CDCl3, 101 MHz)
Ph
Ph
Ph
f1 (ppm)

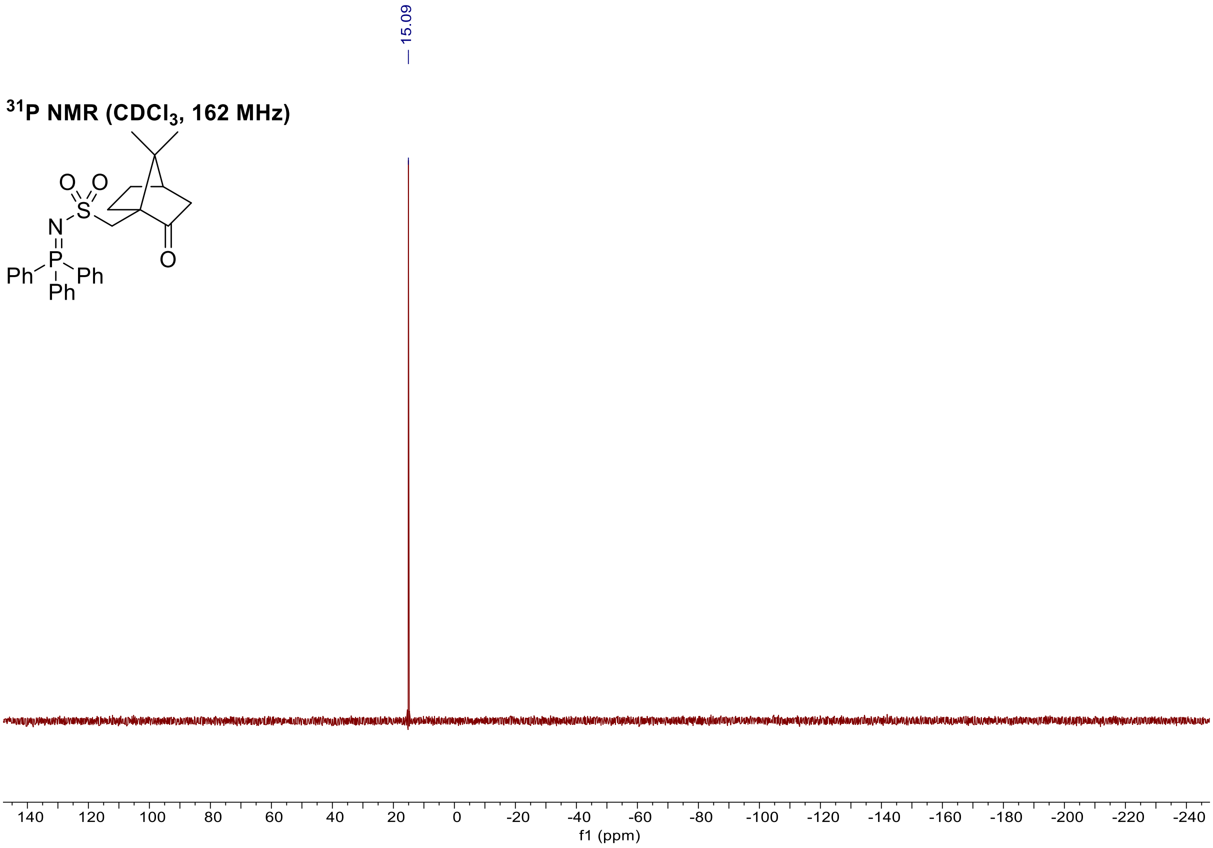
15.09
31P NMR (CDCl3, 162 MHz)
Ph
Ph
Ph
f1 (ppm)

3,3,5,5-Tetraphenyl-4*H*-1,2,6,3λ⁵,5λ⁵-thiadiazadiphosphanine-1,1-dioxide (23)

^{1}H NMR (CD_2Cl_2, 400 MHz)

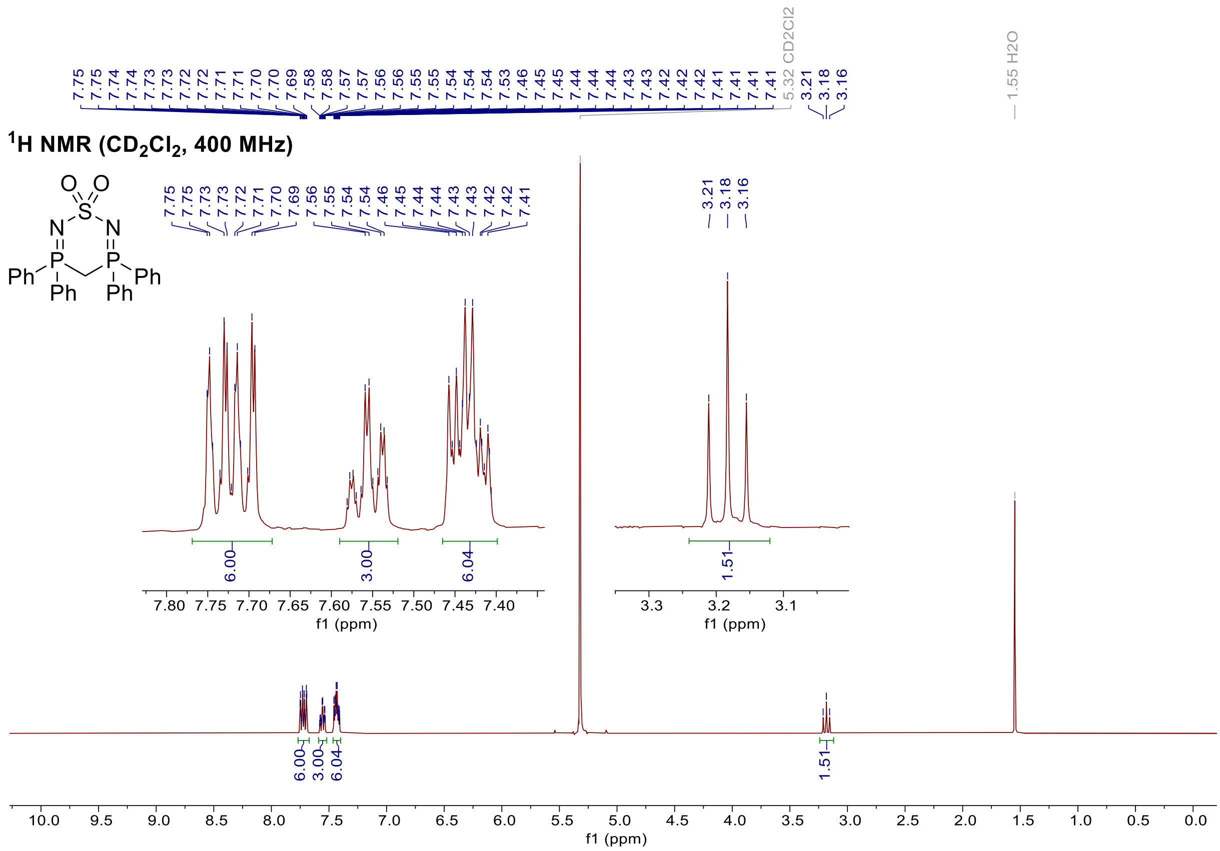

^{13}C NMR (CD_2Cl_2, 151 MHz)

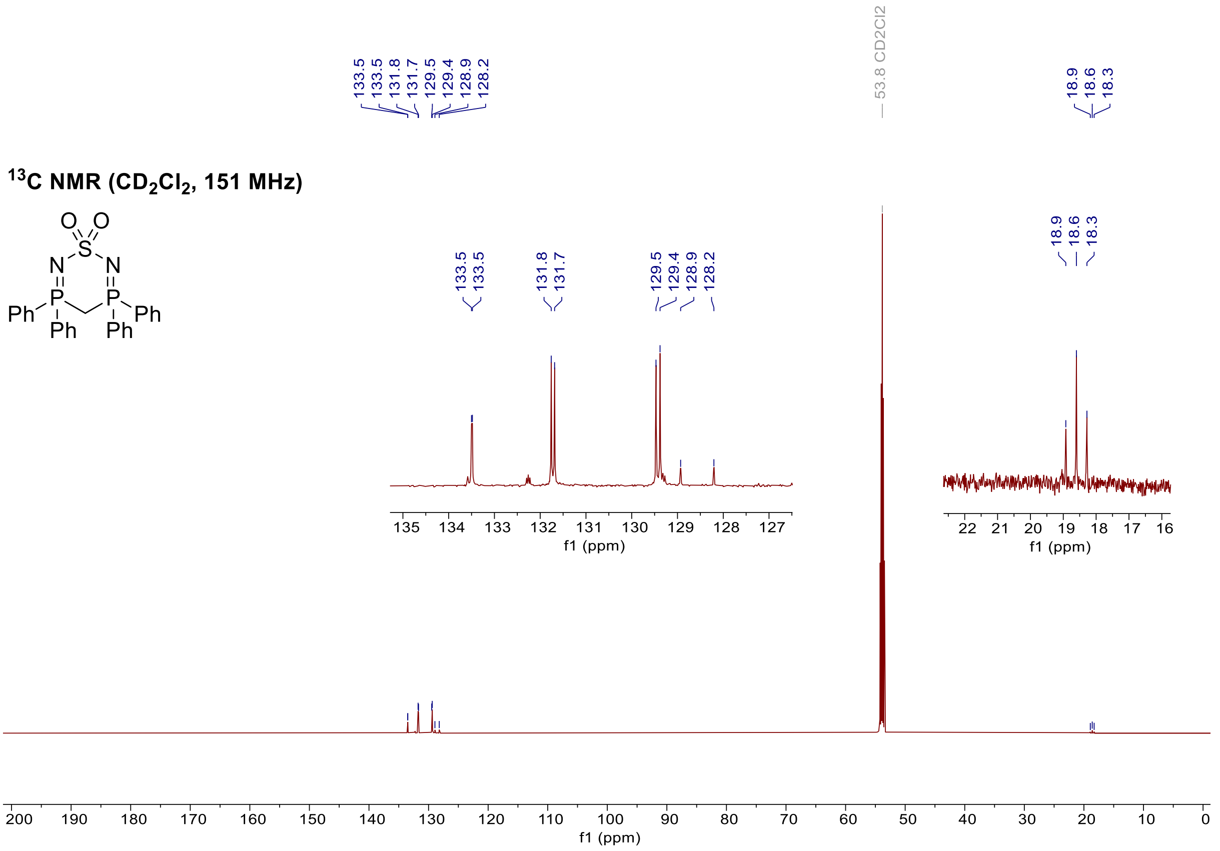

^{31}P NMR (CD_2Cl_2, 162 MHz)

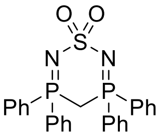

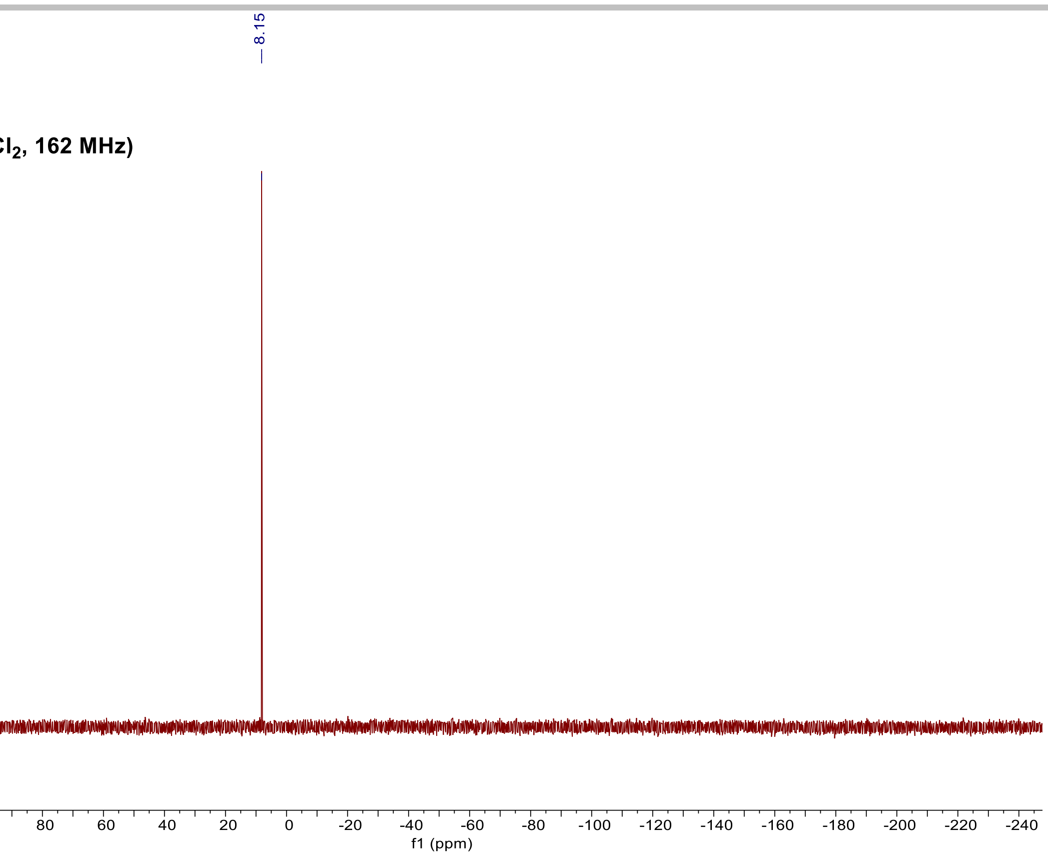

8 Crystallographic Data

4-Amino-*N*-(triphenyl-λ^5-phosphanylidene)benzenesulfonamide (16)

Suitable X-ray quality crystals were grown by dissolving the product in a minimal amount of dichloromethane and layering the solution with cyclohexane. The growth occurred due to slow solvent diffusion.

The crystal structure of **16** is depicted in Figure S5, including two slightly different molecular structures of **16** with different torsions along the N20-S21 bond and a CH_2Cl_2 solvent molecule. The torsion angle of P1-N20-S21-C24 is -67.6(4)° for molecule **16A** and 101.3(3)° for **16B**. Figure S6 visualizes the difference between the two structures **16A** and **16B**.

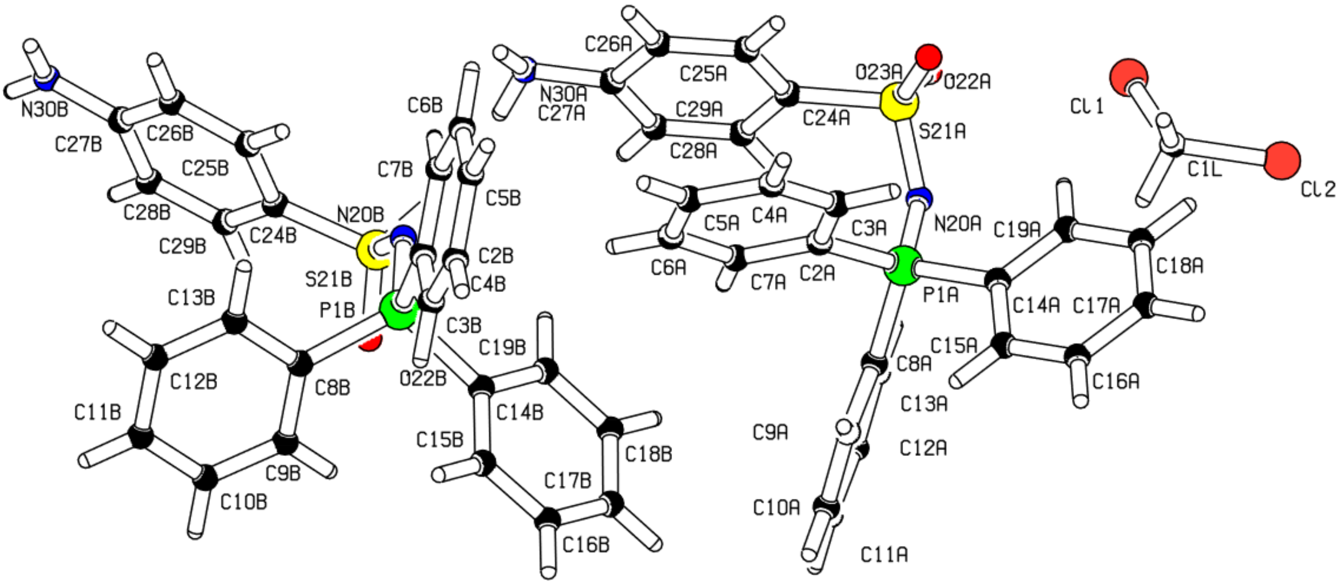

Figure S5: Platon plot of the crystal structures of 4-amino-*N*-(triphenyl-λ^5-phosphanylidene)benzenesulfonamide (**16**), with a solvent molecule (CH_2Cl_2). Two slightly different molecules were observed, differing in torsion along the N20-S21 bond.

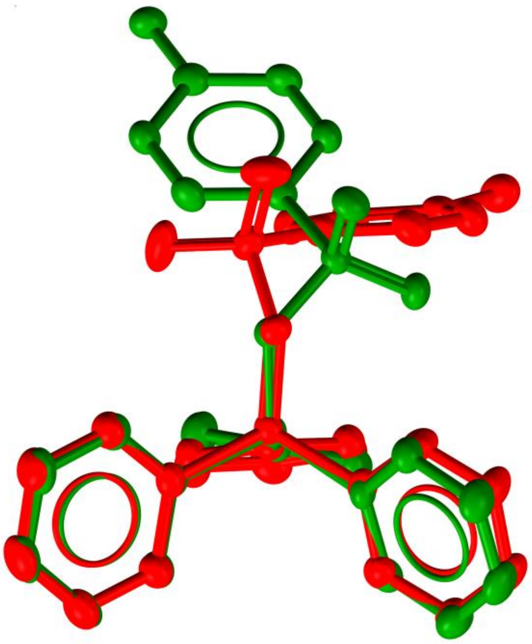

Figure S6: Visualization of the difference between the two observed molecular structures for **16** by overlapping molecules **16A** and **16B**.

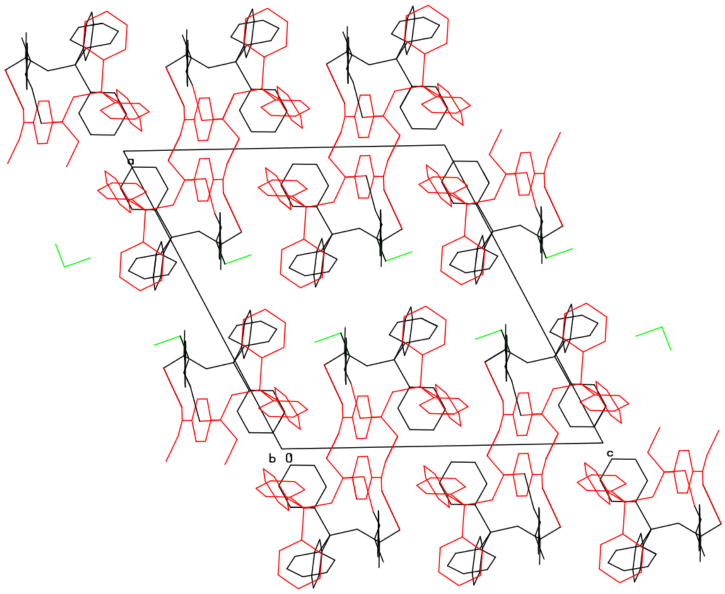

Figure S7: Part of the packing diagram of **16**. View along the b-axis. The different molecules **16A** and **16B** are marked in different colors.

Identification Code	**16**
CCDC Deposition Number	2330430
Empirical formula	$C_{49}H_{44}Cl_2N_4O_4P_2S_2$
Moiety formula	$2(C_{24}H_{21}N_2O_2PS), CH_2Cl_2$
Formula weight	949.84
Temperature	120(2) K
Wavelength, radiation type	0.71073Å, MoKα
Diffractometer	STOE IPDS 2T
Crystal system, Space Group	Monoclinic, P 2_1/c, (14)
Unit Cell Dimensions	a = 19.1019(10) Å α = 90°<br>b = 14.7468(9) Å β = 116.927(4)°<br>c = 18.1905(9) Å γ = 90°
Volume	4568.6(5) $Å^3$
Number of reflections	23016
and range used for lattice parameters	2.50° <=θ<= 28.23°
Z	4
Density (calculated)	1.381 Mg/m^3
Absorption coefficient	0.354 mm^{-1}
Absorption correction	Integration
Max. and min. transmission	0.9787 and 0.8097
F(000)	1976
Crystal size, color and form	0.050 x 0.420 x 0.640 mm^3, colorless plate
Theta range for data collection	2.632 to 28.056°.
Index ranges	-25<=h<=25, -18<=k<=19, -20<=l<=23
Number of reflections collected:	21870
independent	10838 [R(int) = 0.0575]
observed [I>2sigma(I)]	7004
Completeness to theta = 25.2°	99.6 %
Refinement method	Full-matrix least-squares on F^2
Data / restraints / parameters	10838 / 0 / 580
Goodness-of-fit on F^2	1.141
Final R indices [I>2sigma(I)]	R1 = 0.0850, wR2 = 0.1493
R indices (all data)	R1 = 0.1457, wR2 = 0.1791
Largest diff. peak and hole	0.518 and -0.628 $eÅ^{-3}$

3,3,5,5-Tetraphenyl-4*H*-1,2,6,3λ⁵,5λ⁵-thiadiazadiphosphanine-1,1-dioxide (23)

Suitable X-ray quality crystals were grown from a saturated acetonitrile solution of the compound layered with water. The growth occurred due to slow solvent diffusion.

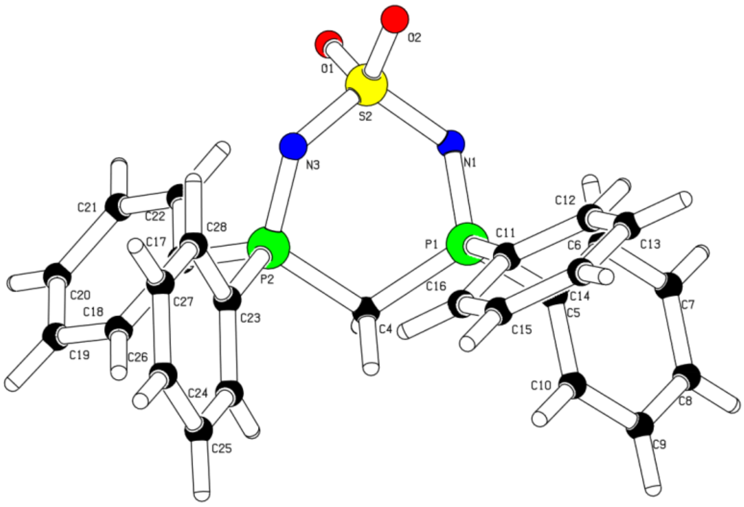

Figure S8: Platon plot of the crystal structure of 3,3,5,5-tetraphenyl-4*H*-1,2,6,3λ⁵,5λ⁵-thiadiazadiphosphanine-1,1-dioxide (**23**).

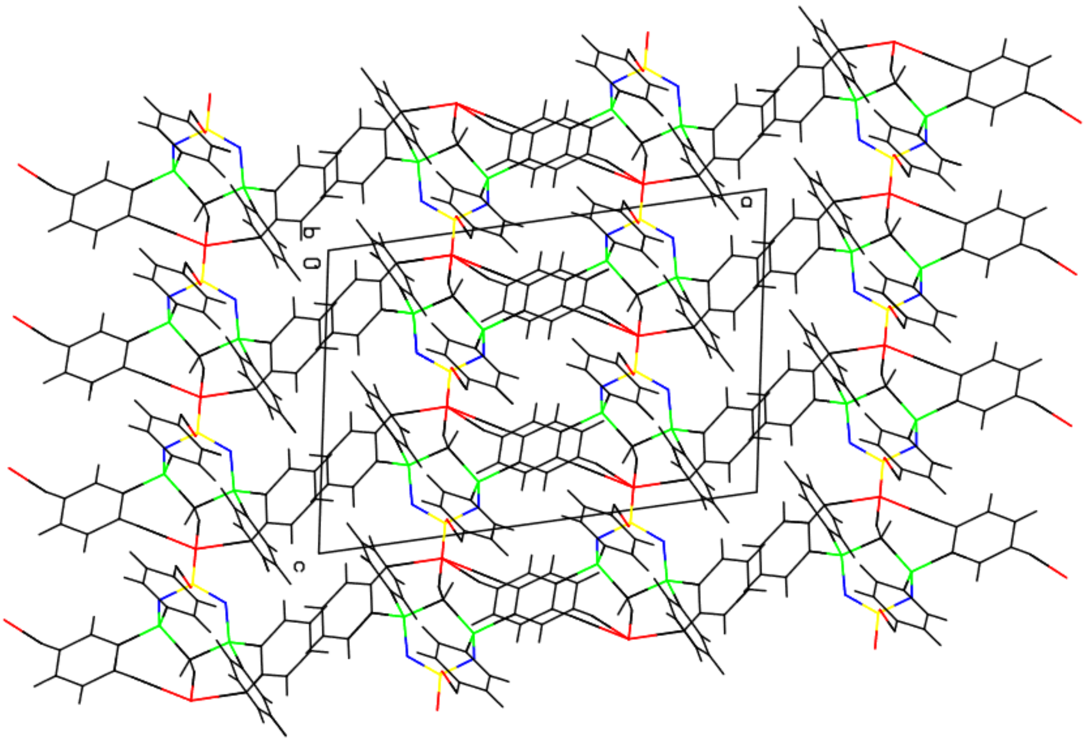

Figure S9: Part of the packing diagram of **23**. View along the b-axis.

Identification Code	**23**
CCDC Deposition Number	2330431
Empirical formula	$C_{25}H_{22}N_2O_2P_2S$
Moiety formula	$C_{25}H_{22}N_2O_2P_2S$
Formula weight	476.44
Temperature	120(2) K
Wavelength, radiation type	0.71073Å, MoKα
Diffractometer	STOE IPDS 2T
Crystal system, Space Group	Monoclinic, P 2_1/c, (14)
Unit Cell Dimensions	a = 17.4017(8) Å α = 90°<br>b = 10.5928(5) Å β = 99.796(4)°<br>c = 11.9463(6) Å γ = 90°
Volume	2169.99(18) $Å^3$
Number of reflections	9775
and range used for lattice parameters	2.59° <=θ<= 28.40°
Z	4
Density (calculated)	1.458 Mg/m^3
Absorption coefficient	0.324 mm^{-1}
Absorption correction	None
F(000)	992
Crystal size, color and form	0.030 x 0.100 x 0.160 mm^3, colorless block
Theta range for data collection	2.587 to 27.942°.
Index ranges	-22<=h<=22, -13<=k<=12, -15<=l<=15
Number of reflections collected:	10534
independent	5136 [R(int) = 0.0465]
observed [I>2sigma(I)]	3702
Completeness to theta = 25.2°	99.7 %
Refinement method	Full-matrix least-squares on F2
Data / restraints / parameters	5136 / 0 / 289
Goodness-of-fit on F^2	1.132
Final R indices [I>2sigma(I)]	R1 = 0.0632, wR2 = 0.1144
R indices (all data)	R1 = 0.1029, wR2 = 0.1329
Largest diff. peak and hole	0.374 and -0.447 $eÅ^{-3}$

9 References

(1) Sheldrick, G. M. Crystal structure refinement with *SHELXL*. *Acta Cryst.* **2015**, *C71*, 3–8.

(2) Gütz, C.; Klöckner, B.; Waldvogel, S. R. Electrochemical Screening for Electroorganic Synthesis. *Org. Process Res. Dev.* **2016**, *20*, 26–32.

(3) IKA-Werke GmbH & CO. KG. *Screening System Package (8 Cells)*. https://www.ika.com/en/Products-LabEq/Screening-System-pg913/Screening-System-Package-(8-Cells)-40003642/ (accessed 2023-10-09).

(4) Merck KGaA. *SynLectro™ Electrolysis Platform*. https://www.sigmaaldrich.com/DE/de/technical-documents/technical-article/chemistry-and-synthesis/organic-reaction-toolbox/synlectro-electrolysis-platform (accessed 2022-09-27).

(5) Elsler, B.; Wiebe, A.; Schollmeyer, D.; Dyballa, K. M.; Franke, R.; Waldvogel, S. R. Source of Selectivity in Oxidative Cross-Coupling of Aryls by Solvent Effect of 1,1,1,3,3,3-Hexafluoropropan-2-ol. *Chem. Eur. J.* **2015**, *21*, 12321–12325.

(6) Hollóczki, O.; Berkessel, A.; Mars, J.; Mezger, M.; Wiebe, A.; Waldvogel, S. R.; Kirchner, B. The Catalytic Effect of Fluoroalcohol Mixtures Depends on Domain Formation. *ACS Catal.* **2017**, *7*, 1846–1852.

(7) Hollóczki, O.; Macchieraldo, R.; Gleede, B.; Waldvogel, S. R.; Kirchner, B. Interfacial Domain Formation Enhances Electrochemical Synthesis. *J. Phys. Chem. Lett.* **2019**, *10*, 1192–1197.

(8) Motiwala, H. F.; Armaly, A. M.; Cacioppo, J. G.; Coombs, T. C.; Koehn, K. R. K.; Norwood, IV, V. M.; Aubé, J. HFIP in Organic Synthesis. *Chem. Rev.* **2022**, *122*, 12544–12747.

(9) Röckl, J. L.; Schollmeyer, D.; Franke, R.; Waldvogel, S. R. Dehydrogenative Anodic C–C Coupling of Phenols Bearing Electron-Withdrawing Groups. *Angew. Chem. Int. Ed.* **2020**, *59*, 315–319.

(10) Hayashi, T.; Kawai, M.; Tokunaga, N. Asymmetric Synthesis of Diarylmethyl Amines by Rhodium‐Catalyzed Asymmetric Addition of Aryl Titanium Reagents to Imines. *Angew. Chem. Int. Ed.* **2004**, *43*, 6125–6128.

(11) Davis, F. A.; Towson, J. C.; Weismiller, M. C.; Lal, S.; Carroll, P. J. Chemistry of Oxaziridines. 11. (Camphorylsulfonyl)oxaziridine, Synthesis and Properties. *J. Am. Chem. Soc.* **1988**, *110*, 8477–8482.

(12) Hasegawa, T.; Yamamoto, H. A Practical Synthesis of Optically Active (*R*)-2-Propyloctanoic Acid: Therapeutic Agent for Alzheimer’s Disease. *Bull. Chem. Soc. Jpn.* **2000**, *73*, 423–428.

(13) Han, W.; Su, J.; Mo, J.-N.; Zhao, J. Photoredox Catalytic Phosphine-Mediated Deoxygenation of Hydroxylamines Enables the Construction of *N*-Acyliminophosphoranes. *Org. Lett.* **2022**, *24*, 6247–6251.

(14) Monkowius, U. V.; Nogai, S.; Schmidbaur, H. Unsuccessful/successful attempts to produce penta(heteroaryl)-phosphoranes/-arsoranes R_5E (E = P, As; R = 2-furyl, 2-thienyl). *Dalton Trans.* **2004**, 1610–1617.

(15) Takeda, Y.; Kawai, H.; Minakata, S. PCy_3-Catalyzed Ring Expansion of Aziridinofullerenes with CO_2 and Aryl Isocyanates: Evidence for a Two Consecutive Nucleophilic Substitution Pathway on the Fullerene Cage. *Chem. Eur. J.* **2013**, *19*, 13479–13483.

(16) Bruce, M. I.; Burgun, A.; George, J.; Nicholson, B. K.; Parker, C. R.; Skelton, B. W.; Scoleri, N.; Sumby, C. J.; Zaitseva, N. N. Some reactions of azides with diynyl-bis(phosphine)ruthenium-cyclopentadienyl complexes. *J. Organomet. Chem.* **2015**, *797*, 185–193.

(17) Pomerantz, M.; Chou, W.-N.; Witczak, M. K.; Smith, C. G. Substituent Effects on the ^{31}P, ^{15}N, and ^{13}C NMR Spectra of *N*-(Arylsulfonyl)-*P*,*P*,*P*-triphenylphospha-λ^5-azenes and on the ^{15}N and ^{13}C NMR Spectra of the Corresponding Arenesulfonamides. *J. Org. Chem.* **1987**, *52*, 159–163.

(18) Liu, Y.; Wang, H.; Yang, X. Copper-catalyzed imination of sulfoxides and sulfides. *Tetrahedron* **2019**, *75*, 4697–4702.

(19) Sunagawa, S.; Morisaki, F.; Baba, T.; Tsubouchi, A.; Yoshimura, A.; Miyamoto, K.; Uchiyama, M.; Saito, A. *In Situ* Generation of *N*-Triflylimino-λ^3-iodanes: Application to Imidation of Phosphines and Catalytic α-Amidation of 1,3-Dicarbonyl Compounds. *Org. Lett.* **2022**, *24*, 5230–5234.

(20) Tolstikova, L. L.; Bel’skikh, A. V.; Shainyan, B. A. Reaction of *N*-Sulfinyltrifluoromethanesulfonamide with Triphenylphosphine and Triphenylphosphine Oxide. *Russ. J. Gen. Chem.* **2010**, *80*, 1189–1192.
